# Tuning the Functionality of Self-Assembled 2D Platelets in the Third Dimension

**DOI:** 10.1021/jacs.3c08770

**Published:** 2023-11-08

**Authors:** Tianlai Xia, Zaizai Tong, Yujie Xie, Maria C. Arno, Shixing Lei, Laihui Xiao, Julia Y. Rho, Calum T. J. Ferguson, Ian Manners, Andrew P. Dove, Rachel K. O’Reilly

**Affiliations:** † School of Chemistry, University of Birmingham, Edgbaston, Birmingham, B15 2TT, U.K.; ‡ College of Materials Science and Engineering, Zhejiang Sci-Tech University, Hangzhou 310018, People’s Republic of China; § Institute of Cancer and Genomic Sciences, University of Birmingham, Edgbaston, Birmingham B15 2TT, U.K.; ∥ Department of Chemistry, University of Victoria, Victoria, BC V8P 5C2, Canada; ⊥ Centre for Advanced Materials and Related Technology (CAMTEC), University of Victoria, 3800 Finnerty Road, Victoria, BC V8P 5C2, Canada

## Abstract

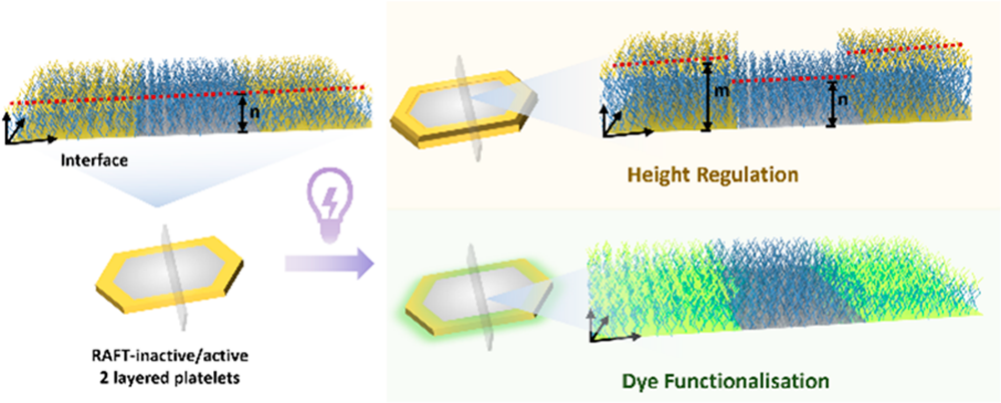

The decoration of 2D nanostructures using heteroepitaxial growth is of great importance to achieve functional assemblies employed in biomedical, electrical, and mechanical applications. Although the functionalization of polymers before self-assembly has been investigated, the exploration of direct surface modification in the third dimension from 2D nanostructures has, to date, been unexplored. Here, we used living crystallization-driven self-assembly to fabricate poly(*ε*-caprolactone)-based 2D platelets with controlled size. Importantly, surface modification of the platelets in the third dimension was achieved by using functional monomers and light-induced polymerization. This method allows us to selectively regulate the height and fluorescence properties of the nanostructures. Using this approach, we gained unprecedented spatial control over the surface functionality in the specific region of complex 2D platelets.

## Introduction

Two-dimensional (2D) nanostructures, with plate-like morphology, have attracted considerable interest due to many of their unique characteristics of large surface area, adjustable aspect ratio, defined shape (i.e., hexagon, rectangle, spindle), layered structure, packing ability, and their modifiable optical, medical, catalytic, and mechanical properties.^1−5^ These advantages have been employed in many applications, for example, as disease-targeted carriers,^6^ electronic circuit templates,^7^ and local-environment monitors/reactors.^8^

Crystallization-driven self-assembly (CDSA) has emerged as a powerful strategy to create 2D nanostructures of various sizes, contributing crystalline core promoted self-assembly, which allows flat platelet structures to be formed due to the low interfacial tension of the crystallized block.^9−18^ For elevating the uniformity of assemblies, Manners and Winnik first reported the concept of “living” CDSA for 1D and 2D structures, where they separated the crystallite nucleation and growth processes, reducing the polydispersity of nanostructures formed.^19,20^ The well-defined assemblies achieved through CDSA are versatile for a broad range of applications,^21−25^ from drug delivery,^26−29^ electronic devices,^30−33^ and reactor substrates^34−37^ to emulsion stabilizers.^38^

**Scheme 1:**
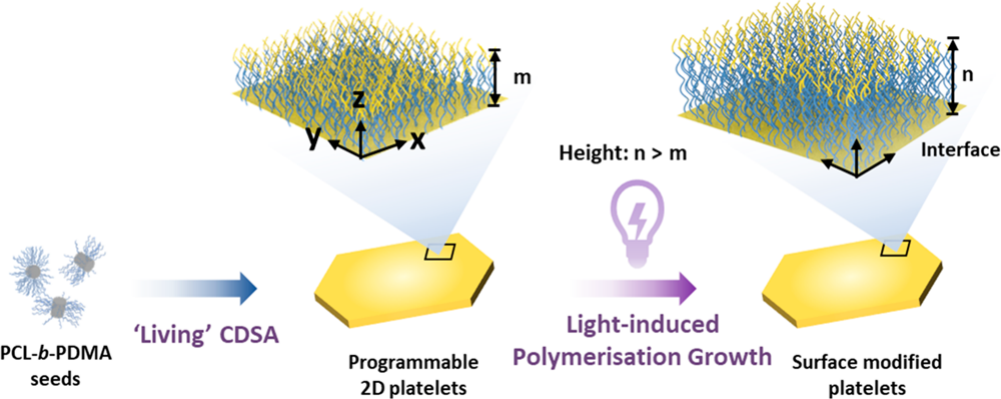
Route of Preparation of the Highly Controlled 2D PCL-Based Platelets via Living CDSA and Surface Modification on Platelets by Light-Induced Polymerization Growth

Currently, 2D platelets with various functionalities are built using prefunctionalized polymers. However, there are two main drawbacks using this method; first, the functionality within the 2D structure is limited to the chemistry of the prefunctionalized polymers, and second, the incorporation of functional polymers can affect the self-assembly and in turn the nanostructures that they adopt. To date, there are very few examples that use living CDSA to prepare 3D assemblies that includes seeded growth from a surface^39^ and self-assembly in solution.^40−42^ However, modification in the third dimension from a flat 2D surface has not been investigated before, which allows the formation of more complex structures that could be used for multiple applications. Specifically, the spatial growth from a 2D surface may enable patterning of the 2D structure in the third dimension (extending to the Z axis, not only in the XY plane), introducing variations in 3D topology over the 2D structure. Moreover, the selective addition of functional monomers, such as dyes, at different positions of a 2D platelet would enable the separation of different functionalities within a nanostructure. To facilitate the formation of a range of different structures, this modification should be undertaken postassembly using a methodology that does not disrupt the preformed nanomaterial. Currently the epitaxial decoration of anisotropic assemblies is very challenging due to the limited fabrication approaches that can be employed while maintaining the integrity of the assembled structure.^43−45^

Surface modification strategies such as spin-coating, physical/chemical deposition, heat treatment, and ion implantation have long been utilized to alter the properties of synthetic nanostructures by introducing different functional groups or layers on the surface.^46−48^ However, these conventional approaches have inevitable limitations. For example, heat treatment is unsuitable for heat-sensitive materials, and spin-coating and deposition approaches limit surface interaction and adhesion durability, which hinder the selective and spatial control over the resulting structures.^49,50^ To overcome these limitations, surface-initiated modification has been explored as an alternative strategy that provides precise control over the density and chemical composition of the modified surface. For this, initiating groups are immobilized on the surface, enabling the growth of the polymer chain from the nanostructure. However, only a few examples of precise modifications from surfaces (e.g., silicon wafer, metal, glass) have been investigated and these have mainly been limited to fixed, rigid, and unadjusted substrates.^39,51−54^

Here, we report an efficient method to modify 2D CDSA platelets in the third dimension, altering their topology and functionality (Scheme 1). We demonstrate the fabrication of multilayered 2D platelets with different functionality (corona or end group) that can be further surface modified via light-induced polymerization. Fluorescent monomers can also be attached to platelet surfaces using the same strategy, allowing visible tracking of the 2D nanostructured assemblies. Importantly, we can modify specific regions of the 2D platelet in the third dimension by altering the height or fluorescence properties of the surface.

## Results and Discussion

### Controlling Platelet Growth

**Figure 1:**
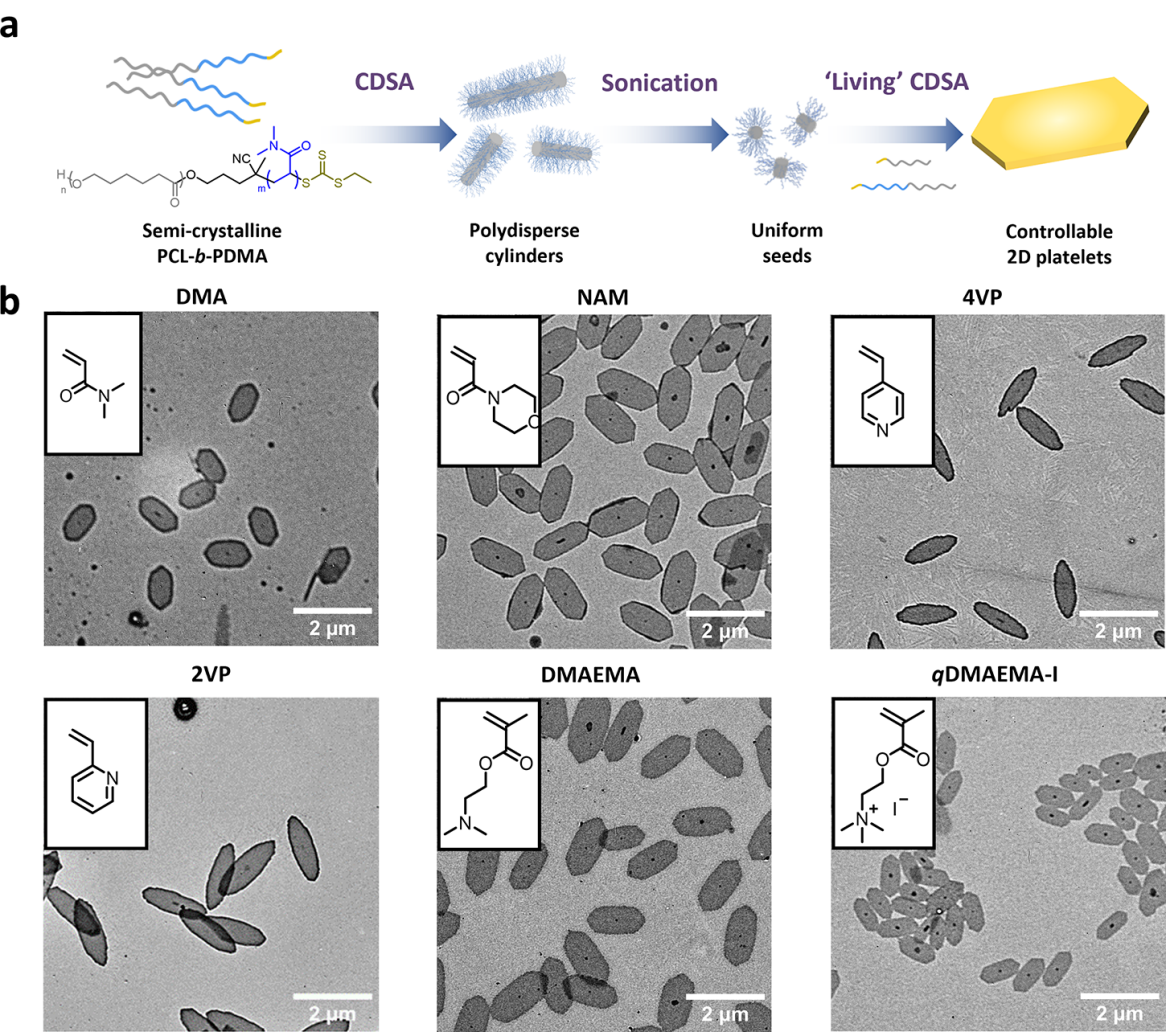
Precise control of 2D platelet growth. (a) The synthesis route for PCL/PCL-*b*-PDMA platelets by a living CDSA approach. (b) 2D platelets composed of PCL/PCL-*b*-PX (X represents types of corona including DMA, NAM, 4VP, 2VP, DMAEMA, *q*DMAEMA-I). Scale bar = 2 μm. The crystalline seeds are prepared from the PCL-*b*-PDMA cylinder. Typical living CDSA procedure: 10 μL of unimer solution (10 mg/mL, PCL/PCL-*b*-PX in a 1:1 w/w ratio dissolved in THF) was added to 1 mL of seed solution (0.01 mg/mL in ethanol).

Poly(*ε*-caprolactone)-*b*-poly(*N*,*N*-dimethylacrylamide) (PCL-*b*-PDMA) was synthesized using a hydroxy-terminated dual-head reversible addition/fragmentation chain transfer (RAFT) agent, which could undergo ring-opening polymerization (ROP) followed by RAFT polymerization (see Schemes S1 and S2, Figures S1–S5 for details).^55^ Following a reported procedure, the resulting PCL_50_-*b*-PDMA_198_ block copolymer (BCP) was used to form polydisperse cylinders via CDSA, then sonication to obtain small crystalline seeds for further living CDSA (Figures 1a, S6, and S7), to form 2D platelets (seeds located on the center of platelets and parallel to the long axis, Figure S8).^55^ The addition of unimers (PCL/PCL-*b*-PDMA) to these preformed seeds led to controlled growth of 2D platelets. The presence of PCL homopolymer directs the epitaxial growth toward 2D structures as expected, while a preference for 1D cylindrical structures is observed when only PCL-*b*-PDMA is used.^44^ The ratio of the homopolymer to block copolymer in the unimer solution governs the eventual size of the platelet. By increasing PCL content (*m*_PCL_:*m*_PCL-*b*-PDMA_ from 0.25 to 5.00 wt %), the average size of platelets increased in length from ca. 770 to 1462 nm (Figure S9). Notably, the dimensions of the platelets could be controlled by varying the unimer-to-seed ratio (*m*_PCL_:*m*_PCL-*b*-PDMA_ = 1:1). This was characterized by transmission electron microscopy (TEM, Figure 1b), atomic force microscopy (AFM, Figure S10), and confocal laser scanning microscopy (CLSM, Figure S10). A linear relationship between the measured area and unimer-to-seed mass ratio indicated predictable epitaxial growth of 2D platelets that could be observed up to a ratio of 80 (Figure S10). However, the aspect ratio (ratio of length to width) remained relatively stable at around 1.85 (1.77–1.91) across the range of unimer-to-seed ratios. Low dispersity (<1.02) was observed for all 2D platelets (Table S1).

To investigate the influence of the corona block, different functional block copolymers (PCL-*b*-PX, where X represents types of corona) were incorporated into the 2D platelets. Using a range of functional monomers, such as 4-acryloylmorpholine (NAM), 4-vinylpyridine (4VP), 2-vinylpyridine (2VP), 2-(dimethylamino)ethyl methacrylate (DMAEMA), and quaternized DMAEMA-I, block copolymers with various chemistries were synthesized via RAFT polymerization (Schemes S4 and S5, Figures S14–S19, Table S2). Interestingly, platelets with different coronas were shown to adopt different morphologies. In particular, the vinylpyridine (P2VP and P4VP)-based coronas lead to spindle-like nanostructures (Figure 1b). The incorporation of these BCPs did not adversely affect the living CDSA process; all corona systems generate well-defined 2D platelets at various unimer-to-seed ratios of 5, 10, 15, and 20 (Figures S20–S24).

### Multilayer 2D Platelet Construction

**Figure 2:**
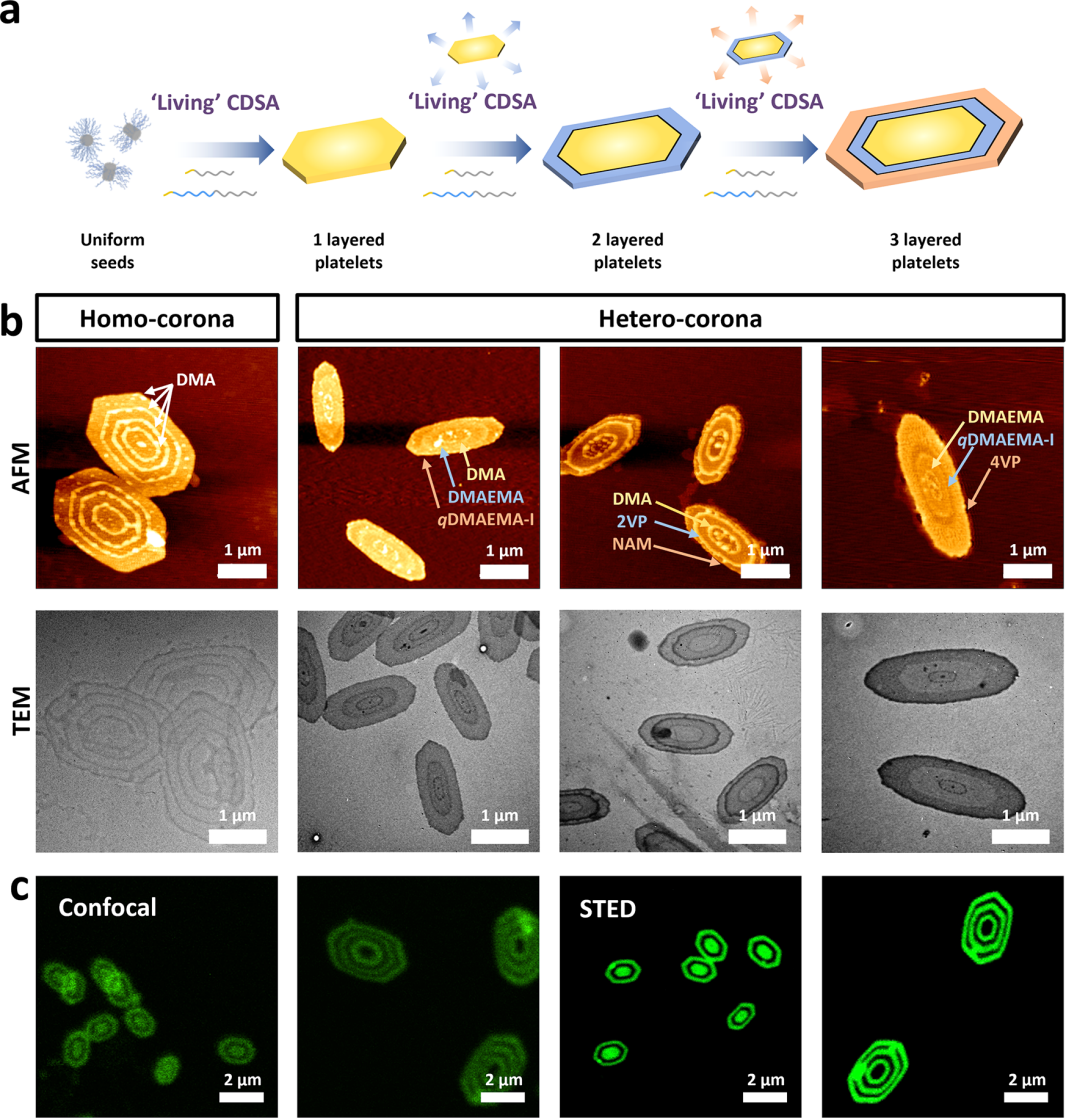
Multilayer platelet construction. (a) Mechanism of preparing multilayered platelets by sequential seeded growth. (b) AFM and TEM images of quadruple-layered homocorona platelets (four-layered PCL/PCL-*b*-PDMA platelets) and triple heterocorona platelets of L_DMA_-L_DMAEMA_-L_*q*DMAEMA-I_, L_DMA_-L_2VP_-L_NAM_, and L_DMAEMA_-L_*q*DMAEMA-I_-L_4VP_ (L_DMA_ represents a PCL/PCL-*b*-PDMA layer) via sequential seeded growth. Scale bar: 1 μm. TEM images were stained with 1 wt % uranyl acetate in water. (c) CLSM and STED images of fluorescent modified multilayered platelets by sequential epitaxial growth of fluorescent or nonfluorescent modified PCL/PCL-*b*-PDMA blending unimers. Scale bar = 2 μm.

Having accomplished 2D epitaxial growth using 1D short seeds, we investigated whether a secondary layer could be grown from prepared platelets (Figure 2a). Double-layered platelets containing two regions, an inner layer and an outer layer, can be generated by the sequential addition of blending unimers (*m*_homopolymer_:*m*_block copolymer_ = 1:1, Figure S25). PCL and PCL-*b*-PDMA blend unimers have a preference for growing from the exposed crystalline plane, as it is more energetically favored. Consequently, when additional unimers are introduced, they tend to deposit on the edges of PCL-based platelets rather than initiating spontaneous nucleation.^20^ Furthermore, well-defined triple-layered and quadruple-layered platelets were prepared following a similar procedure, as observed by AFM and TEM (Figures 2b, S25). To further explore heterocorona chemistry, the multilayered platelets with different coronas were targeted. All corona modifications demonstrated good compatibility with heterocoronas in a sequential seeded growth process (Figures 2b, S26).

To further prove the precision and scope of this method, the fluorescent dye aminochloromaleimide (ACM) was utilized to functionalize polymers (homopolymer PCL-ACM and block copolymer PCL-*b*-PDMA-ACM, Scheme S3, Figures S11–S13).^56^ The modified polymers can generate spatially defined fluorescent layered platelets by living CDSA. Through adjusting the addition sequences of fluorescent and nonfluorescent polymer unimers into a seed solution, various kinds of patterned platelets (dark and green areas within the “patterned platelets” correspond to nonfluorescent and fluorescent regions, respectively) were fabricated and visualized by CLSM and stimulated emission depletion (STED) microscopy (Figures 2c, S27). Using these methods, we could observe the formation of complex 2D structures with a spatially confined surface functionality. Moreover, no detectable diffusion of luminescent polymers across the area of the 2D platelets was observed over time (2, 10, and 35 days, Figure S28). Furthermore, this shows that no chain transformation in the platelet local environment occurred through this process.

### *In Situ* Surface Modification

**Figure 3:**
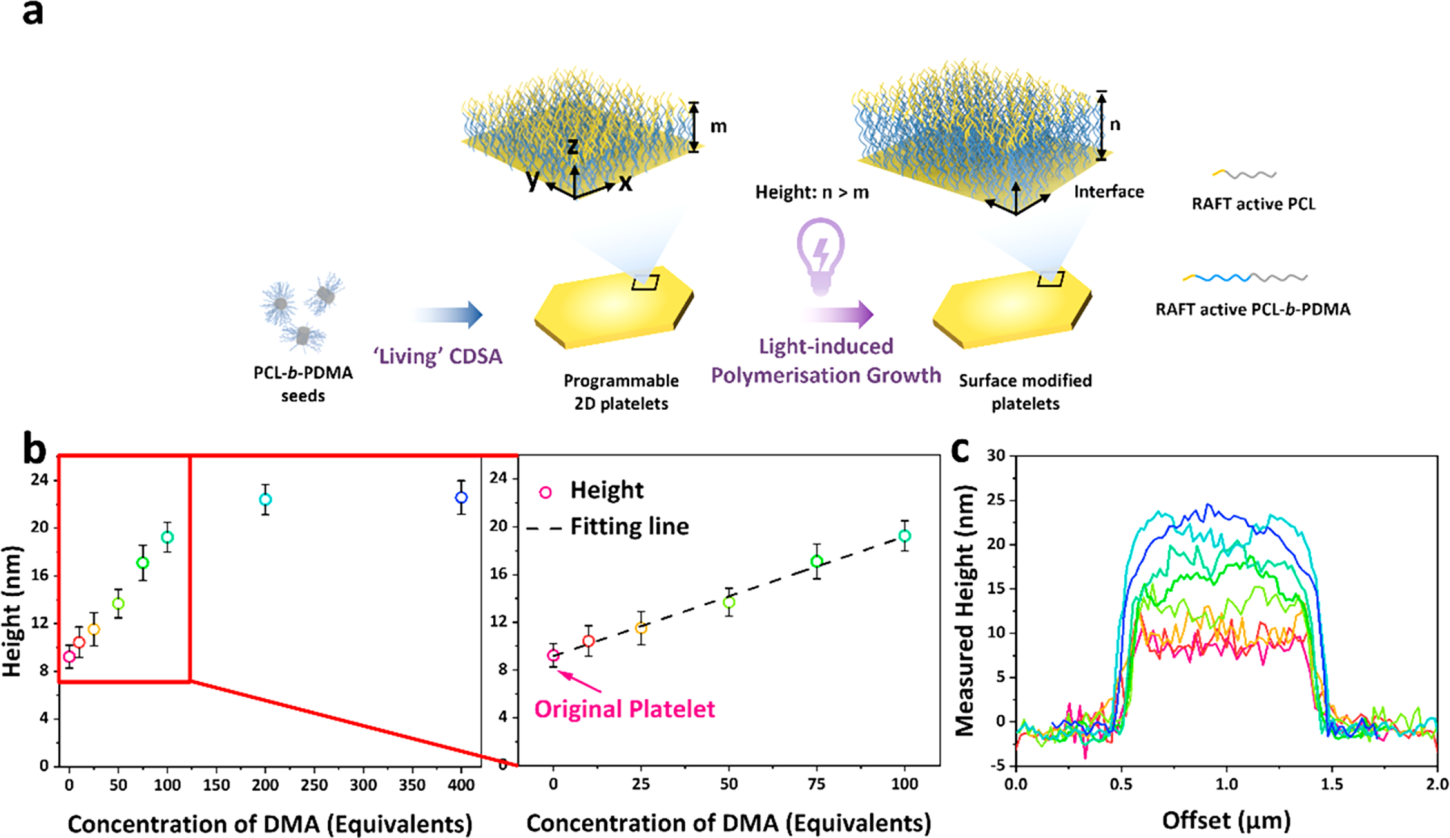
*In situ* surface modification via light-induced polymerization (photoiniferter polymerization). (a) Strategy of surface modification of CDSA platelets prepared via light-induced polymerization growth. Typical light-induced polymerization procedure: prepared platelets (in ethanol) and monomer mixed solution was degassed under a nitrogen flow for 30 min in an ice bath, then was placed in the UV cross-linker and irradiated with 405 nm light under 25 °C for 4 h. (The role of PCL-*b*-PDMA is to act as a stabilizer, enhancing colloidal stability in the solvent and preventing precipitation, also no change of Tyndall effect before and after cooling as shown in Figure S30.) (b) Relationship of platelet height against the concentrations of DMA monomer (0, 10, 25, 50, 75, 100, 200, and 400 mass equivalents to platelets mass) by photoiniferter polymerization. (c) AFM height profiles of platelets reacted with different concentrations of DMA.

The surface modification of 2D PCL-based platelets was investigated in order to modify the structure in the third dimension to enhance the tunability of the nanostructures (Figure 3a). The assemblies were observed by AFM, exhibiting a uniform height of 10 nm across the platelets (Figure S29). Photoiniferter polymerization of DMA on the surface of platelets was carried out under UV irradiation and a nitrogen atmosphere. Considering the degradable nature of PCL, prolonged radiation time may induce photodegradation of the platelets;^57,58^ therefore, the UV radiation was minimized to 4 h. Following dialysis to remove unreacted monomer, the height change of platelets was observed to 20 nm (100 mass equivalents of DMA to platelets), and platelet morphology was maintained by AFM analysis, revealing a successful surface functionalization via *in situ* light-induced polymerization. Moreover, a clear shift in the molecular weight of the bimodal distributions was confirmed by SEC, with good overlap of the refractive index and UV traces (λ_abs_ = 309 nm, Figure S29), showing the successful polymerization and retention of the RAFT end group. To further confirm the applicability of this approach, the aminobromomaleimide methacrylate (ABMMA)-based fluorescent monomer was polymerized to the surface of platelets via the same procedure (Scheme S6). As observed by CLSM, the overlap of fluorescent and bright field channels confirmed that the fluorescent dye was distributed uniformly on the surface of the platelets (Figure S31).

After successful surface modification with a homogeneous monomer and fluorescent monomer by light-induced polymerization, we sought to explore whether the platelet height could be precisely tuned by varying the equivalents of the monomer added. Arrangements from 10 to 400 mass equivalents of DMA were used for the photoiniferter polymerization process. AFM analysis confirmed that the height of the platelets changed as expected and increased in a linear relationship between platelet height and monomer concentration, ranging from 11 to 20 nm with 10–100 mass equivalents of DMA (Figures 3b,c, S32). Interestingly, when the concentration of DMA was increased to higher equivalents (more than 200 mass equiv), the height remained at ca. 22 nm (Figure S32). Furthermore, NMR spectroscopic analysis showed an increase in the ratio of the PDMA block to the PCL block, indicating the DP of PDMA increased. It exhibited a linear curve below 200 equiv and then plateaued, which is associated with the height results (relationship of DMA concentration and platelet height, Figures S34 and 35, Table S3). This phenomenon may be ascribed to three reasons: (1) the chain ends aggregating on the surface of the platelets or the active polymer chains becoming bulkier, limiting the active RAFT group’s ability to further react with the free monomer; (2) the degradation of the RAFT agent (trithiocarbonate), especially the fragmented thiyl radical;^59^ and (3) the uncontrolled polymerization rate when [DMA]/[RAFT agent] was very high.^60^ Furthermore, the behavior of the chains standing upright and polymer chain conformation on the surface of PCL-based platelets is intricately linked to factors such as grafting density, molecular weight, and mean square radius of gyration (*R*_g_) of corona segments in ethanol, requiring further investigation in the future. Encouraged by the promising results that platelets assembled via CDSA could act as a template for surface decoration, we investigated whether heterogeneous monomers could also be applied in this system (Figures S36 and S37). Photoiniferter polymerization successfully achieved the addition of the heterogeneous DMAEMA monomer. A significant increase in molecular weight was detected by SEC analysis, requiring further investigation of the photo-induced polymerization for this system.

### Spatial Selective Modulation in the Third Dimension

**Figure 4:**
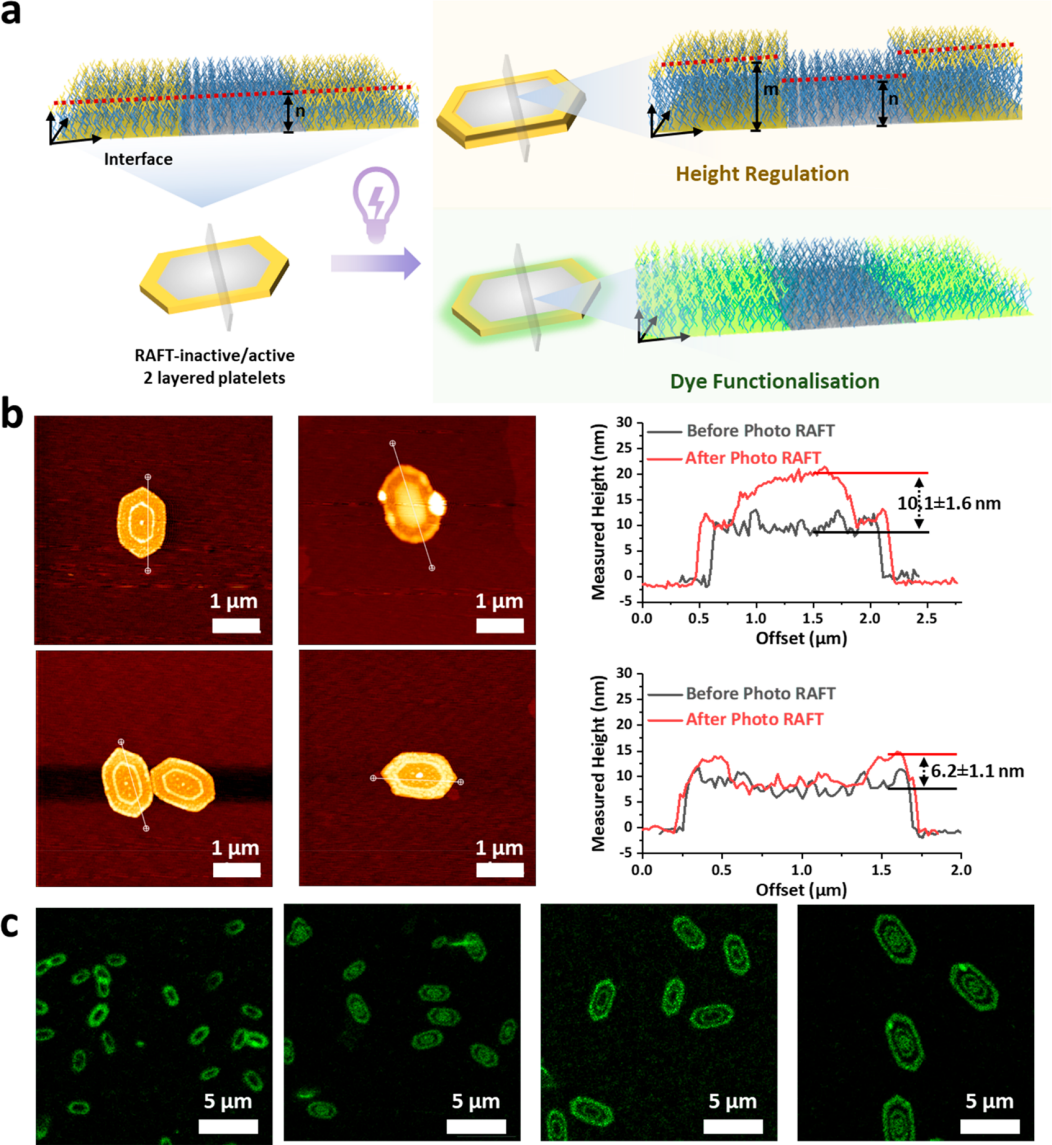
Spatial selective modulation on 2D platelets’ surface via light-induced polymerization (PET-RAFT polymerization). (a) Design of spatially selective modification on the surface of platelets. Reaction conditions: platelets, monomer, and photocatalysts (eosin Y, 0.01 equiv) mixed solution was degassed under a nitrogen flow for 30 min in an ice-bath, then was placed in the self-made light setup and irradiated with green light under 25 °C for 4 h. (b) AFM images and height profiles of double-layered platelet before and after light-induced polymerization with DMA monomer. Scale bar = 1 μm. (c) CLSM (hybrid detector) images of a multilayered platelet with 2, 3, 4, and 5 layers, respectively (left to right). Specific layers in the multilayered platelet, containing active RAFT end groups, were chain extended with the ABM dye monomer through PET-RAFT polymerization. Scale bar = 5 μm.

To satisfy more specific application scenarios such as pharmaceutical encapsulation and microelectronic devices, selective functionalization on a specific region of the platelet was investigated using *in situ* surface modification (Figure 4a). Modulating the position of the RAFT active group in the specific layer of the platelets is a facile way for postassembly surface modification, providing the chain-extending ability to postpolymerize only on the layer containing the RAFT group (Scheme S7 and Figures S38–S40). To achieve this, multilayered platelets were generated by living CDSA using alternate layers of unimers with/without RAFT agent. This offers an efficient route to construct complex hierarchical structures by selective spatial modification on the surface of 2D platelets. In this work, light-induced polymerization (photoiniferter) was used to extend the chain of the polymers that had a living chain end (Figure 4a).

Using this strategy, the ability to conduct surface polymerization is restricted to the layer containing the RAFT group. From AFM analysis, the height of the inner layer containing the RAFT group was shown to increase by about 10.1 ± 1.6 nm, while the outer layer, free from the RAFT group, remained at the original height (Figures 4b, S41, and S42). Furthermore, when the RAFT group was located in the outer layer, the height of the outer layer was shown to increase in height by 6.2 ± 1.1 nm as compared to the inner layer, which again remained the same (Figures 4b, S43, and S44). This work to our knowledge demonstrated for the first time selective surface modification from 2D platelets in the third dimension. To further demonstrate this selective modification method, a fluorescent dye was incorporated using photoiniferter polymerization to visualize the spatially defined regions of the multilayered platelets (Figure 4a). By performing photoiniferter polymerization with dye functionalized monomer, the platelets were successfully modified to emit a green fluorescence in specific regions, which allowed for their tracking using CLSM and STED. However, limited dye incorporation due to inefficient photoiniferter polymerization resulted in poor fluorescent image resolution (Figure S45). To overcome this limitation and enhance the polymerization efficiency, photoinduced electron/energy transfer RAFT (PET-RAFT) polymerization was introduced into this system (Figure S46). PET-RAFT polymerization of prepared platelets was carried out using the fluorescent monomer (10 equiv) and photocatalyst eosin Y (0.01 equiv) under green light radiation for 4 h; see the SI for details. Upon incorporation of the fluorescent monomer to the regions containing an active RAFT end group, the segregation of domains could be observed by fluorescence imaging. The centrosymmetric multilayered platelets formed by sequentially adding RAFT or RAFT end group removed polymer to a solution of seeds, and were then polymerized with fluorescent dye by PET-RAFT. The green ring-like multilayered platelets were visualized by CLSM and STED, proving the formation of a complex hierarchical structures spatially defined by post surface modification (Figure 4c).

## Conclusions

In summary, we report a living crystallization-driven self-assembly method for the formation of uniform 2D platelets by adding PCL-based homopolymer and different corona chemistry block copolymer blends with controlled spatial dimensions and uniform dispersity. Multilayered platelets were accessed by selective sequential addition of fluorescent modified PCL blends to the 1D or 2D precursors, exhibiting excellent trace tracking ability and colloidal stability with integral structural morphology. Furthermore, *in situ* light-induced (photoiniferter or PET-RAFT) polymerization was used to selectively modify and spatially functionalize the surface of the platelets using DMA or dye as a monomer. Moreover, uniform platelets with different heights were prepared by tuning the monomer concentration during the light-induced polymerization process. This exhibited good control and a linear relationship of surface height growth upto and including the addition of 100 mass equivalents of DMA. The ability of *in situ* photodecoration of the surface of 2D platelets opens the possibility of controlled selective and spatial surface functionalization of soft materials, possibly exploiting the huge diversity of applications in material science and polymer chemistry.

## Supporting Information Available

Materials, characterization, instrumentation, methods, experimental procedures, and additional data (NMR, SEC, TEM, AFM, CLSM, and STED images) (PDF)

## References

[ref1] Boott C. E.; Nazemi A.; Manners I. Synthetic Covalent and Non-Covalent 2D Materials. Angew. Chem., Int. Ed. 2015, 54 (47), 13876–13894. 10.1002/anie.201502009. 26490667

[ref2] Hailes R. L.; Oliver A. M.; Gwyther J.; Whittell G. R.; Manners I. Polyferrocenylsilanes: synthesis, properties, and applications. Chem. Soc. Rev. 2016, 45 (19), 5358–5407. 10.1039/C6CS00155F. 27348354

[ref3] Arno M. C.; Inam M.; Weems A. C.; Li Z.; Binch A. L. A.; Platt C. I.; Richardson S. M.; Hoyland J. A.; Dove A. P.; O’Reilly R. K. Exploiting the role of nanoparticle shape in enhancing hydrogel adhesive and mechanical properties. Nat. Commun. 2020, 11 (1), 1420 10.1038/s41467-020-15206-y. 32184392 PMC7078206

[ref4] MacFarlane L. R.; Shaikh H.; Garcia-Hernandez J. D.; Vespa M.; Fukui T.; Manners I. Functional nanoparticles through π-conjugated polymer self-assembly. Nat. Rev. Mater. 2021, 6 (1), 7–26. 10.1038/s41578-020-00233-4.

[ref5] He Y.; Tang Y.; Zhang Y.; MacFarlane L.; Shang J.; Shi H.; Xie Q.; Zhao H.; Manners I.; Guo J. Driving forces and molecular interactions in the self-assembly of block copolymers to form fiber-like micelles. Appl. Phys. Rev. 2022, 9 (2), 021301 10.1063/5.0083099.

[ref6] Rohaizad N.; Mayorga-Martinez C. C.; Fojtu M.; Latiff N. M.; Pumera M. Two-dimensional materials in biomedical, biosensing and sensing applications. Chem. Soc. Rev. 2021, 50 (1), 619–657. 10.1039/D0CS00150C. 33206730

[ref7] Meng Z.; Stolz R. M.; Mendecki L.; Mirica K. A. Electrically-Transduced Chemical Sensors Based on Two-Dimensional Nanomaterials. Chem. Rev. 2019, 119 (1), 478–598. 10.1021/acs.chemrev.8b00311. 30604969

[ref8] Jin H.; Guo C.; Liu X.; Liu J.; Vasileff A.; Jiao Y.; Zheng Y.; Qiao S. Z. Emerging Two-Dimensional Nanomaterials for Electrocatalysis. Chem. Rev. 2018, 118 (13), 6337–6408. 10.1021/acs.chemrev.7b00689. 29552883

[ref9] Inam M.; Cambridge G.; Pitto-Barry A.; Laker Z. P. L.; Wilson N. R.; Mathers R. T.; Dove A. P.; O’Reilly R. K. 1D vs. 2D shape selectivity in the crystallization-driven self-assembly of polylactide block copolymers. Chem. Sci. 2017, 8 (6), 4223–4230. 10.1039/C7SC00641A. 29081959 PMC5635812

[ref10] Ganda S.; Stenzel M. H. Concepts, fabrication methods and applications of living crystallization-driven self-assembly of block copolymers. Prog. Polym. Sci. 2020, 101, 101195 10.1016/j.progpolymsci.2019.101195.

[ref11] Wang J.; Lu Y.; Chen Y. Fabrication of 2D surface-functional polymer platelets via crystallization-driven self-assembly of poly(ε-caprolactone)-contained block copolymers. Polymer 2019, 160, 196–203. 10.1016/j.polymer.2018.11.053.

[ref12] Sha Y.; Rahman M. A.; Zhu T.; Cha Y.; McAlister C. W.; Tang C. ROMPI-CDSA: ring-opening metathesis polymerization-induced crystallization-driven self-assembly of metallo-block copolymers. Chem. Sci. 2019, 10 (42), 9782–9787. 10.1039/C9SC03056E. 32055347 PMC6993615

[ref13] Cha Y.; Jarrett-Wilkins C.; Rahman M. A.; Zhu T.; Sha Y.; Manners I.; Tang C. Crystallization-Driven Self-Assembly of Metallo-Polyelectrolyte Block Copolymers with a Polycaprolactone Core-Forming Segment. ACS Macro Lett. 2019, 8 (7), 835–840. 10.1021/acsmacrolett.9b00335. 33791171 PMC8009604

[ref14] Inam M.; Foster J. C.; Gao J.; Hong Y.; Du J.; Dove A. P.; O’Reilly R. K. Size and shape affects the antimicrobial activity of quaternized nanoparticles. J. Polym. Sci., Part A: Polym. Chem. 2019, 57 (3), 255–259. 10.1002/pola.29195.

[ref15] Yu W.; Inam M.; Jones J. R.; Dove A. P.; O’Reilly R. K. Understanding the CDSA of poly(lactide) containing triblock copolymers. Polym. Chem. 2017, 8 (36), 5504–5512. 10.1039/C7PY01056G.

[ref16] Hurst P. J.; Rakowski A. M.; Patterson J. P. Ring-opening polymerization-induced crystallization-driven self-assembly of poly-L-lactide-block-polyethylene glycol block copolymers (ROPI-CDSA). Nat. Commun. 2020, 11 (1), 4690 10.1038/s41467-020-18460-2. 32943622 PMC7499262

[ref17] Hurst P. J.; Graham A. A.; Patterson J. P. Gaining Structural Control by Modification of Polymerization Rate in Ring-Opening Polymerization-Induced Crystallization-Driven Self-Assembly. ACS Polym. Au 2022, 2 (6), 501–509. 10.1021/acspolymersau.2c00027. 36536891 PMC9756957

[ref18] Wang J.; Zhu W.; Peng B.; Chen Y. M. A facile way to prepare crystalline platelets of block copolymers by crystallization-driven self-assembly. Polymer 2013, 54 (25), 6760–6767. 10.1016/j.polymer.2013.10.027.

[ref19] Wang X.; Guerin G.; Wang H.; Wang Y.; Manners I.; Winnik M. A. Cylindrical block copolymer micelles and co-micelles of controlled length and architecture. Science 2007, 317 (5838), 644–647. 10.1126/science.1141382. 17673656

[ref20] Qiu H.; Gao Y.; Boott C. E.; Gould O. E.; Harniman R. L.; Miles M. J.; Webb S. E.; Winnik M. A.; Manners I. Uniform patchy and hollow rectangular platelet micelles from crystallizable polymer blends. Science 2016, 352 (6286), 697–701. 10.1126/science.aad9521. 27151866

[ref21] Zhu C.; Nicolas J. (Bio)degradable and Biocompatible Nano-Objects from Polymerization-Induced and Crystallization-Driven Self-Assembly. Biomacromolecules 2022, 23 (8), 3043–3080. 10.1021/acs.biomac.2c00230. 35707964

[ref22] MacFarlane L.; Zhao C.; Cai J.; Qiu H.; Manners I. Emerging applications for living crystallization-driven self-assembly. Chem. Sci. 2021, 12 (13), 4661–4682. 10.1039/D0SC06878K. 34163727 PMC8179577

[ref23] Sayed F. A.; Eissa N. G.; Shen Y.; Hunstad D. A.; Wooley K. L.; Elsabahy M. Morphologic design of nanostructures for enhanced antimicrobial activity. J. Nanobiotechnology 2022, 20 (1), 536 10.1186/s12951-022-01733-x. 36539809 PMC9768920

[ref24] Rajak A.; Das A. Crystallization-Driven Controlled Two-Dimensional (2D) Assemblies from Chromophore-Appended Poly(L-lactide)s: Highly Efficient Energy Transfer on a 2D Surface. Angew. Chem., Int. Ed. 2022, 61 (15), e202116572 10.1002/anie.202116572. 35137517

[ref25] Zhu W.; Peng B.; Wang J.; Zhang K.; Liu L.; Chen Y. Bamboo leaf-like micro-nano sheets self-assembled by block copolymers as wafers for cells. Macromol. Biosci. 2014, 14 (12), 1764–1770. 10.1002/mabi.201400283. 25205068

[ref26] Zhang X.; Chen G.; Zheng B.; Wan Z.; Liu L.; Zhu L.; Xie Y.; Tong Z. Uniform Two-Dimensional Crystalline Platelets with Tailored Compositions for pH Stimulus-Responsive Drug Release. Biomacromolecules 2023, 24 (2), 1032–1041. 10.1021/acs.biomac.2c01481. 36700709

[ref27] Ganda S.; Wong C. K.; Biazik J.; Raveendran R.; Zhang L.; Chen F.; Ariotti N.; Stenzel M. H. Macrophage-Targeting and Complete Lysosomal Degradation of Self-assembled Two-Dimensional Poly(epsilon-caprolactone) Platelet Particles. ACS Appl. Mater. Interfaces 2022, 14 (31), 35333–35343. 10.1021/acsami.2c06555. 35895018

[ref28] Song Y.; Elsabahy M.; Collins C. A.; Khan S.; Li R.; Hreha T. N.; Shen Y.; Lin Y. N.; Letteri R. A.; Su L.; Dong M.; Zhang F.; Hunstad D. A.; Wooley K. L. Morphologic Design of Silver-Bearing Sugar-Based Polymer Nanoparticles for Uroepithelial Cell Binding and Antimicrobial Delivery. Nano Lett. 2021, 21 (12), 4990–4998. 10.1021/acs.nanolett.1c00776. 34115938 PMC8545462

[ref29] Li Z.; Zhang Y.; Wu L.; Yu W.; Wilks T. R.; Dove A. P.; Ding H. M.; O’Reilly R. K.; Chen G.; Jiang M. Glyco-Platelets with Controlled Morphologies via Crystallization-Driven Self-Assembly and Their Shape-Dependent Interplay with Macrophages. ACS Macro Lett. 2019, 8 (5), 596–602. 10.1021/acsmacrolett.9b00221. 35619371

[ref30] Yun N.; Kang C.; Yang S.; Hwang S. H.; Park J. M.; Choi T. L. Size-Tunable Semiconducting 2D Nanorectangles from Conjugated Polyenyne Homopolymer Synthesized via Cascade Metathesis and Metallotropy Polymerization. J. Am. Chem. Soc. 2023, 145 (16), 9029–9038. 10.1021/jacs.3c00357. 37040606

[ref31] Han L.; Fan H.; Zhu Y. L.; Wang M. J.; Pan F.; Yu D. P.; Zhao Y.; He F. Precisely Controlled Two-Dimensional Rhombic Copolymer Micelles for Sensitive Flexible Tunneling Devices. CCS Chem. 2021, 3 (5), 1399–1409. 10.31635/ccschem.020.202000297.

[ref32] Yang S.; Kang S. Y.; Choi T. L. Semi-conducting 2D rectangles with tunable length via uniaxial living crystallization-driven self-assembly of homopolymer. Nat. Commun. 2021, 12 (1), 2602 10.1038/s41467-021-22879-6. 33972541 PMC8110585

[ref33] Yang S.; Kang S. Y.; Choi T. L. Morphologically Tunable Square and Rectangular Nanosheets of a Simple Conjugated Homopolymer by Changing Solvents. J. Am. Chem. Soc. 2019, 141 (48), 19138–19143. 10.1021/jacs.9b10904. 31714065

[ref34] Lei S.; Tian J.; Kang Y.; Zhang Y.; Manners I. AIE-Active, Stimuli-Responsive Fluorescent 2D Block Copolymer Nanoplatelets Based on Corona Chain Compression. J. Am. Chem. Soc. 2022, 144 (38), 17630–17641. 10.1021/jacs.2c07133. 36107414

[ref35] Dong B.; Miller D. L.; Li C. Y. Polymer Single Crystal As Magnetically Recoverable Support for Nanocatalysts. J. Phys. Chem. Lett. 2012, 3 (10), 1346–1350. 10.1021/jz300434c. 26286781

[ref36] Zhou T.; Wang B.; Dong B.; Li C. Y. Thermoresponsive Amphiphilic Janus Silica Nanoparticles via Combining “Polymer Single-Crystal Templating” and “Grafting-from” Methods. Macromolecules 2012, 45 (21), 8780–8789. 10.1021/ma3019987.

[ref37] Dong B.; Zhou T.; Zhang H.; Li C. Y. Directed self-assembly of nanoparticles for nanomotors. ACS Nano 2013, 7 (6), 5192–5198. 10.1021/nn400925q. 23647410

[ref38] Inam M.; Jones J. R.; Perez-Madrigal M. M.; Arno M. C.; Dove A. P.; O’Reilly R. K. Controlling the Size of Two-Dimensional Polymer Platelets for Water-in-Water Emulsifiers. ACS Cent. Sci. 2018, 4 (1), 63–70. 10.1021/acscentsci.7b00436. 29392177 PMC5785766

[ref39] Cai J.; Li C.; Kong N.; Lu Y.; Lin G.; Wang X.; Yao Y.; Manners I.; Qiu H. Tailored multifunctional micellar brushes via crystallization-driven growth from a surface. Science 2019, 366 (6469), 1095–1098. 10.1126/science.aax9075. 31780551

[ref40] Guerin G.; Cruz M.; Yu Q. Formation of 2D and 3D multi-tori mesostructures via crystallization-driven self-assembly. Sci. Adv. 2020, 6 (16), eaaz7301 10.1126/sciadv.aaz7301. 32494620 PMC7159922

[ref41] Zhang Y.; Pearce S.; Eloi J. C.; Harniman R. L.; Tian J.; Cordoba C.; Kang Y.; Fukui T.; Qiu H.; Blackburn A.; Richardson R. M.; Manners I. Dendritic Micelles with Controlled Branching and Sensor Applications. J. Am. Chem. Soc. 2021, 143 (15), 5805–5814. 10.1021/jacs.1c00770. 33851530

[ref42] Jiang J.; Nikbin E.; Hicks G.; Song S.; Liu Y.; Wong E. C. N.; Manners I.; Howe J. Y.; Winnik M. A. Polyferrocenylsilane Block Copolymer Spherulites in Dilute Solution. J. Am. Chem. Soc. 2023, 145 (2), 1247–1261. 10.1021/jacs.2c11119. 36598864

[ref43] Ganda S.; Wong C. K.; Stenzel M. H. Corona-Loading Strategies for Crystalline Particles Made by Living Crystallization-Driven Self-Assembly. Macromolecules 2021, 54 (14), 6662–6669. 10.1021/acs.macromol.1c00643.

[ref44] Tong Z.; Xie Y.; Arno M. C.; Zhang Y.; Manners I.; O’Reilly R. K.; Dove A. P. Uniform segmented platelet micelles with compositionally distinct and selectively degradable cores. Nat. Chem. 2023, 15 (6), 824–831. 10.1038/s41557-023-01177-2. 37081206 PMC10239731

[ref45] Pearce A. K.; Wilks T. R.; Arno M. C.; O’Reilly R. K. Synthesis and applications of anisotropic nanoparticles with precisely defined dimensions. Nat. Rev. Chem. 2021, 5 (1), 21–45. 10.1038/s41570-020-00232-7. 37118104

[ref46] Zhang S.; Li W.; Luan J.; Srivastava A.; Carnevale V.; Klein M. L.; Sun J.; Wang D.; Teora S. P.; Rijpkema S. J.; Meeldijk J. D.; Wilson D. A. Adaptive insertion of a hydrophobic anchor into a poly(ethylene glycol) host for programmable surface functionalization. Nat. Chem. 2023, 15 (2), 240–247. 10.1038/s41557-022-01090-0. 36411361 PMC9899690

[ref47] Kroupa D. M.; Voros M.; Brawand N. P.; McNichols B. W.; Miller E. M.; Gu J.; Nozik A. J.; Sellinger A.; Galli G.; Beard M. C. Tuning colloidal quantum dot band edge positions through solution-phase surface chemistry modification. Nat. Commun. 2017, 8, 15257 10.1038/ncomms15257. 28508866 PMC5440806

[ref48] Lei S.; Wang X.; Li B.; Kang J.; He Y.; George A.; Ge L.; Gong Y.; Dong P.; Jin Z.; Brunetto G.; Chen W.; Lin Z. T.; Baines R.; Galvao D. S.; Lou J.; Barrera E.; Banerjee K.; Vajtai R.; Ajayan P. Surface functionalization of two-dimensional metal chalcogenides by Lewis acid-base chemistry. Nat. Nanotechnol. 2016, 11 (5), 465–471. 10.1038/nnano.2015.323. 26828848

[ref49] Karawdeniya B. I.; Damry A. M.; Murugappan K.; Manjunath S.; Bandara Y.; Jackson C. J.; Tricoli A.; Neshev D. Surface Functionalization and Texturing of Optical Metasurfaces for Sensing Applications. Chem. Rev. 2022, 122 (19), 14990–15030. 10.1021/acs.chemrev.1c00990. 35536016

[ref50] Feng C.; Wu Z. P.; Huang K. W.; Ye J.; Zhang H. Surface Modification of 2D Photocatalysts for Solar Energy Conversion. Adv. Mater. 2022, 34 (23), e2200180 10.1002/adma.202200180. 35262973

[ref51] Hetemi D.; Pinson J. Surface functionalisation of polymers. Chem. Soc. Rev. 2017, 46 (19), 5701–5713. 10.1039/C7CS00150A. 28766657

[ref52] Lee K.; Corrigan N.; Boyer C. Rapid High-Resolution 3D Printing and Surface Functionalization via Type I Photoinitiated RAFT Polymerization. Angew. Chem., Int. Ed. 2021, 60 (16), 8839–8850. 10.1002/anie.202016523. 33449437

[ref53] Corrigan N.; Yeow J.; Judzewitsch P.; Xu J.; Boyer C. Seeing the Light: Advancing Materials Chemistry through Photopolymerization. Angew. Chem., Int. Ed. 2019, 58 (16), 5170–5189. 10.1002/anie.201805473. 30066456

[ref54] Lee Y.; Boyer C.; Kwon M. S. Photocontrolled RAFT polymerization: past, present, and future. Chem. Soc. Rev. 2023, 52 (9), 3035–3097. 10.1039/D1CS00069A. 37040256

[ref55] Arno M. C.; Inam M.; Coe Z.; Cambridge G.; Macdougall L. J.; Keogh R.; Dove A. P.; O’Reilly R. K. Precision Epitaxy for Aqueous 1D and 2D Poly(epsilon-caprolactone) Assemblies. J. Am. Chem. Soc. 2017, 139 (46), 16980–16985. 10.1021/jacs.7b10199. 29078700 PMC5789388

[ref56] Xie Y.; Husband J. T.; Torrent-Sucarrat M.; Yang H.; Liu W.; O’Reilly R. K. Rational design of substituted maleimide dyes with tunable fluorescence and solvafluorochromism. Chem. Commun. 2018, 54 (27), 3339–3342. 10.1039/C8CC00772A. PMC5885783 29542762

[ref57] Ikada E. Photo- and Bio-degradable Polyesters. Photodegradation Behaviors of Aliphatic Polyesters. J. Photopolym. Sci. Technol. 1997, 10 (2), 265–270. 10.2494/photopolymer.10.265.

[ref58] Christensen P. A.; Egerton T. A.; Martins-Franchetti S. M.; Jin C.; White J. R. Photodegradation of polycaprolactone/poly(vinyl chloride) blend. Polym. Degrad. Stab. 2008, 93 (1), 305–309. 10.1016/j.polymdegradstab.2007.08.008.

[ref59] Fu Q.; McKenzie T. G.; Ren J. M.; Tan S.; Nam E.; Qiao G. G. A novel solid state photocatalyst for living radical polymerization under UV irradiation. Sci. Rep. 2016, 6, 20779 10.1038/srep20779. 26863939 PMC4749958

[ref60] Wang H.; Li Q.; Dai J.; Du F.; Zheng H.; Bai R. Real-Time and in Situ Investigation of “Living”/Controlled Photopolymerization in the Presence of a Trithiocarbonate. Macromolecules 2013, 46 (7), 2576–2582. 10.1021/ma400208j.

