# Supplementary material for: Tuning the Functionality of Self-Assembled 2D Platelets in the Third Dimension
Source: J Am Chem Soc. 2023 Nov 8;145(46):25274–82. doi: 10.1021/jacs.3c08770 (PMC10682995; doi:10.1021/jacs.3c08770)
Supplement: Supplementary file 1 — ja3c08770_si_001.pdf [file ja3c08770_si_001.pdf]

Supporting Information for

Tuning the Functionality of Self-Assembled 2D Platelets in the Third Dimension

Tianlai Xia,[1] Zaizai Tong,[1,2] Yujie Xie,[1] Maria C. Arno,[1,3] Shixing Lei,[4] Laihui Xiao,[1] Julia Y. Rho,[1] Calum T. J. Ferguson,[1] Ian Manners,[4,5] Andrew P. Dove,[1]* Rachel K. O'Reilly[1]*

[1]School of Chemistry, University of Birmingham, Edgbaston, Birmingham, B15 2TT, U.K.

[2]College of Materials Science and Engineering, Zhejiang Sci-Tech University, Hangzhou 310018, People's Republic of China

[3]Institute of Cancer and Genomic Sciences, University of Birmingham, Edgbaston, Birmingham, B15 2TT, U.K.

[4]Department of Chemistry, University of Victoria, Victoria, BC, V8P 5C2, Canada

[5]Centre for Advanced Materials and Related Technology (CAMTEC), University of Victoria, 3800 Finnerty Rd, Victoria, BC, V8P 5C2, Canada

*Email: A.Dove@bham.ac.uk

*Email: R.OReilly@bham.ac.uk

This PDF file includes:

Materials

Characterization & Instrumentation

Methods

Figure S1 to S46

Table S1 to S3

Scheme S1 to S7

References

Materials

All chemicals and solvents were used as obtained without further purification unless otherwise stated. Sodium ethanethiolate (90%), carbon disulfide (≥99%), solid iodine (≥99%), 4,4'-azobis(4-cyanovaleric acid) (ACVA, 98%), borane tetrahydrofuran complex solution (1.0 M in THF), iodomethane, nitromethane, and photocatalyst Eosin Y (EY) were purchased from Sigma Aldrich and Alfa Aesar. The synthesis of dual head CTA of 2-cyano-5-hydroxypentan-2-yl ethyl carbonotrithioate (CHPET) has been followed Varlas et al. and Kang et al. by in previous reports. CTA and diphenylphosphate (DPP, 99%, Sigma Aldrich) were dried over P_2O_5 in desiccator under static vacuum for 1 week before use. ε-caprolactone (99%, ACROS Organics) were vacuum distilled twice over CaH_2 before being introduced in the glovebox and used. 2,2'-azobis(2-methylpropionitrile) (AIBN, 98%, Sigma Aldrich) was recrystallized twice from methanol and stored at 4 °C in the dark. 1,4-dioxane (anhydrous, 99.8%), N,N-dimethyl acrylamide (DMA, 99%, contains 500 ppm monomethyl ether hydroquinone as inhibitor), 4-acryloylmorpholine (NAM, 97%, contains 1,000 ppm monomethyl ether hydroquinone as inhibitor), 4-vinylpyridine (4VP, 95%, stabilized), 2-Vinylpyridine (2VP, 97%), 2-(Dimethylamino)ethyl methacrylate (DMAEMA, 98%, contains 700-1000 ppm monomethyl ether hydroquinone as inhibitor), N-Isopropylacrylamide (NIPAm), 2-(Dimethylamino)ethyl acrylate (DMAEA, 98%, contains <2,000 ppm MEHQ as inhibitor), acrylic acid (AA, 99%, anhydrous, contains 200 ppm MEHQ as inhibitor), 2-hydroxypropyl acrylate (HPA), and methacrylic acid (MAA, 99%, contains 250 ppm MEHQ as inhibitor) were purchased from Sigma Aldrich or Fisher, and passed through a basic alumina plug immediately to remove stabilizer before use. 1-ethylpiperidine hypophosphite (EPHP) and tributylphosphine (PBu_3) were purchased from Sigma Aldrich. Solvents were purchased from Fisher Scientific. Dry solvents were used directly from a drying and degassing inert solvent tower system. Aminochloromaleimide (ACM) based fluorescent dye (4-(3-(butylamino)-4-chloro-2,5-dihydro-1H-pyrrol-1-yl)butanoic acid) and aminobromomaleimide methacrylate (ABMMA) based dye monomer (2-(3-bromo-4-(isopropylamino)-2,5-dioxo-2,5-dihydro-1*H*-pyrrol-1-yl)ethyl methacrylate) were synthesized has been followed by Xie et al. in previous reports.[1, 2]

Characterization & Instrumentation

Nuclear Magnetic Resonance (NMR). All ^{1}H and ^{13}C NMR spectra were recorded on a Bruker 400 MHz (DPX-400) spectrometer. Chemical shift (δ) are reported in parts per million (ppm) relative to internal standard tetramethylsilane (TMS) at $\delta = 0$. Samples were prepared in deuterated chloroform ($CDCl_3$) or dimethyl sulfoxide (DMSO) and referenced to residual non-deuterated signal of solvent ($CDCl_3$ @ 7.26 ppm ^{1}H-NMR and 77.2 ppm ^{13}C-NMR; DMSO @ 2.50 ppm ^{1}H-NMR). The resonance multiplicities are described as s (singlet), d (doublet), t (triplet), q (quartet) or m (multiplet).

Size Exclusion Chromatography (SEC). SEC analysis was performed on a Agilent 1260 Infinity II system fitted with RI and ultraviolet (UV) detectors (λ = 309 and 360 nm), equipped with a PLGel 3 μm (50 × 7.5 mm guard column and two PLGel 5 μm (300 × 7.5 mm) mixed-D columns, mobile phase (eluent) using $CHCl_3$ WITH 0.5% triethylamine (TEA) or DMF with 5 mM NH_4BF_4. Molecular weight (M_w) and molecular weight distributions ($Đ_M = M_w/M_n$) were calibrated against poly(methyl methacrylate) (PMMA) standards and analysed using Agilent SEC software.

Transmission Electron Microscopy (TEM). Dry state transmission electron microscopy (TEM) imaging was performed on JEOL 1400 Bio (1720/GB06) microscopy at an acceleration voltage of 80 kV. CDSA samples were deposited onto graphene oxide-coated copper grids. Then the grid was stained with an aqueous 1wt. % uranyl acetate (UA) solution to drying and microscopic analysis. For the determination of the average micelles' size at least 100 particles were analysed in each case.

Atomic Force Microscopy (AFM). AFM samples were prepared by drop casting 10 μL of assemblies in ethanol onto mica silicon wafer which treated by Sigmacote, followed by drying naturally in air. Imaging and analysis were performed on a JPK Nanowizard 4 system in quantitative imaging (QI) mode. The tips for the AFM analysis (PPP-NCHAuD) were purchased from NANOSENSORSTM, with resonance frequency (kHz) in the window of 204 - 497 and force constant in the range of 10 - 130. The AFM pictures and data were analysed and collected using the JPK Data Processing software with QI mode. For determination of the average micelles' size at least 100 particles were analysed in each case.

For micelle contour lengths, widths and areas analysis, ca. 100 micelles in several images were traced manually using the ImageJ software package developed at the US National Institute of Health. The number average micelle length (L_n) and weight average micelle length (L_w) were

calculated using blow equation from measurements of the contour lengths (L_i) of individual micelles, where N_i is the number of micelles of length L_i, and n is the number of micelles examined in each sample. The distribution of micelle lengths is characterized by both L_w/L_n.

$$L_n = \frac{\sum_{i=1}^{n} N_i L_i}{\sum_{i=1}^{n} N_i} \qquad L_w = \frac{\sum_{i=1}^{n} N_i L_i^2}{\sum_{i=1}^{n} N_i L_i}$$

The number average micelle width (W_n) and weight average micelle width (W_w) [3]were calculated using blow equation from measurements of the contour widths (W_i) of individual micelles, where N_i is the number of micelles of width W_i, and n is the number of micelles examined in each sample. The distribution of micelle widths is characterized by both W_w/W_n.

$$W_n = \frac{\sum_{i=1}^{n} N_i W_i}{\sum_{i=1}^{n} N_i} \qquad W_w = \frac{\sum_{i=1}^{n} N_i W_i^2}{\sum_{i=1}^{n} N_i W_i}$$

The number average micelle area (A_n) and weight average micelle area (A_w) were calculated using blow equation from measurements of the contour areas (A_i) of individual micelles, where N_i is the number of micelles of length A_i, and n is the number of micelles examined in each sample. The distribution of micelle lengths is characterized by both A_w/A_n.

$$A_n = \frac{\sum_{i=1}^{n} N_i A_i}{\sum_{i=1}^{n} N_i} \qquad A_w = \frac{\sum_{i=1}^{n} N_i A_i^2}{\sum_{i=1}^{n} N_i A_i}$$

Confocal Laser Scanning Microscopy (CLSM). FV3000 (Olympus) confocal microscope with a IX-81 inverted base (Olympus) and the 20x and 60x oil lens (Olympus) was used for imaging. The FV3000 system was driven with the FV31S-SW Viewer software platform (Olympus) with scan rates of 1 μs pixel^{-1} at 515 by 512 pixel^{-1} to 1024 by 1024 pixel^{-1}.

Stimulated Emission Depletion (STED) Microscopy. CLSM imaging was carried out on a Leica TCS SP8 STED 3X microsystem using 405 nm laser excitation. The fluorescence signal was detected by a Hybrid Detector (HyD, Standard mode, with observation window of 430–500 nm) after passing through an Acousto-Optical Beam Splitter (AOBS). The resulting outputs were obtained as digital false-colour images and colour coded as green. 2D nanoplatelets solution (~10 μL) was sealed between cover glasses for the imaging experiment.

UV Light Apparatus Setup. The light source for the light induced polymerization reactions (TruOpto OSV5X3CAC1E) was purchased from Rapid Electronics and had an output power of 800 mW at 12 V DC operating at a wavelength of 400 - 410 nm. This was fitted to a custom-built setup fitted with a dimmer switch for controlling the output light intensity. (**Setup shown bellowing picture**)

Green Light Apparatus Setup. The green light source for the light induced polymerization reactions was purchased from Amazon and had an output power of 60 W. This was fitted to a custom-built setup fitted with a dimmer switch for controlling the output light intensity. (**Setup shown bellowing picture**)

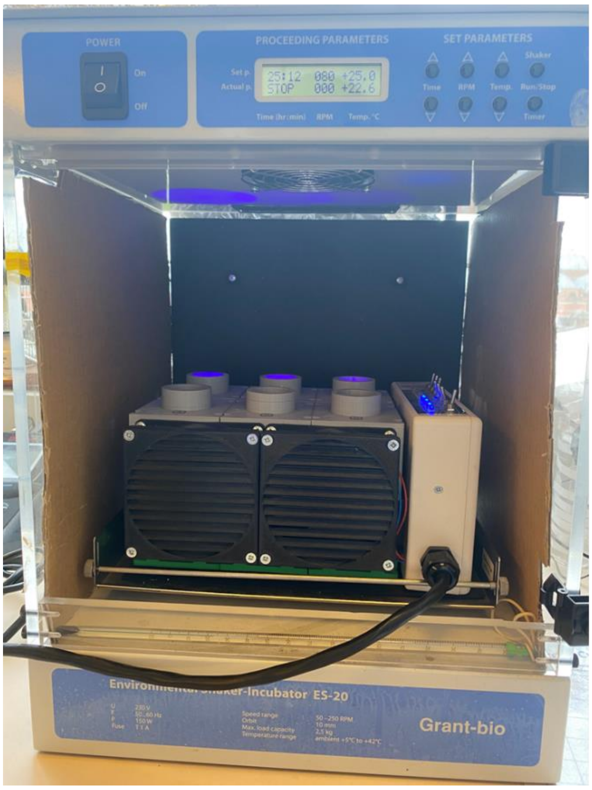

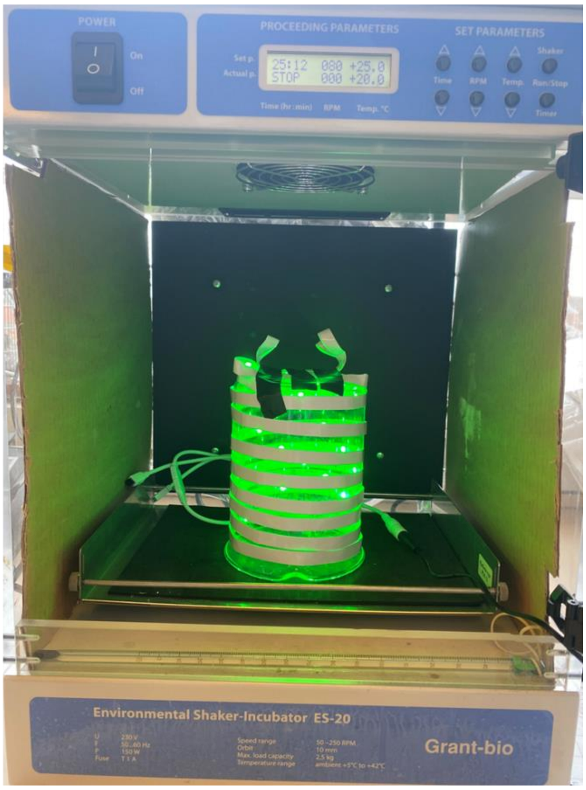

Setup	Photo-iniferter polymerization	PET-RAFT polymerization
Light	UV light (400-410 nm)	Green light (around 530 nm)
Temperature	25 degrees	25 degrees
Photocatalysts	-	Eosin Y (0.01 equiv)
Solvent	Ethanol	Ethanol

Methods

Synthesis of 4-cyano-4-(((ethylthio)carbonothioyl)thio)pentanoic acid (CEPA). Following a previously reported procedure,[4, 5] to an oven-dried round bottom flask, sodium ethanethiolate (10 g, 119 mmol, 1 equiv) was added followed by the addition of dry diethyl ether (500 mL) with the resulting solution cooled to 0 ˚C. Carbon disulfide (7.74 mL, 131 mmol, 1.1 equiv) was subsequently added dropwise over 10 min. producing a thick yellow precipitate of sodium S-ethyl trithiocarbonate. After 2 h of stirring at room temperature, solid iodine (15.1 g, 59.4 mmol, 0.5 equiv) was added and the resultant reaction mixture was stirred for a further 2 h at room temperature. The reaction mixture was then washed with sodium thiosulfate solution (1 M, 3 × 100 mL), deionized water (3 × 100 mL) and finally with saturated sodium chloride solution (3 × 100 mL). The organic phase was dried over $MgSO_4$, filtered and evaporated to remove solvent, leaving a residue of bis-(ethylsulfanylthiocarbonyl) disulfide (15.6 g, 56.8 mmol). A solution of 4,4'-azobis(4- cyanovaleric acid) (ACVA) (23.9 g, 85.2 mmol, 1 equiv) and bis-(ethylsulfanylthiocarbonyl) disulfide (15.6 g, 56.8 mmol, 0.67 equiv) in ethyl acetate (500 mL) was heated to 80 ˚C overnight at reflux under a N_2 atmosphere. After removal of the volatile solvents in vacuo, purification was carried out using silica gel column chromatography (hexane : dichloromethane = 1:3) affording 4-cyano-4-(((ethylthio) carbonothioyl)thio)pentanoic acid (CEPA) as an orange red oil (24.4 g, 97.7 mmol, 86%). ^{1}H NMR (400 MHz, $CDCl_3$): δ (ppm): 3.34 (q, 2H, SC$\boldsymbol{H_2}$$CH_3$), 2.68 (m, 2H, C(CN)($CH_3$)$CH_2C\boldsymbol{H_2}$), 2.33-2.60 (m, 2H, C(CN)(CH_3)C$\boldsymbol{H_2}$$CH_2$), 1.89 (s, 3H, C(CN)(C$\boldsymbol{H_3}$) CH_2CH_2), 1.36 (t, 3H, SCH_2C$\boldsymbol{H_3}$). ^{13}C NMR (100 MHz, $CDCl_3$): δ (ppm): 216.7 (***C***=S), 177.3 (***C***=O), 118.9 (C(***C***N)(CH_3)CH_2CH_2), 46.2 (***C***(CN)(CH_3)CH_2CH_2), 33.4 (C(CN)(CH_3)***C***H_2CH_2), 31.4 (S***C***H_2CH_3), 29.5 (C(CN)(CH_3)CH_2***C***H_2), 24.8 (C(CN)(***C***H_3)CH_2CH_2), 12.8 (SCH_2***C***H_3). (**Figure S1**)

Synthesis of 2-cyano-5-hydroxypentan-2-yl ethyl carbonotrithioate (CHPET). To a flame-dried three-neck round bottom flask, 4-cyano-4-(((ethylthio)carbonothioyl)thio)pentanoic acid (CEPA) (14.1 g, 53.6 mmol, 1 equiv) was added followed by the addition of dry tetrahydrofuran (500 mL) with the resulting solution cooled to -78 ˚C (mixture of dry ice and acetone). Borane tetrahydrofuran complex solution (1 M, 56.3 mL, 56.3 mmol, 1 equiv) was subsequently added in a dropwise fashion over 30 min. Reaction mixture was left to stir for 1 h, after which the cooling bath was removed and the reaction stirred overnight at ambient temperature under a N_2 atmosphere. After 18 h of stirring, methanol (100 mL) was added in five portions and stirred for 10 min. after each addition or until no further bubbling was

observed. After removal of the volatile solvents in vacuo, the organic residue was dissolved in diethyl ether (250 mL) and washed with saturated $NaHCO_3$ solution (3 × 250 mL) and then with brine (250 mL). Further extraction using diethyl ether from the collected aqueous layers was carried out. Combined organic layers were then dried over $MgSO_4$, filtered and evaporated to dryness. Purification was carried out using silica gel column chromatography (petroleum ether 40/60: ethyl acetate = 1:1) affording 2-cyano-5-hydroxypentan-2-yl ethyl carbonotrithioate (CHPET) as an orange red oil (9.7 g, 39.1 mmol, 73%). Then, the CTA CHPET was dried by static vacuum with P_2O_5 in the desiccator for one week to get rid of water. ^{1}H NMR (400 MHz, $CDCl_3$): δ (ppm): 3.73 (t, 2H, C***H₂***OH), 3.34 (q, 2H, SC***H₂***CH₃), 2.00-2.35 (m, 2H, C(CN)(CH₃)C***H₂***CH₂), 1.90 (s, 3H, C(CN)(C***H₃***)CH₂CH₂), 1.86 (m, 2H, C(CN)(CH₃)CH₂C***H₂***), 1.36 (t, 3H, SCH₂C***H₃***). ^{13}C NMR (100 MHz, $CDCl_3$): δ (ppm): 217.4 (***C***=S), 119.6 (C(***C***N)(CH₃)CH₂CH₂), 61.7 (***C***H₂OH), 47.0 (***C***(CN)(CH₃)CH₂CH₂), 35.8 (C(CN)(CH₃)***C***H₂CH₂), 31.3 (S***C***H₂CH₃), 27.9 (C(CN)(CH₃)CH₂***C***H₂), 24.9 (C(CN)(***C***H₃)CH₂CH₂), 12.8 (SCH₂***C***H₃). (**Figure S2**)

Synthesis of Poly(*ε*-Caprolactone) (PCL_{50} and PCL_{45}). In a nitrogen-filled glove box with oxygen and water content lower than 0.1 ppm, solutions of diphenylphosphate (DPP, 17 mg, 0.068 mmol, 1 equiv) in dry toluene (2.5 mL) and dual-head CTA (17 mg, 0.068 mmol, 1 equiv) in dry toluene (1 mL) were added to *ε*-caprolactone (543.3 mg, 4.76 mmol, 70 equiv) dissolved in dry toluene (1.5 mL). After stirring for 6 or 7 hours at room temperature, the solution was removed from the glove box, precipitated three times into cold diethyl ether dropwise and collected by centrifugation and dried in vacuum. Then analysed by NMR and SEC. ^{1}H NMR (400 MHz, $CDCl_3$): δ (ppm): 4.10 (t, 2H, C***H***₂OCO), 4.04 (t, 100H, CH₂C***H***₂O), 3.62 (m, 2H, C(CN)C***H***₂CH₂), 3.32 (q, 2H, SC***H***₂CH₃), 2.28 (100H, OCOC***H***₂CH₂), 1.86 (s, 3H, C(CN)(C***H***₃)CH₂), 1.72-1.54 (200H, OCOCH₂C***H***₂CH₂C***H***₂), 1.45-1.28 (100H, OCOCH₂CH₂C***H***₂CH₂). SEC (Chloroform, PMMA standard): M_n = 15.2 or 12.9 kg mol^{-1}, $Đ_M$ = 1.05. (**Figure S3, S5, S14, S19**)

Synthesis of PCL_{50}-*b*-$PDMA_{198}$. PCL_{50} (100 mg, 0.0168 mmol, 1eq), DMA (399.7 mg, 4.0320 mmol, 240 equiv) and AIBN (0.276 mg, 0.00168 mmol, 0.1 equiv) were dissolved in 1,4-dioxane (1 mL) and placed in an ampoule. The solution was then freeze-pump-thawed three times and heated for 2 h at 70 ˚C. The reaction was quenched by immersion of the ampoule in liquid nitrogen and the polymer was precipitated in ice-cold diethyl ether three times before being dried under vacuum and analysed. SEC (Chloroform, PMMA standard): M_n = 31.8 kg mol^{-1}, $Đ_M$ = 1.09. (**Figure S4, S5**)

Synthesis of PCL-ACM. Aminochloromaleimide (ACM), synthesized according to a previous method, was coupled to the PCL polymer backbone by esterification. In a typical coupling reaction, PCL (100 mg, 0.0168 mmol, 1 equiv), ACM (14.55 mg, 0.0504 mmol, 3 equiv), 4-dimethylaminopyridine (DMAP) (2.05 mg, 0.0168 mmol, 1 equiv) and N,N′-dicyclohexylcarbodiimide (DCC) (34.66 mg, 0.168 mmol, 10 equiv) were mixed together in an ampoule with 2 mL DCM. The solution was left stirring at room temperature for 2 days. The solution was filtered and the filtrate precipitated in diethyl ether three times and the resultant polymer was dried in vacuo. (**Figure S11, S12**)

Synthesis of PCL-*b*-PDMA-ACM. Aminochloromaleimide (ACM), synthesized according to a previous method,[6] was coupled to the PCL-*b*-PDMA polymer backbone by esterification. In a typical coupling reaction, PCL-*b*-PDMA (200 mg, 0.0078mmol, 1 equiv), ACM (6.64 mg, 0.023 mmol, 3 equiv), 4-dimethylaminopyridine (DMAP) (0.95 mg, 0.0078 mmol, 1 equiv) and N,N′-dicyclohexylcarbodiimide (DCC) (16.09 mg, 0.078 mmol, 10 equiv) were mixed together in an ampoule with 2 mL DCM. The solution was left stirring at room temperature for 2 days. The solution was filtered and the filtrate precipitated in diethyl ether three times and the resultant polymer was dried in vacuo. (**Figure S11, S13**)

Synthesis of PCL_{45}-*b*-$PNAM_{100}$. PCL_{45} (100 mg, 0.0186 mmol, 1eq), NAM (630.2 mg, 4.4640 mmol, 240 equiv) and AIBN (0.305 mg, 0.00186 mmol, 0.1 equiv) were dissolved in 1,4-dioxane (1 mL) and placed in an ampoule. The solution was then freeze-pump-thawed three times and heated for 4 h at 70 ˚C. The reaction was quenched by immersion of the ampoule in liquid nitrogen and the polymer was precipitated in ice-cold diethyl ether three times before being dried under vacuum and analysed. (**Figure S15, S19**)

Synthesis of PCL_{45}-*b*-$P4VP_{220}$. PCL_{45} (100 mg, 0.0186 mmol, 1eq), 4VP (586.7 mg, 5.5800 mmol, 300 equiv) and AIBN (0.305 mg, 0.00186 mmol, 0.1 equiv) were dissolved in DMF (1 mL) and placed in an ampoule. The solution was then freeze-pump-thawed three times and heated for 24 h at 70 ˚C. The reaction was quenched by immersion of the ampoule in liquid nitrogen and the polymer was precipitated in ice-cold diethyl ether three times before being dried under vacuum and analysed. (**Figure S16, S19**)

Synthesis of PCL_{45}-*b*-$P2VP_{134}$. PCL_{45} (100 mg, 0.0186 mmol, 1equiv), 2VP (391.1 mg, 3.7200 mmol, 200 equiv) and AIBN (0.305 mg, 0.00186 mmol, 0.1 equiv) were dissolved in 1,4-dioxane (1 mL) and placed in an ampoule. The solution was then freeze-pump-thawed three times and heated for 24 h at 70 ˚C. The reaction was quenched by immersion of the ampoule

in liquid nitrogen and the polymer was precipitated in ice-cold diethyl ether three times before being dried under vacuum and analysed. (**Figure S17, S19**)

Synthesis of PCL_{45}-*b*-$PDMAEMA_{160}$. PCL_{45} (100 mg, 0.0186 mmol, 1equiv), DMAEMA (877.2 mg, 5.5800 mmol, 300 equiv) and AIBN (0.305 mg, 0.00186 mmol, 0.1 equiv) were dissolved in 1,4-dioxane (1 mL) and placed in an ampoule. The solution was then freeze-pump-thawed three times and heated for 18 h at 70 ˚C. The reaction was quenched by immersion of the ampoule in liquid nitrogen and the polymer was precipitated in ice-cold diethyl ether three times before being dried under vacuum and analysed. (**Figure S18, S19**)

Quaternization of PCL_{45}-*b*-$PDMAEMA_{160}$. PCL-*b*-PDMAEMA were quaternized according to a previous report.[7] A clear solution of PCL-*b*-PDMAEMA (100 mg, 0.0033 mmol, 1equiv) in nitromethane (10 mL) was stirred for 10 min in a Schlenk flask under nitrogen flow. Then iodomethane (23.34 mg, 0.1644 mmol, 50 equiv), dissolved in 4 mL of nitromethane, was added dropwise under nitrogen. The solution became slightly yellow after iodomethane was completely added. The reaction was continued for 2 days under reflux. The mixture was then concentrated and dissolved in DMF, dialyzed against deionized water for 3 days to remove the organic solvent. Finally, the product was then freeze-dried for 1 day and vacuum-dried at 50 °C for 2 days. (**Figure S19**)

Crystallization-Driven Self-Assembly of PCL-*b*-PDMA Cylinder. Block copolymer PCL-*b*-PDMA (20 mg) was added to 4 mL of ethanol (5.0 mg mL^{-1}) in a vial. The samples were heated at 70 °C without stirring on a heating block for 3 h before cooling to room temperature. The sample was aging for 5 days to yield micron-long polydisperse cylinders.

Preparation of PCL-*b*-PDMA Seeds Solution. The crystalline cylinders were sonicated using a Bandelin Sonopuls sonication probe in an ice water bath for total 20 min. Polymer solution was fractured for 10 cycles of 2 min sonication each with interval of 20 min to yield short crystalline seeds. For determination of the average seeds' length at least 100 particles were analysed.

Living Epitaxial Growth of 2D Platelet. PCL homopolymer with block copolymer PCL-*b*-PDMA, or PCL-ACM homopolymer with block copolymers PCL-*b*-PDMA-ACM dissolved in THF, DMF or Chloroform (10 mg mL^{-1} or 50 mg mL^{-1}) was added to a dispersion of crystalline seeds (0.01 mg mL^{-1}) in a screw cap vial. (Typical living CDSA procedure: if fix the ratio of unimer-to-seed is 10, 10 uL of unimer solution (10 mg/mL, PCL/PCL-b-PX in a 1:1 w/w ratio dissolved in THF) was added to 1 mL of seed solution (0.01 mg/mL in ethanol))

After shaking the vials for 5 s, the solution was aged for 2 days at room temperature. The unimer-to-seed ratio was altered by adding different volumes of unimer solution to the dispersion of seed micelles (When the ratio of unimers to seeds above 20, the concentration of unimers changes to 50 mg mL^{-1}). The of contour lengths, widths and areas were measured from AFM and CLSM images.

Sequential Growth of Multi-Layered Platelet. PCL homopolymer block copolymers PCL-*b*-PDMA dissolved in THF, DMF or Chloroform (10 mg mL^{-1}) was added to a dispersion of as prepared platelets seeds and each time aged for 2 days before analysis by AFM and TEM images.

Growth of Fluorescent Multi-Layered Platelet. The blending unimers of PCL homopolymer block copolymers PCL-*b*-PDMA (or PCL-ACM and PCL-*b*-PDMA-ACM) dissolved in THF, DMF or Chloroform (10 mg mL^{-1}) was added alternatively to a dispersion of as prepared platelets seeds and aged for 2 days before analysis by AFM and CLSM images.

Synthesis of PCL-H. The removal of the trithiocarbonate end group was performed following procedure by Beth and Thomas et al. PCL, 1-ethylpiperidine hypophosphite (EPHP), tributylphosphine (PBu_3) and AIBN (molar mass ratio = 1:10:2:0.33 equiv) were dissolved in DMF and degassed by three freeze-pump-thaw cycles in an ampoule. The mixture solution was stirred for 3 h at 100 °C, over the course of which the solution became colourless from yellow. After precipitating into cold diethyl ether three times and being dried under vacuum, the inactive RAFT group (-H) terminated polymers as a white powder, were obtained and analyzed. SEC (Chloroform, PMMA standard): M_n = 14.8 kg mol^{-1}, $Đ_M$ = 1.12. (**Figure S38, S39**)

Synthesis of PCL-*b*-PDMA-H. The removal of the trithiocarbonate end group was performed following procedure by Beth and Thomas et al. PCL-*b*-PDMA, 1-ethylpiperidine hypophosphite (EPHP), tributylphosphine (PBu_3) and AIBN (molar mass ratio = 1:10:2:0.33 equiv) were dissolved in DMF and degassed by three freeze-pump-thaw cycles in an ampoule. The mixture solution was stirred for 3 h at 100 °C, over the course of which the solution became colourless from yellow. After precipitating into cold diethyl ether three times and being dried under vacuum, inactive RAFT group (-H) terminated polymers as a white powder, were obtained and analyzed. SEC (Chloroform, PMMA standard): M_n = 35.6 kg mol^{-1}, $Đ_M$ = 1.18. (**Figure S38, S40**)

Epitaxial growth of platelets. PCL homopolymer with block copolymers PCL-*b*-PDMA, or PCL-H homopolymer with block copolymers PCL-*b*-PDMA-H dissolved in THF, DMF or

Chloroform (10 mg mL^{-1}) was added to a dispersion of crystalline seeds (0.01 mg mL^{-1}) (Typical living CDSA procedure: if fix the ratio of unimer-to-seed is 10, 10 uL of unimer solution (10 mg/mL, PCL/PCL-b-PX in a 1:1 w/w ratio dissolved in THF) was added to 1 mL of seed solution (0.01 mg/mL in ethanol)) and then aged for 2 days before analysis by AFM.

Sequential growth of multi-layered platelets. The blend of PCL homopolymer and block copolymer PCL-*b*-PDMA (or PCL-H and PCL-*b*-PDMA-H) dissolved in THF, DMF or Chloroform (10 mg mL^{-1}) was added to a dispersion of as prepared platelets seeds (0.01 mg mL^{-1}) and aged for 2 days before analysis by AFM.

Light induced polymerization (photoiniferter polymerization and PET-RAFT polymerization).

Photoiniferter polymerization on platelets surface. The DMA or fluorescent dye monomer (various equivalent for weight mass, from 0 – 400 equiv) was added into as-prepared PCL/PCL-*b*-PDMA platelets solution (solvent: ethanol without extra treatment for platelets solution) and degassed under a nitrogen flow with a rubber plug for 30 min in ice bath. Then the solution was placed in the UV crosslinker and irradiated with 405 nm light under 25 °C for 4 h. The solution was then dialysed in ethanol to remove unreacted DMA or fluorescent dye monomer (ABM methacrylate based), for following characterization.

PET-RAFT polymerization on platelets surface. The DMA or fluorescent dye monomer and photocatalyst (Eosin Y, 0.01 equiv) (equivalent for weight mass) was added into as-prepared PCL/PCL-*b*-PDMA platelets solution (solvent: ethanol without extra treatment for platelets solution) and degassed under a nitrogen flow with a rubber plug for 30 min in ice bath. Then the solution was placed in the self-made light set-up (shown in Figure SX) and irradiated with green light under 25 °C for 4 h. The solution was then dialysed in ethanol to remove unreacted DMA or fluorescent dye monomer (ABM methacrylate based) and photocatalyst (Eosin Y, 0.01 equiv wt%).

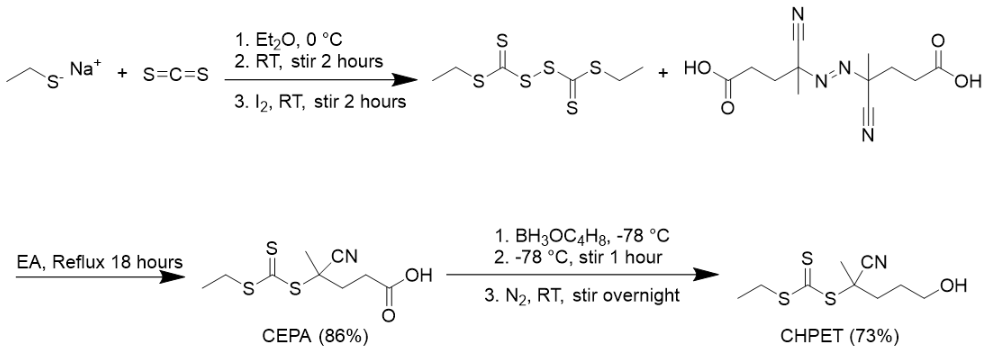

Scheme S1. Synthesis of dual-head RAFT chain transfer agent CHPET.

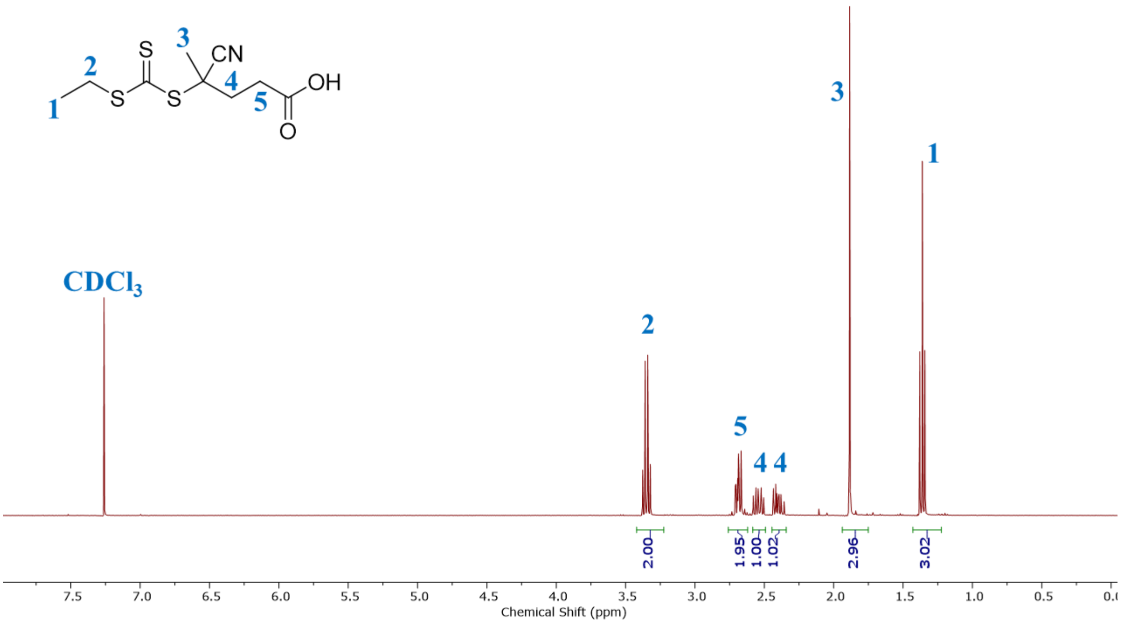

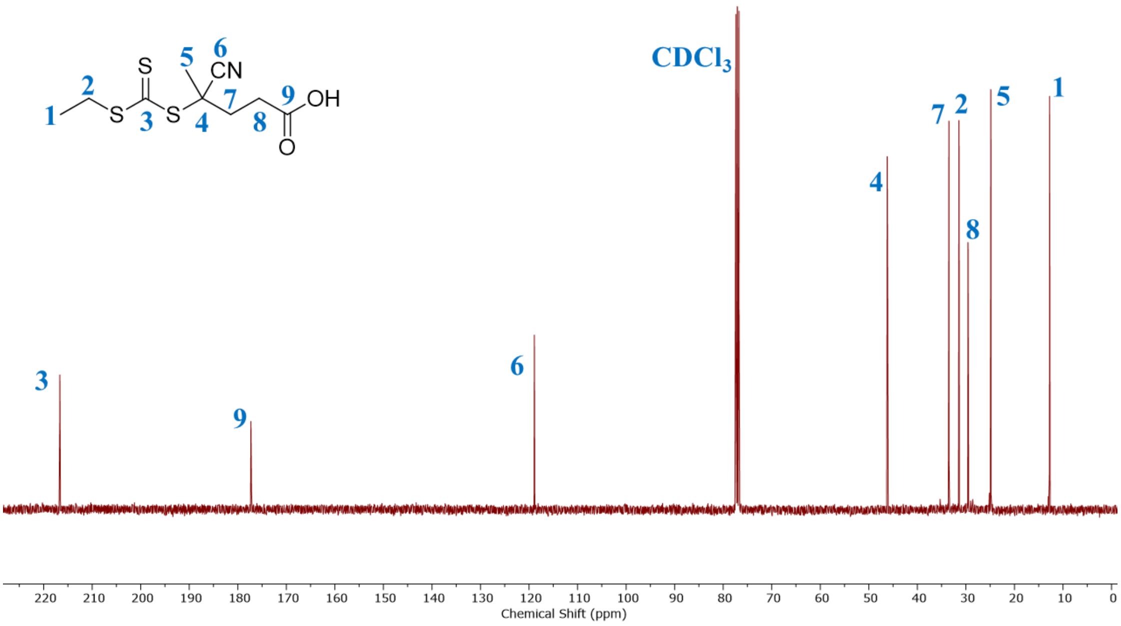

Figure S1. ^{1}H NMR (400 MHz) and ^{13}C NMR (100 MHz) spectra of 4-cyano-4-(((ethylthio)carbonothioyl)thio)pentanoic acid (CEPA) in $CDCl_3$.

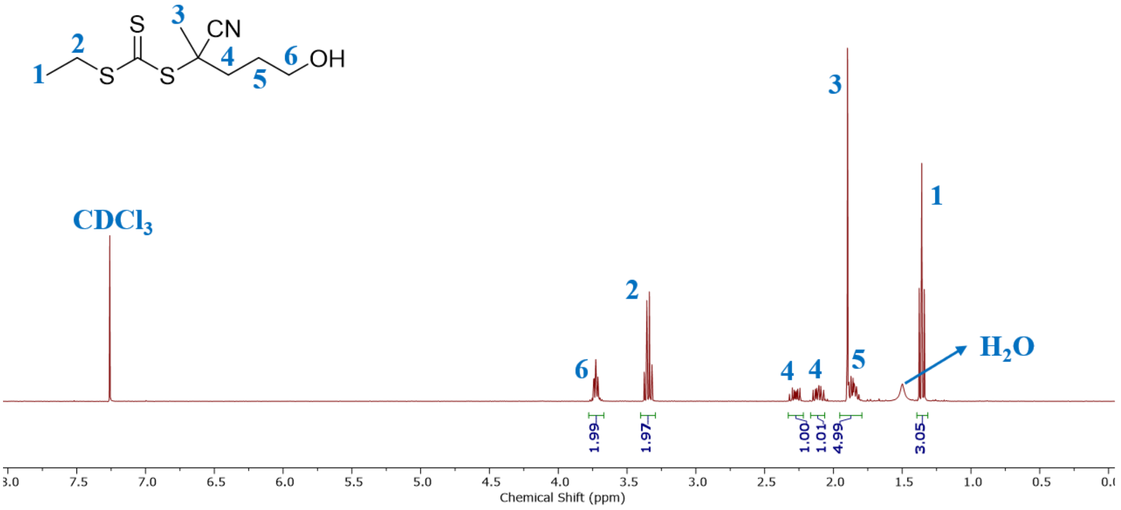

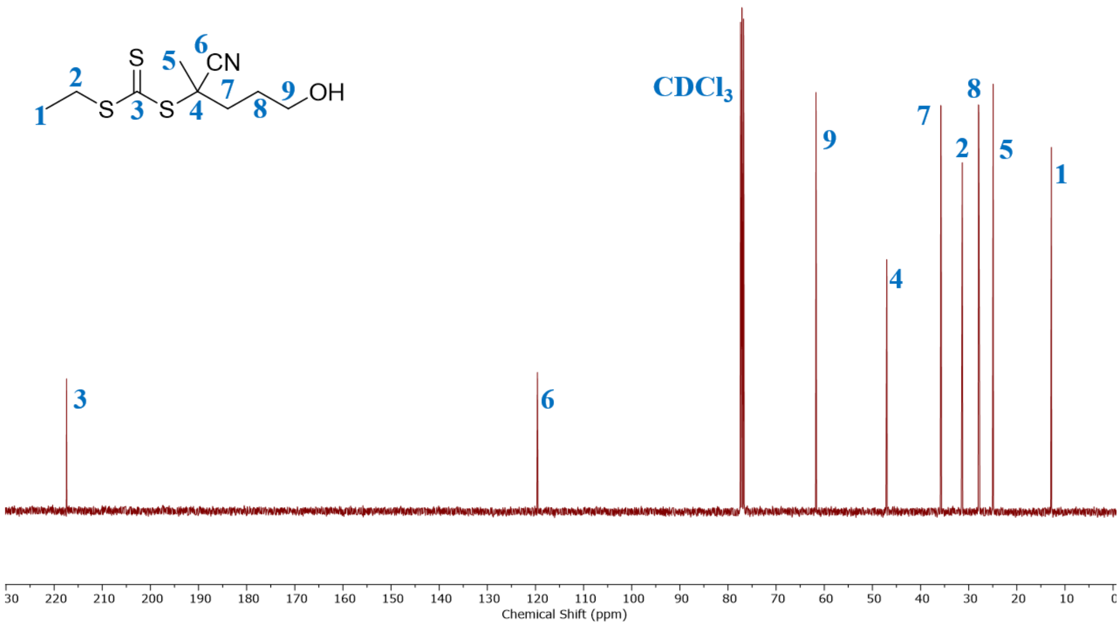

Figure S2. ^{1}H NMR (400 MHz) and ^{13}C NMR (100 MHz) spectra of 2-cyano-5-hydroxypentan-2-yl ethyl carbonotrithioate (CHPET) in $CDCl_3$.

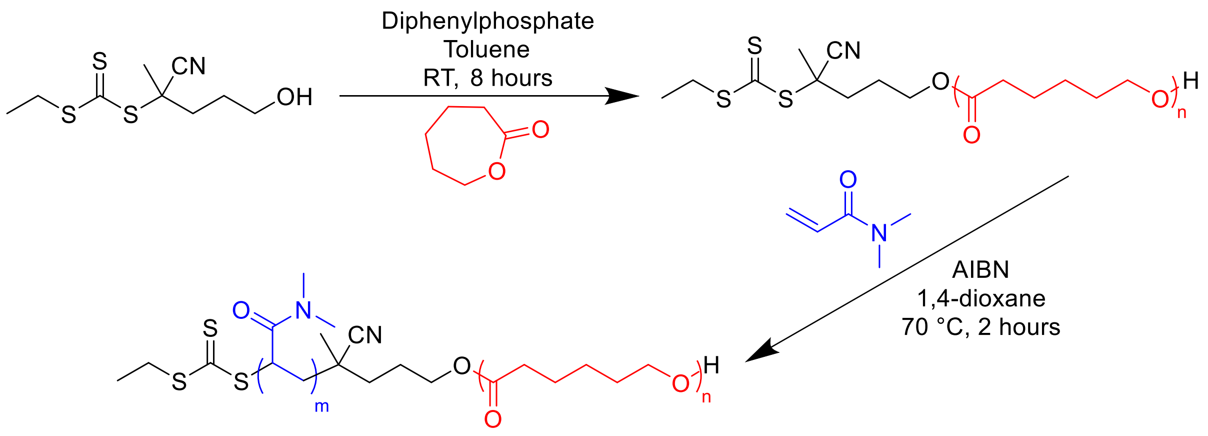

Scheme S2. Synthesis of PCL macro CTA and PCL-*b*-PDMA block copolymer by ROP and RAFT polymerization.

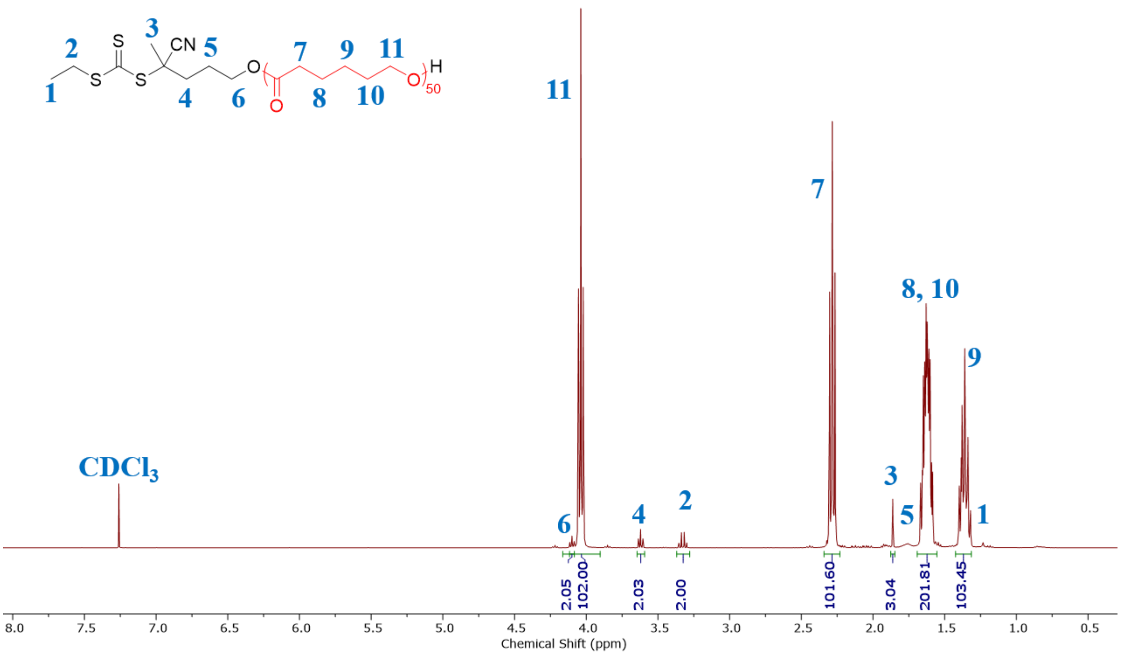

Figure S3. ^{1}H NMR spectrum of PCL_{50} in $CDCl_3$ (400 MHz).

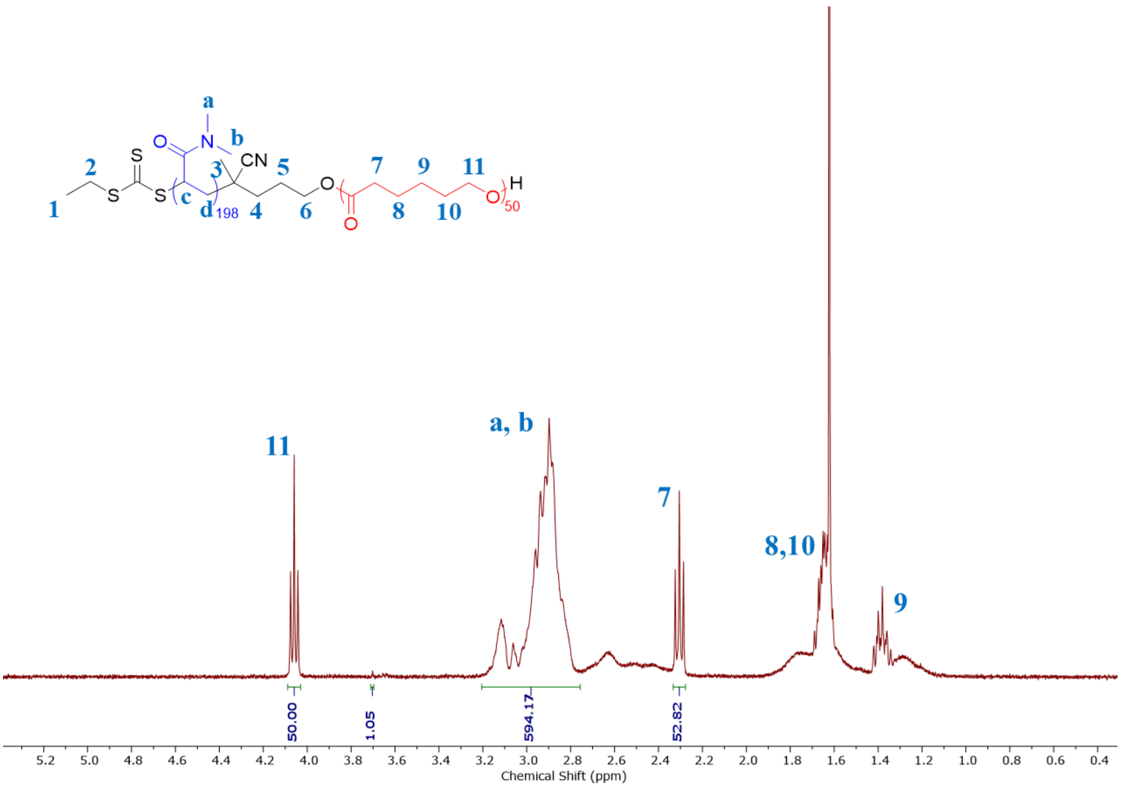

Figure S4. ^{1}H NMR spectrum of PCL_{50}-*b*-$PDMA_{198}$ in $CDCl_3$ (400 MHz).

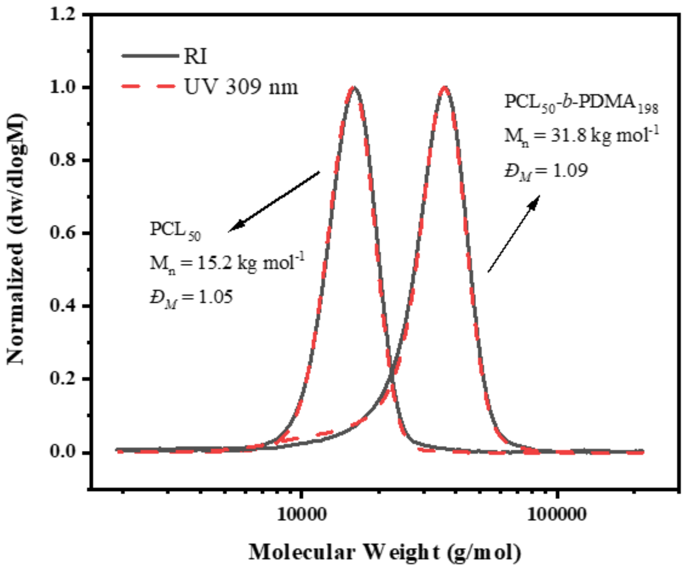

Figure S5. SEC chromatogram (RI and UV 309 nm trace) of PCL_{50}-*b*-$PDMA_{198}$ using $CHCl_3$ with 0.5% TEA as an eluent (PMMA standard).

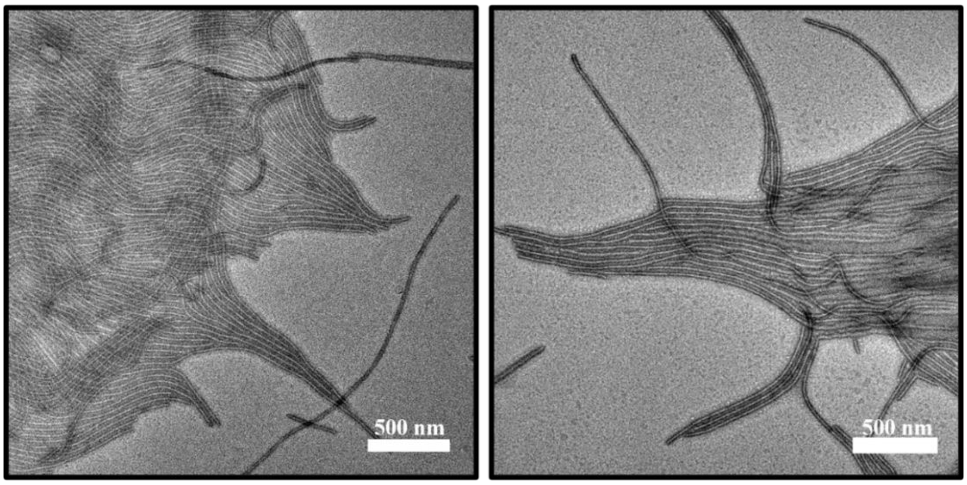

Figure S6. Transmission electron microscopy (TEM) images of cylindrical micelles (0.5 mg mL^{-1}) prepared using PCL_{50}-*b*-$PDMA_{198}$ in ethanol, heating at 70 °C for 3 h and subsequently cooling down to room temperature (stained with 1 wt. % uranyl acetate in water).

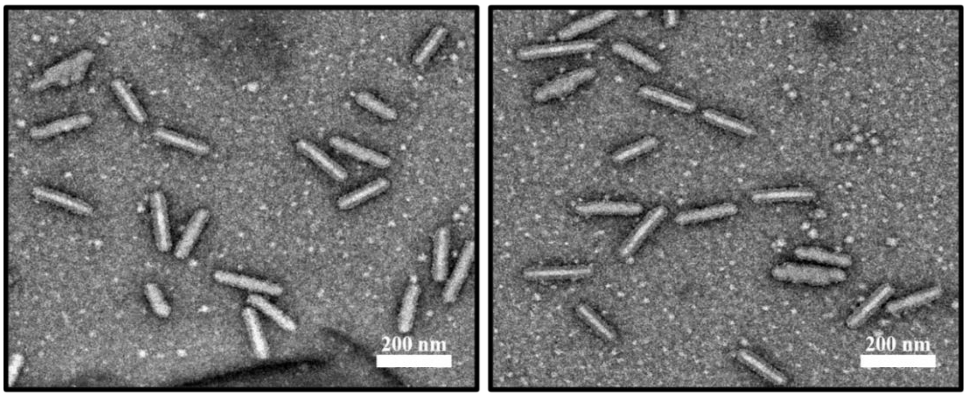

Figure S7. TEM micrographs of PCL_{50}-*b*-$PDMA_{198}$ cylinders assembled in ethanol after sonication at 0 °C for 20 min using a sonic probe (stained with 1 wt. % uranyl acetate in water). After sonication, short and uniform 1D seeds were achieved (L_n = 125.3 nm, L_w = 127.3 nm, L_w/L_n= 1.02).

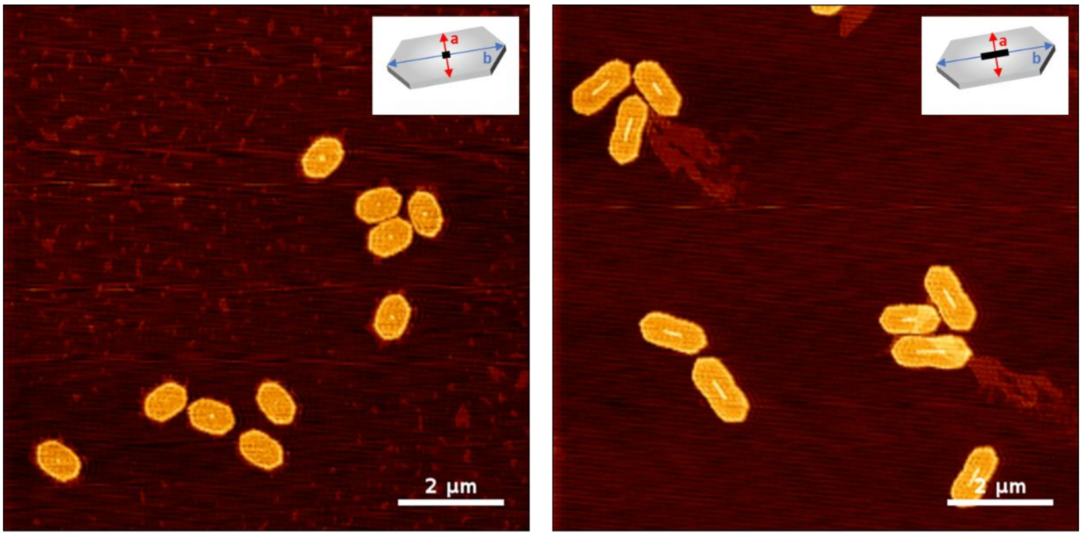

Short seeds for living CDSA

Long seeds for living CDSA

Figure S8. AFM images of PCL-based platelets using short and long seeds for living CDSA.

Regarding the placement of seeds, due to the nature of epitaxial growth, the seeds are positioned in the middle of the platelets. This has been confirmed through AFM characterizations. As for orientation, observing the orientation within the platelet structure can be challenging due to the small size of normal seeds. To address this challenge, we prepared elongated seeds for seeded growth, which led to the formation of platelets through living CDSA. Upon characterization of the AFM image, it became evident that the seeds are oriented along and parallel to the long axis (b-axis) of the 2D platelets. Qiu et al. demonstrated a similar CDSA result in PFS system, which the seed rods located on the centre of platelets and parallel to the long axis.[8]

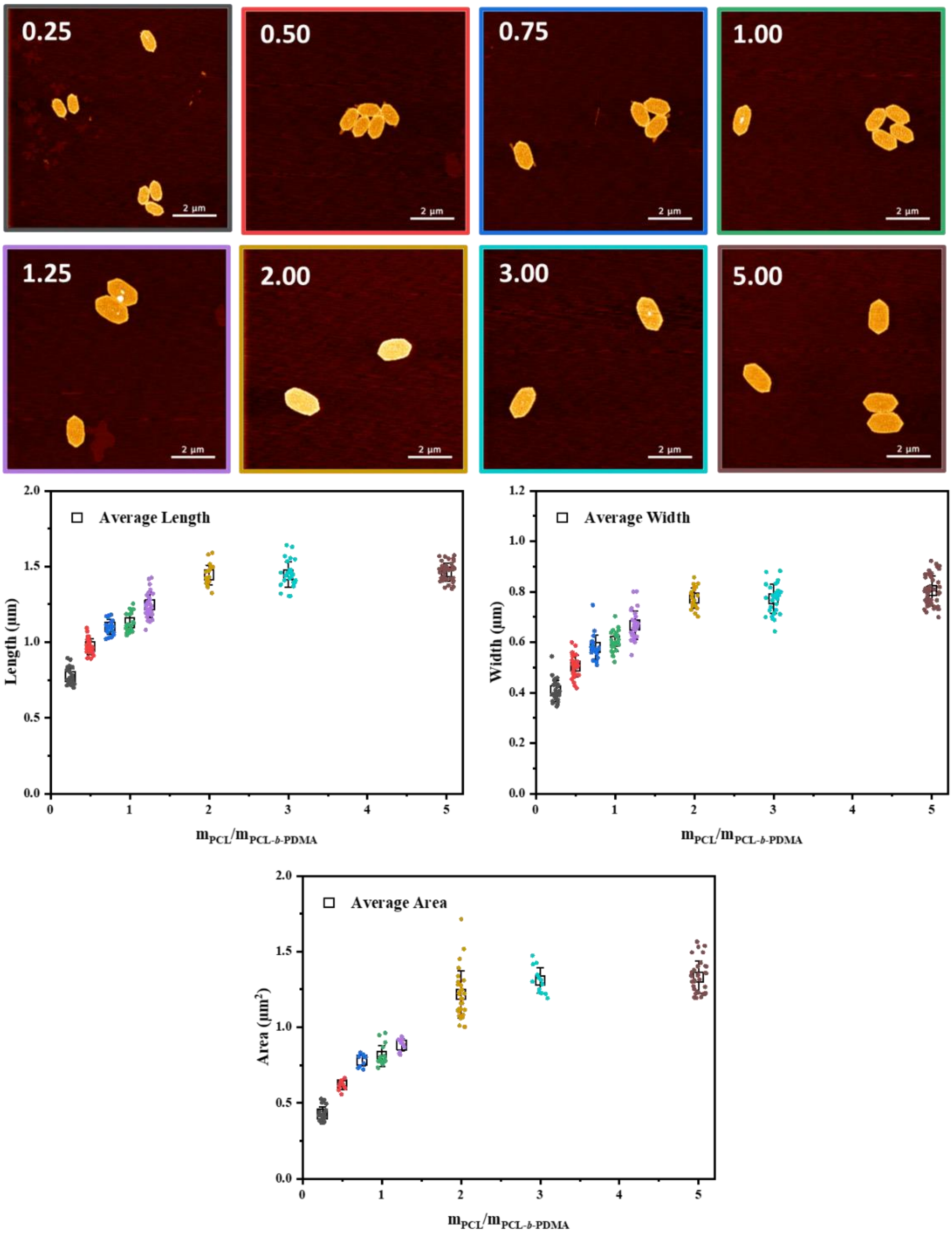

Figure S9. Effect of PCL homopolymer concentration on the assemblies by seeded epitaxial growth. m_{PCL}: $m_{PCL\text{-}b\text{-}PDMA}$=0.25, 0.5, 0.75, 1, 1.25, 2, 3 and 5; The crystalline seeds are prepared from PCL_{50}-*b*-$PDMA_{198}$ cylinder. The unimer-to-seed ratio is fixed as 10. Top: AFM images of PCL_{50}/PCL_{50}-*b*-$PDMA_{198}$ blending platelets; Bottom: Plots of 2D assemblies' length, width, and area versus m_{unimer}/m_{seed}.

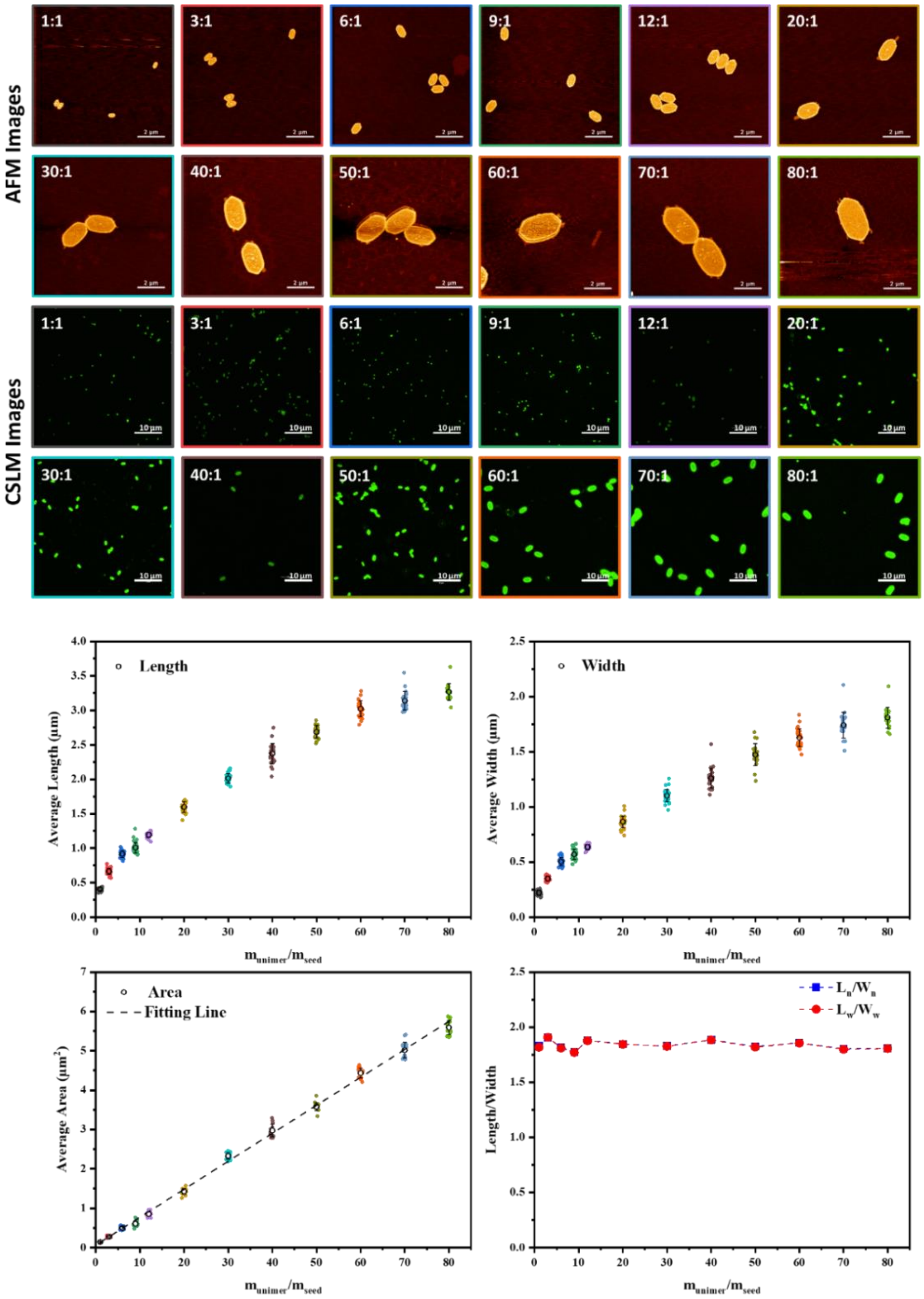

Figure S10. Exquisite control of 2D platelets growth. AFM images and CLSM images of PCL_{50}/PCL_{50}-*b*-$PDMA_{198}$ (mass ratio=1) blending platelets grown from PCL_{50}-*b*-$PDMA_{198}$ crystalline seeds with munimer/mseed of 1, 3, 6, 9, 12, 20, 30, 40, 50, 60, 70 and 80; graph showing the linear dependence of 2D platelet length, width, area and aspect ratio (length/width) upon the unimer-to-seed ratio. (Normally, the concentration of unimers is 10 mg mL^{-1}, when the ratio of unimers to seeds above 20, it changes to 50 mg mL^{-1}, avoiding to introduce too much good solvent.) With the increase in the unimers-to-seed ratio, the growth rate of length and width decreases.

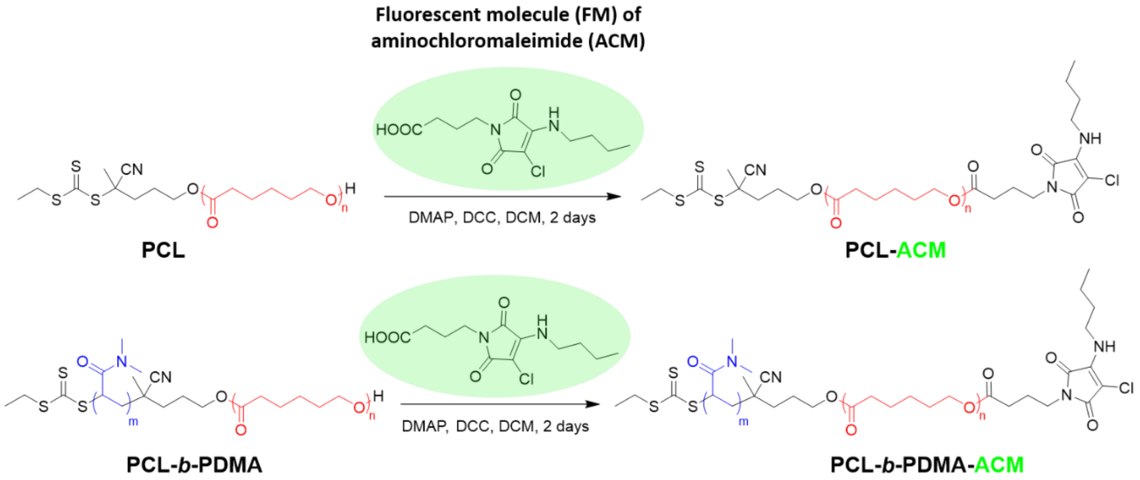

Scheme S3. Design of fluorescent functionalized homopolymer and block copolymer.

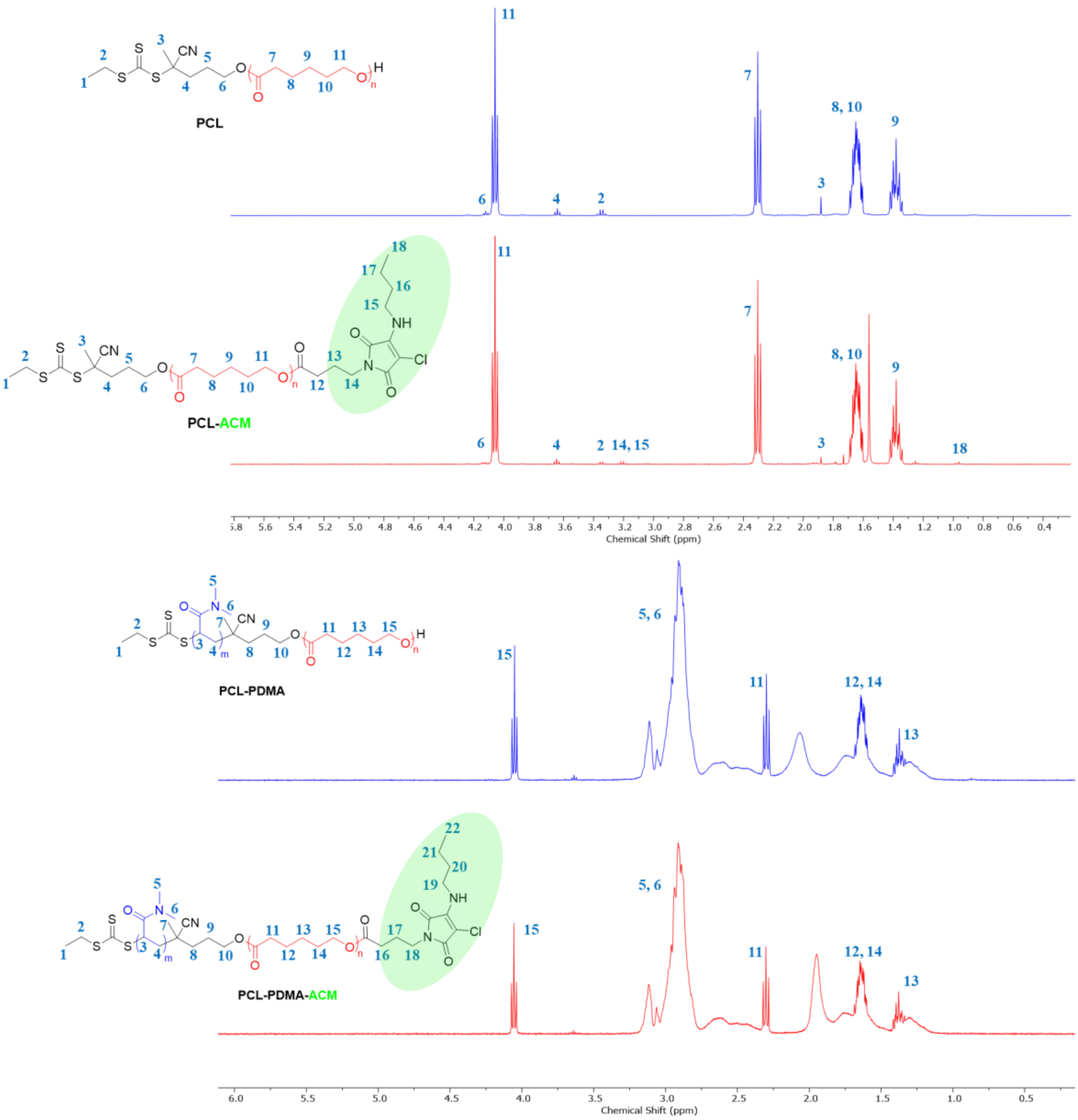

Figure S11. ^{1}H NMR spectra of PCL_{50} homopolymer and block copolymer PCL_{50}-b-$PDMA_{198}$ before and after coupling with fluorescent molecule ACM in $CDCl_3$ (400 MHz).

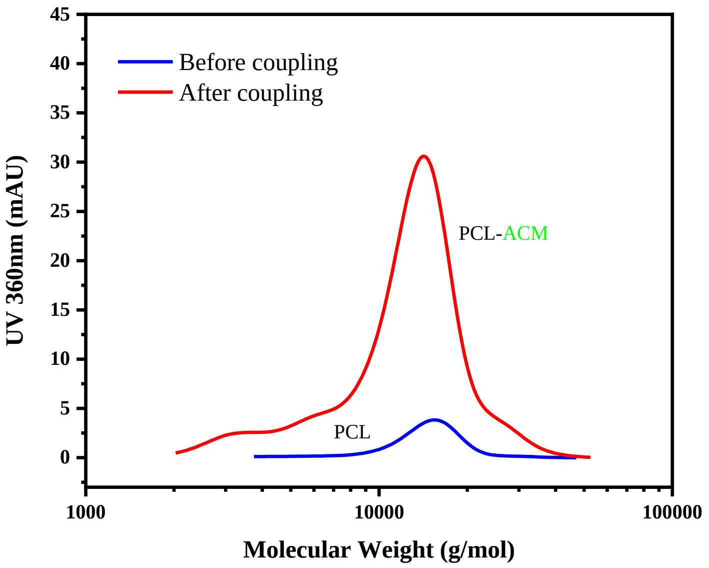

Figure S12. SEC chromatogram (UV trace, 360 nm) of PCL_{50} homopolymer before and after coupling with fluorescent molecule ACM using $CHCl_3$ with 0.5% TEA as an eluent with PMMA standards.

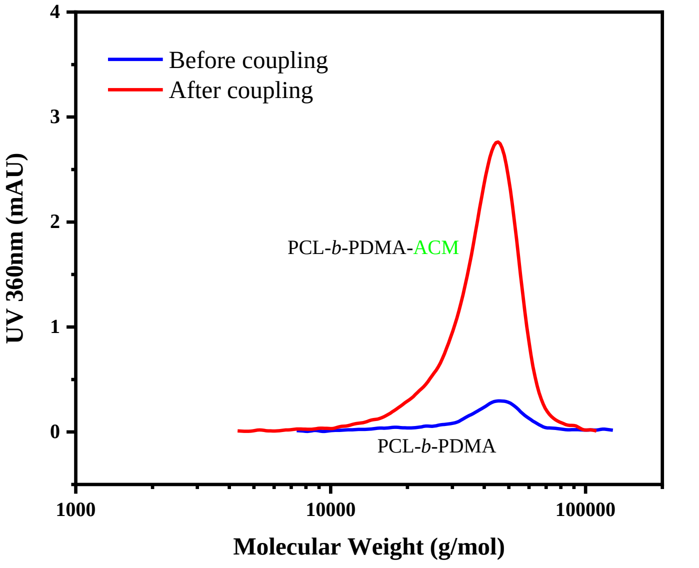

Figure S13. SEC chromatogram (UV trace, 360 nm) of block copolymer PCL_{50}-*b*-$PDMA_{198}$ before and after coupling with fluorescent molecule ACM using CHCl3 with 0.5% TEA as an eluent with PMMA standards.

Table S1. Exquisite control of 2D platelets growth. Length, width and area dispersity of platelet.[a]

$m_{unimers}/m_{seeds}$	Length			Width			Area			Length/Width	
	L_n (nm) [b]	L_w (nm) [c]	L_w/L_n [d]	W_n (nm) [e]	W_w (nm) [f]	W_w/W_n [g]	A_n (10^4 nm^2) [h]	A_w (10^4 nm^2) [i]	A_w/A_n [j]	L_n/W_n [k]	L_w/W_w [l]
**1**	403.9	405.2	1.00	220.6	223.0	1.02	14.2	14.4	1.02	1.83	1.82
**3**	663.4	666.5	1.00	348.2	349.5	1.00	27.3	27.4	1.01	1.91	1.91
**6**	914.7	917.4	1.00	504.3	506.9	1.01	49.6	49.9	1.01	1.81	1.81
**9**	1008.0	1013.5	1.01	568.9	572.5	1.01	60.6	61.2	1.01	1.77	1.77
**12**	1192.0	1193.1	1.00	634.8	635.7	1.00	85.3	85.7	1.00	1.88	1.88
**20**	1597.7	1601.3	1.00	865.1	868.4	1.00	142.4	142.8	1.00	1.85	1.84
**30**	2012.7	2015.0	1.00	1101.1	1103.8	1.00	232.7	233.0	1.00	1.83	1.83
**40**	2374.1	2383.0	1.00	1259.4	1265.8	1.01	297.2	298.1	1.00	1.89	1.88
**50**	2687.1	2690.2	1.00	1472.9	1479.4	1.00	358.1	358.4	1.00	1.82	1.82
**60**	3023.0	3027.5	1.00	1627.8	1631.9	1.00	443.3	443.7	1.00	1.86	1.86
**70**	3139.5	3145.4	1.00	1741.0	1748.7	1.00	501.4	502.1	1.00	1.80	1.80
**80**	3269.3	3273.7	1.00	1808.7	1813.8	1.00	559.1	559.6	1.00	1.81	1.80

[a]. The length, width and area of the platelet was collected by AFM; [b, e, h]. The length, width and area of number average micelle, respectively; [c, f, i]. The length, width and area of weight average micelle, respectively; [d, g, j]. The distribution of micelle length, width and area, respectively; [k, l]. The length-to-width ratio of number and weight average micelle, respectively.

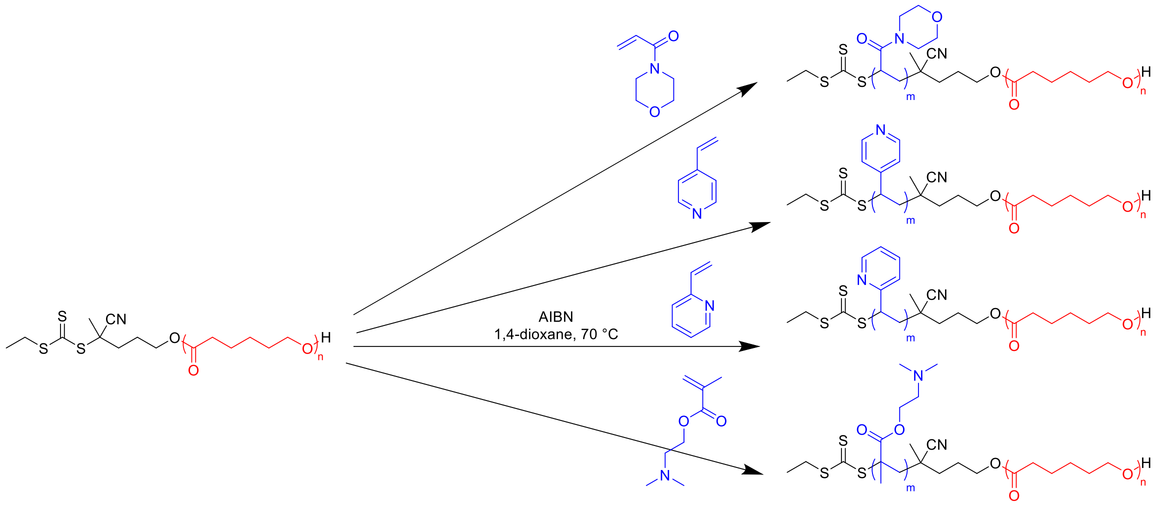

Scheme S4. Synthesis route of PCL-*b*-PNAM, PCL-*b*-P4VP, PCL-*b*-P2VP, PCL-*b*-PDMAEMA block copolymer by RAFT polymerization.

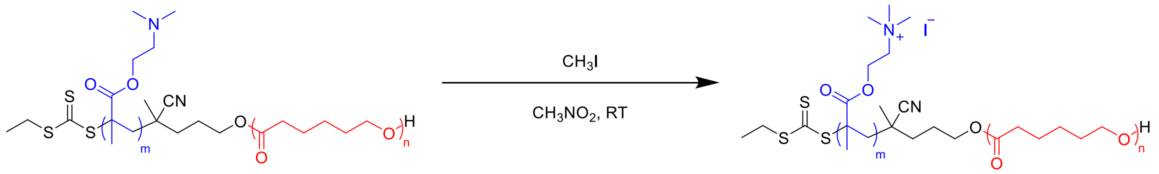

Scheme S5. Quaternization of PCL-*b*-PDMAEMA.

In the process of forming 2D platelet systems via living CDSA, the preferred shape tends to be hexagonal-like platelets, primarily due to the influence of homopolymer PCL, which reflects the intrinsic morphology of PCL crystals. Block copolymer PCL-b-PX (where X can be DMA, NAM, 4VP, 2VP, DMAEMA, *q*DMAEMA-I) serves as a stabilizer to enhance colloidal stability in the solvent. This is essential because PCL alone is hydrophobic and tends to aggregate/precipitate from the solvent.

Therefore, in selecting the corona block's degree of polymerization (DP), two main factors were considered: (1) ensuring that the corona does not disrupt the assembly's morphology while maintaining the 2D nanostructure, and (2) ensuring that the corona provides sufficient colloidal stability to keep the platelets uniformly suspended in the solvent, achieved through interactions between the corona and solvent. Taking into account the above considerations and drawing from previous reports on PCL-b-PDMAEMA[9] and PLLA-b-PDMA[10] systems, we have selected a core-to-corona ratio window ranging from 1:2 to 1:5. This range fulfills the aforementioned criteria. Consequently, we have synthesized various block copolymers, including PCL_{45}-*b*-$PNAM_{100}$, PCL_{45}-*b*-$P4VP_{220}$, PCL_{45}-*b*-$P2VP_{134}$, PCL_{45}-*b*-$PDMAEMA_{160}$, and PCL_{45}-*b*-*q*$PDMAEMA_{160}$-I.

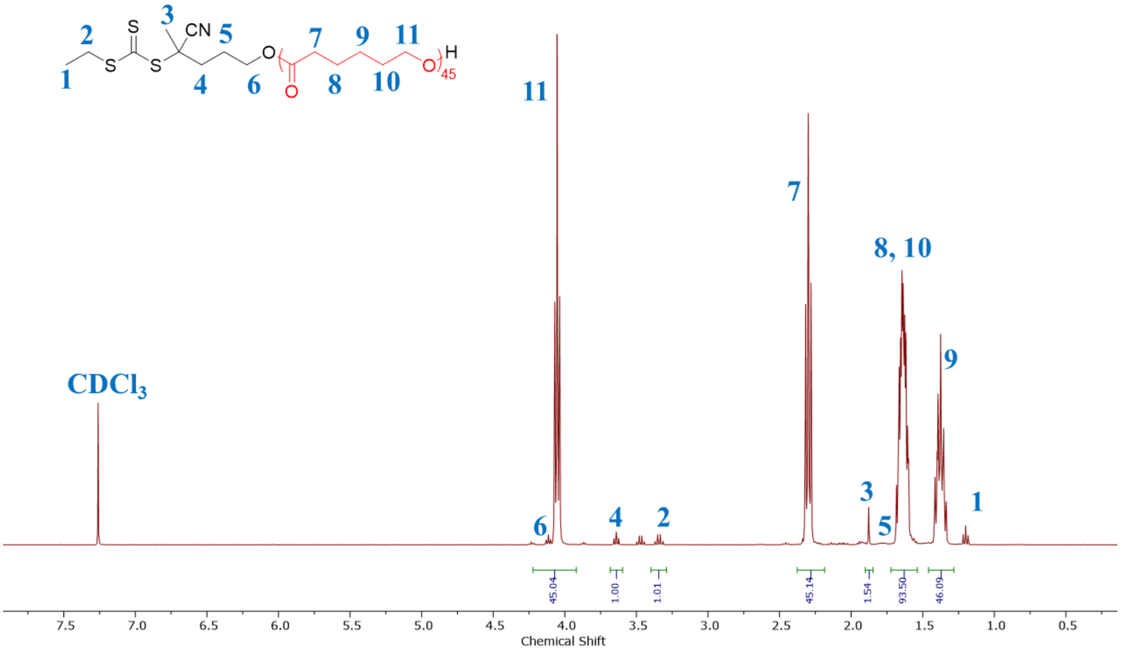

Figure S14. ^{1}H NMR spectrum of PCL_{45} in $CDCl_3$ (400 MHz).

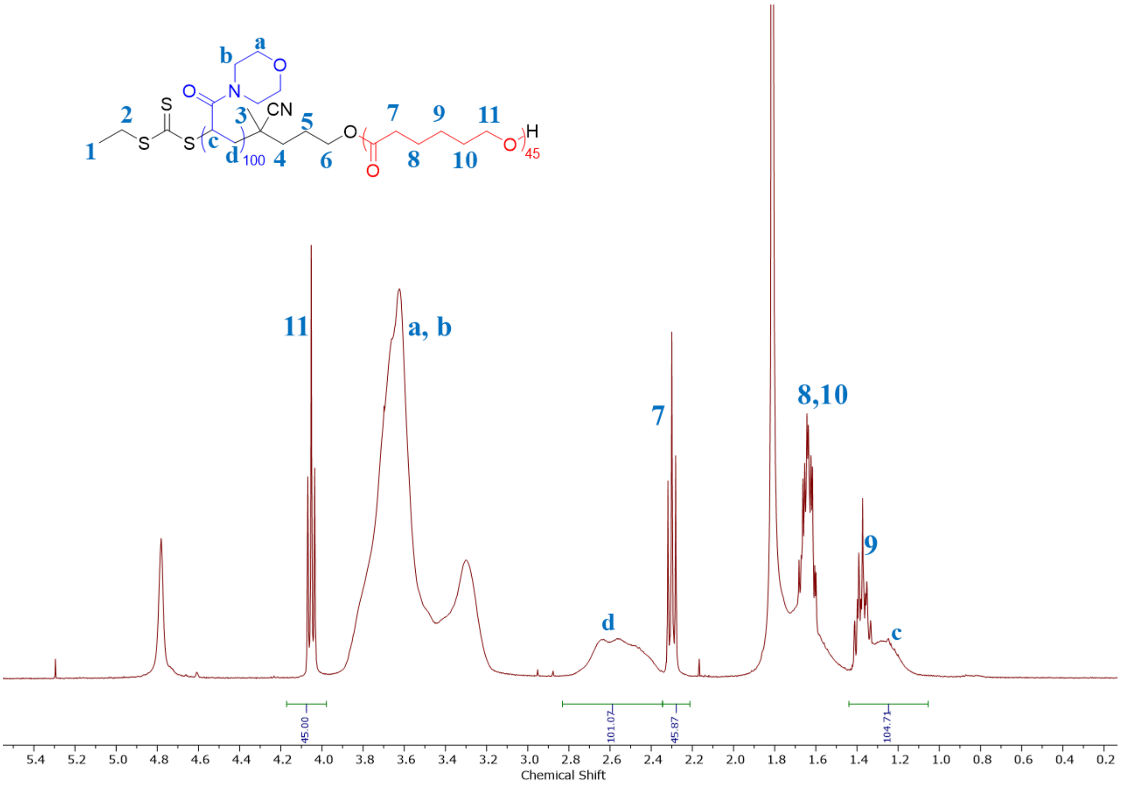

Figure S15. ^{1}H NMR spectrum of PCL_{45}-*b*-$PNAM_{100}$ in $CDCl_3$ (400 MHz).

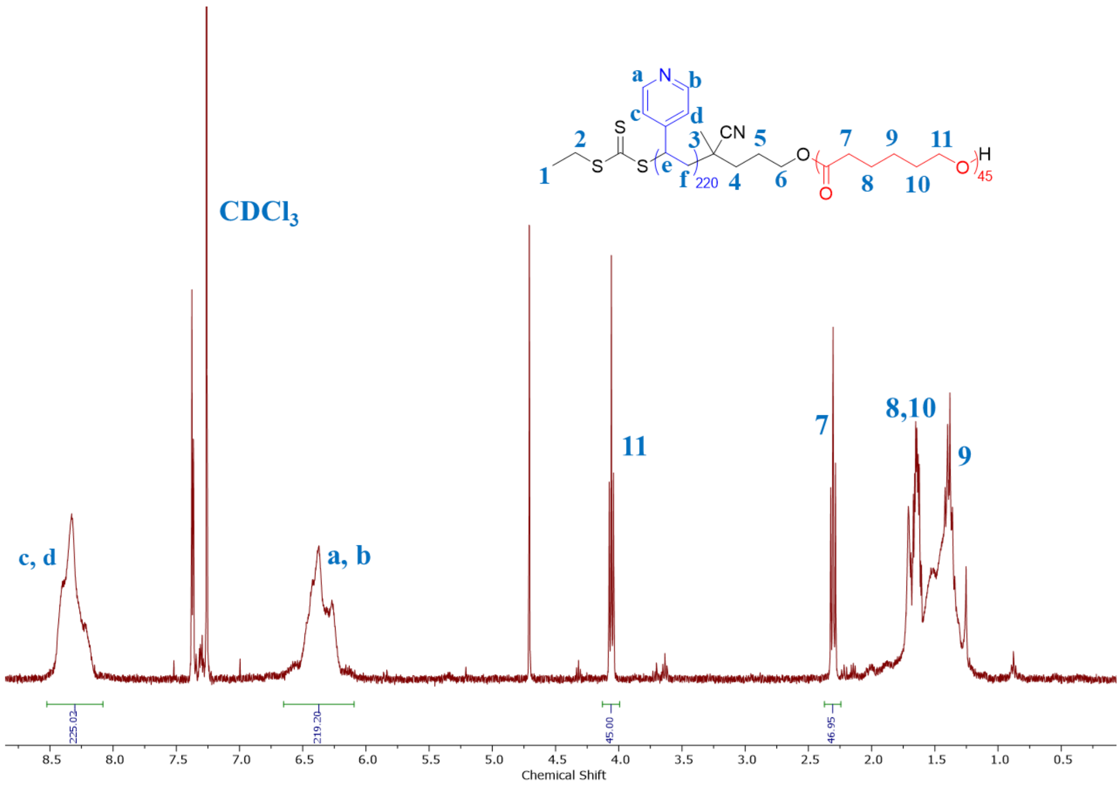

Figure S16. ^{1}H NMR spectrum of PCL_{45}-*b*-$P4VP_{220}$ in $CDCl_3$ (400 MHz).

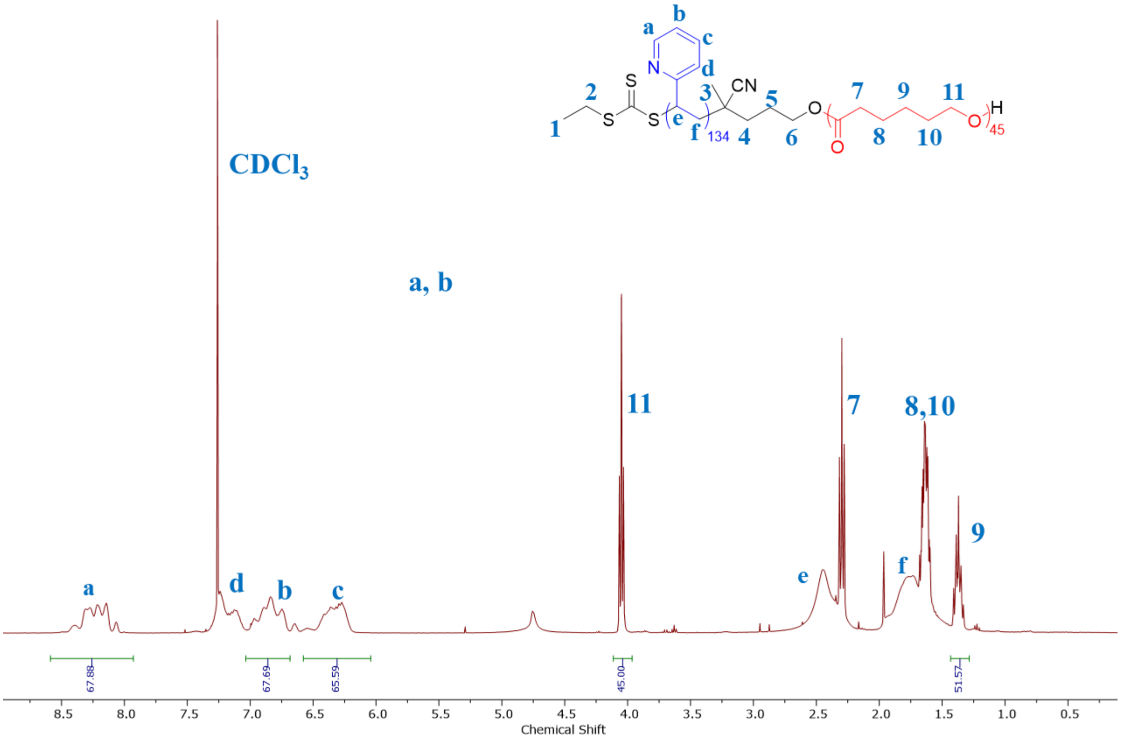

Figure S17. ^{1}H NMR spectrum of PCL_{45}-*b*-$P2VP_{134}$ in $CDCl_3$ (400 MHz).

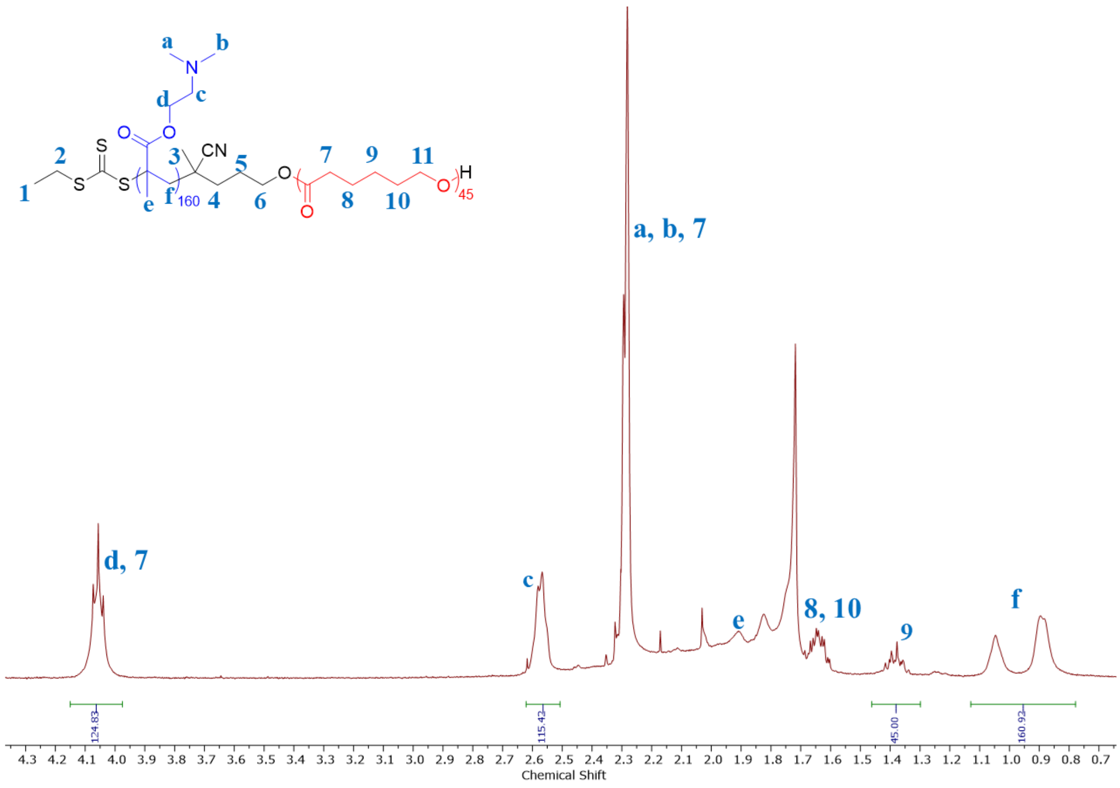

Figure S18. ^{1}H NMR spectrum of PCL_{45}-*b*-$PDMAEMA_{160}$ in $CDCl_3$ (400 MHz).

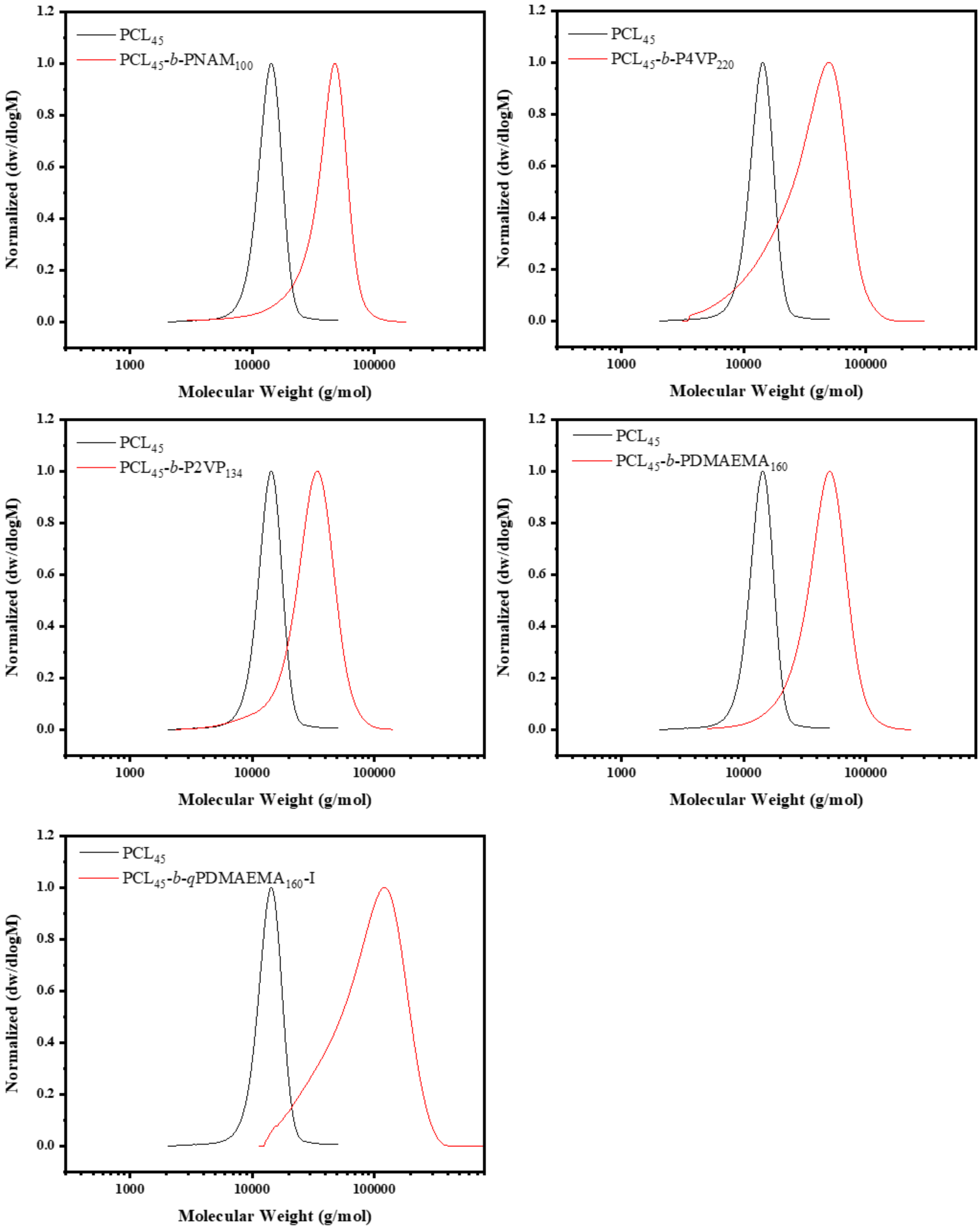

Figure S19. SEC chromatogram (RI trace) of PCL_{45}, PCL_{45}-*b*-$PNAM_{100}$, PCL_{45}-*b*-$P2VP_{134}$, PCL_{45}-*b*-$P4VP_{220}$, PCL_{45}-*b*-$PDMAEMA_{160}$, PCL_{45}-*b*-*q*$PDMAEMA_{160}$-I using $CHCl_3$ with 0.5% TEA or DMF with 5 mM NH_4BF_4 as an eluent.

Table S2. Molecular characteristics of the polymers used in this study.

Polymer	$M_{n,\ NMR}$[a] (kg/mol)	$M_{n,\ SEC}$[b](kg/mol)	$Đ_{M,\ SEC}$[b]
PCL_{45}	5.3	12.9	1.05
PCL_{45}-*b*-$PNAM_{100}$	19.5	34.3	1.27
PCL_{45}-*b*-$P4VP_{220}$	28.4	26.8	1.56
PCL_{45}-*b*-$P2VP_{134}$	19.4	26.0	1.29
PCL_{45}-*b*-$PDMAEMA_{160}$	30.4	40.4	1.23
PCL_{45}-*b*-*q*$PDMAEMA_{160}$-I	53.2	105.6	1.32

a) The polymer compositions were determined from ^{1}H NMR sprectra;

b) The polydispersity was obtained from SEC analysis with $CHCl_3$ or DMF as eluent.

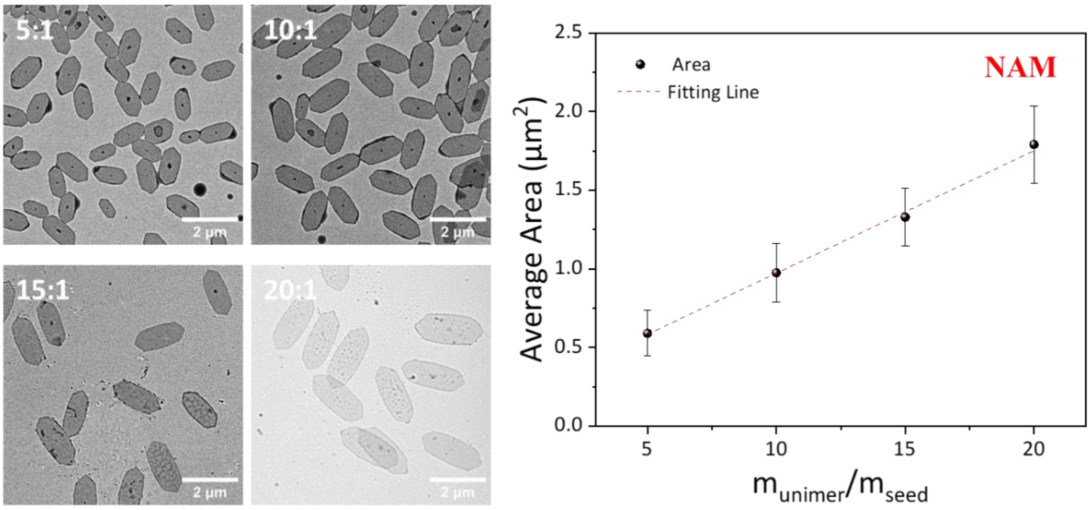

Figure S20. Living CDSA of PCL_{45}/PCL_{45}-*b*-$PNAM_{100}$ platelets. (Left) AFM images of 2D platelets of PCL_{45}/PCL_{45}-*b*-$PNAM_{100}$ (1:1, w/w, 10 mg mL^{-1} in $CHCl_3$) grown from 1D crystalline seeds of PCL_{50}-*b*-$PDMA_{198}$ (0.01 mg mL-1, 1 mL) with m_{unimer} to m_{seed} (unimer-to-seed) ratios of 5, 10, 15 and 20. (Right) Plots of area of 2D platelet against unimer-to-seed ratios.

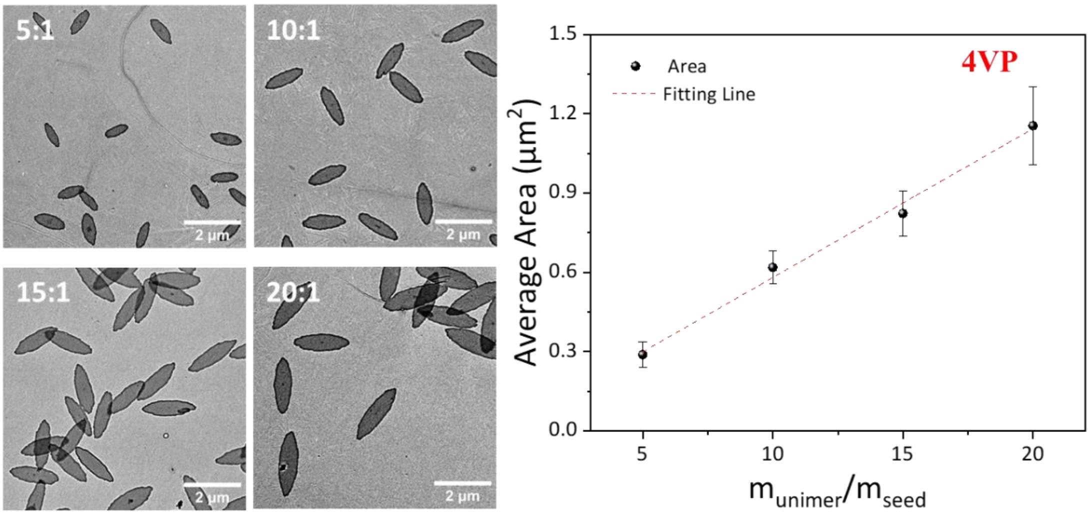

Figure S21. Living CDSA of PCL_{45}/PCL_{45}-*b*-$P4VP_{220}$ platelets. (Left) AFM images of 2D platelets of PCL_{45}/PCL_{45}-*b*-$P4VP_{220}$ (1:1, w/w, 10 mg mL^{-1} in $CHCl_3$) grown from 1D crystalline seeds of PCL_{50}-*b*-$PDMA_{198}$ (0.01 mg mL^{-1}, 1 mL) with m_{unimer} to m_{seed} (unimer-to-seed) ratios of 5, 10, 15 and 20. (Right) Plots of area of 2D platelet against unimer-to-seed ratios.

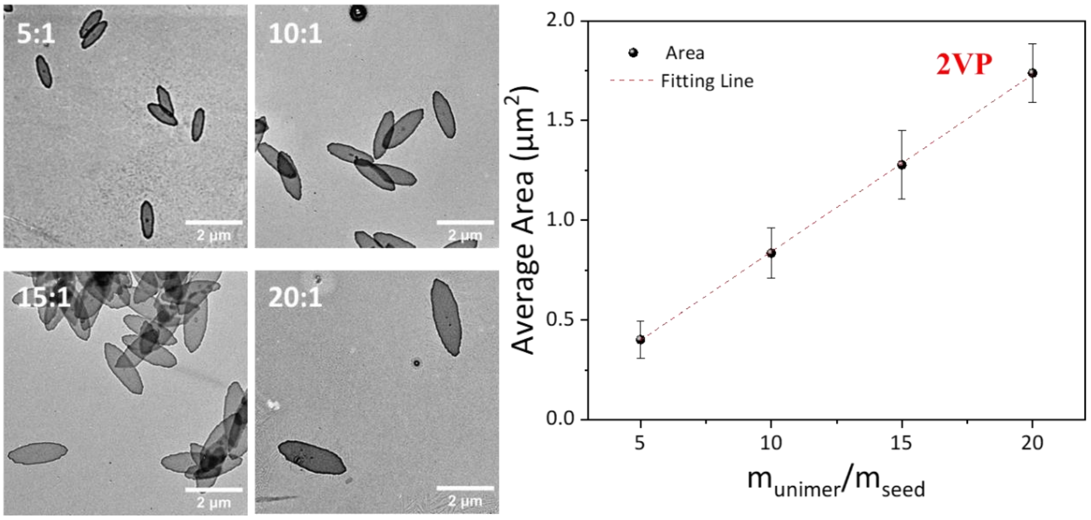

Figure S22. Living CDSA of PCL_{45}/PCL_{45}-*b*-$P2VP_{134}$ platelets. (Left) AFM images of 2D platelets of PCL_{45}/PCL_{45}-*b*-$P2VP_{134}$ (1:1, w/w, 10 mg mL^{-1} in $CHCl_3$) grown from 1D crystalline seeds of PCL_{50}-*b*-$PDMA_{198}$ (0.01 mg mL^{-1}, 1 mL) with m_{unimer} to m_{seed} (unimer-to-seed) ratios of 5, 10, 15 and 20. (Right) Plots of area of 2D platelet against unimer-to-seed ratios.

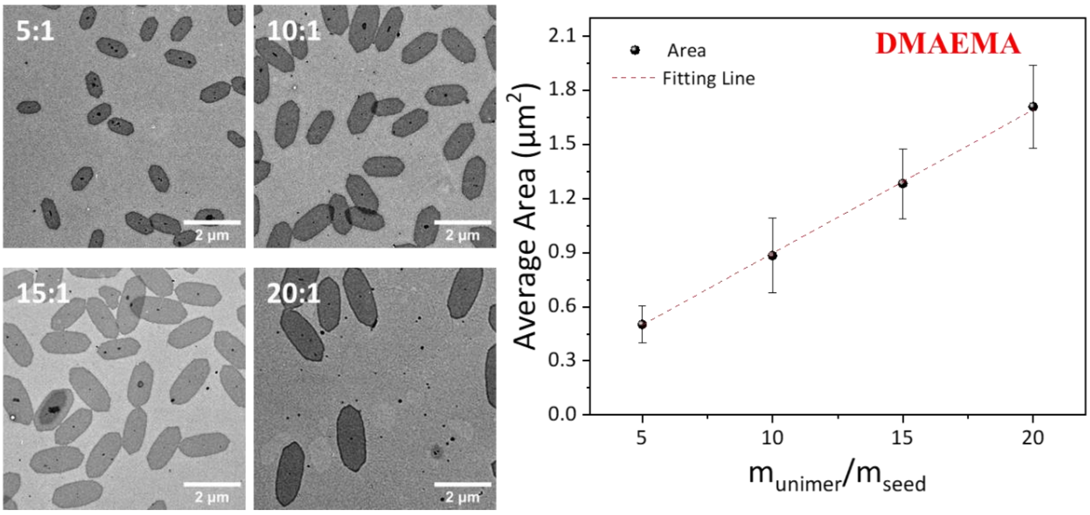

Figure S23. Living CDSA of PCL45/PCL45-*b*-PDMAEMA160 platelets. (Left) AFM images of 2D platelets of PCL45/PCL45-*b*-PDMAEMA160 (1:1, w/w, 10 mg mL-1 in $CHCl_3$) grown from 1D crystalline seeds of PCL50-*b*-PDMA198 (0.01 mg mL^{-1}, 1 mL) with m_{unimer} to m_{seed} (unimer-to-seed) ratios of 5, 10, 15 and 20. (Right) Plots of area of 2D platelet against unimer-to-seed ratios.

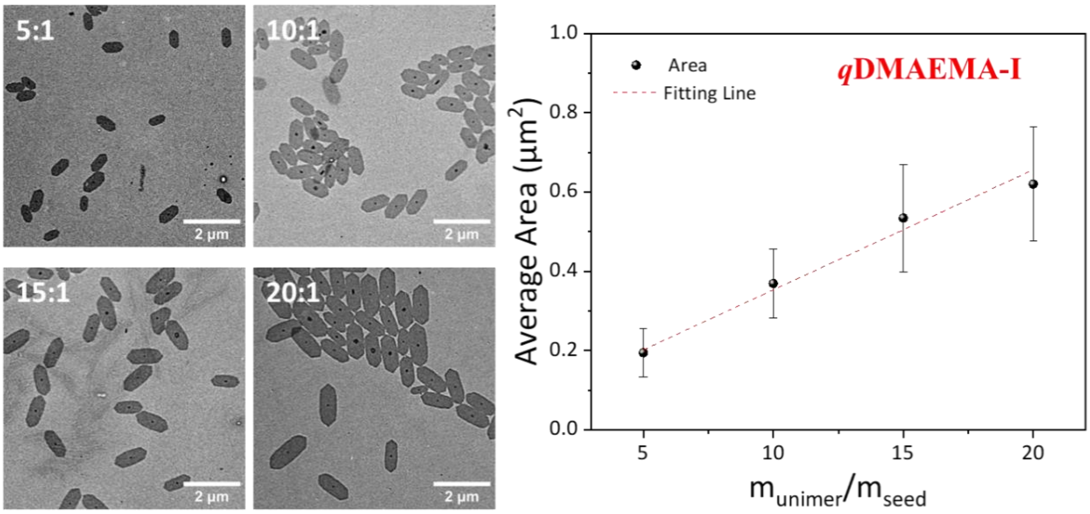

Figure S24. Living CDSA of PCL45/PCL45-*b*-*q*PDMAEMA160-I platelets. (Left) AFM images of 2D platelets of PCL45/PCL45-*b*-*q*PDMAEMA160-I (1:1, w/w, 5 mg mL^{-1} in DMF) grown from 1D crystalline seeds of PCL50-*b*-PDMA198 (0.01 mg mL^{-1}, 1 mL) with m_{unimer} to m_{seed} (unimer-to-seed) ratios of 5, 10, 15 and 20. (Right) Plots of area of 2D platelet against unimer-to-seed ratios.

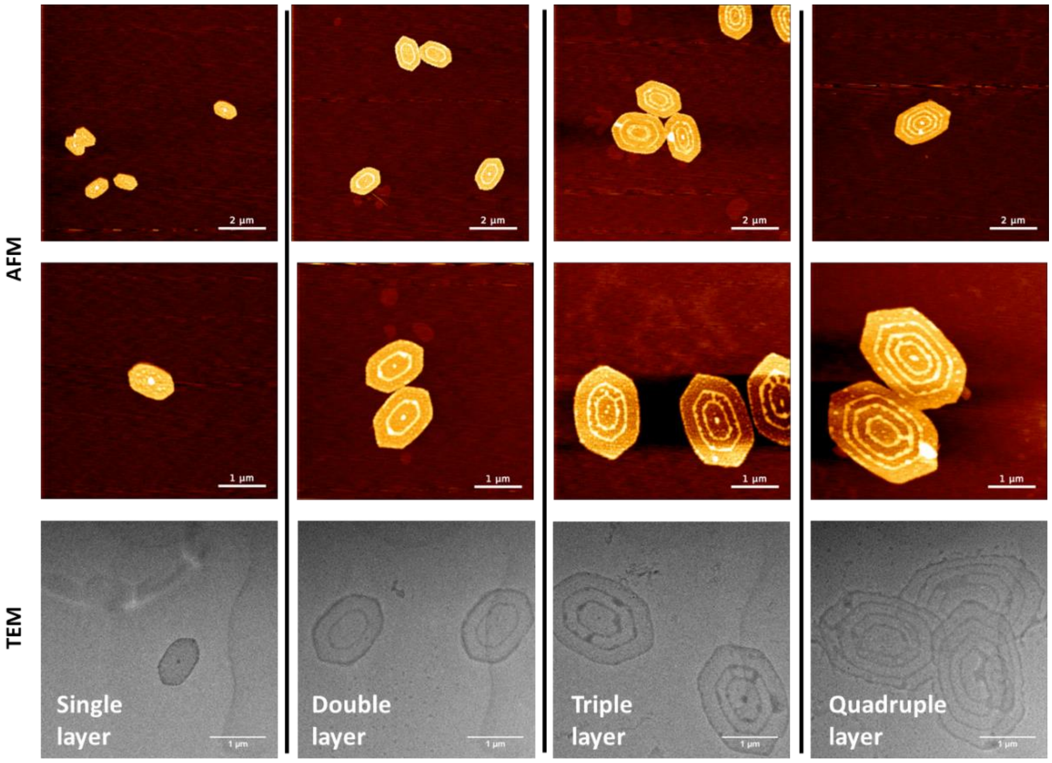

Figure S25. AFM and TEM images of multi-layered platelets of single, double, triple and quadruple layered after sequential seeded growth.

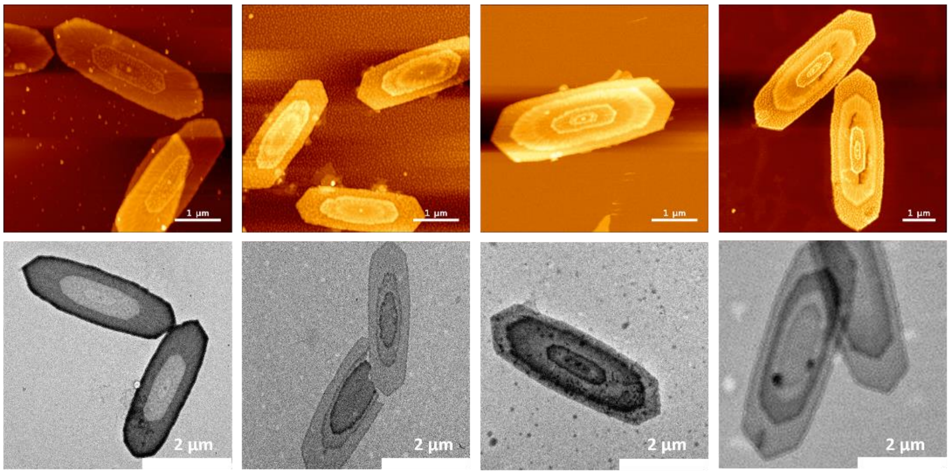

	Corona of BCPs	Corona of BCPs	Corona of BCPs	Corona of BCPs
Inner layer	*q*DMAEMA-I	DMAEMA	DMA	DMA
Middle layer	DMA	DMA	DMAEMA	DMAEMA
Outer layer	DMAEMA	*q*DMAEMA-I	*q*DMAEMA-I	DMA

Figure S26. The AFM and TEM images of heterocorona platelets of $L_{DMAEMA\text{-}I}$-L_{DMA}-L_{DMAEMA}, L_{DMAEMA}-L_{DMA}-$L_{qDMAEMA\text{-}I}$, L_{DMA}-L_{DMAEMA}-$L_{qDMAEMA\text{-}I}$, L_{DMA}-L_{DMAEMA}-L_{DMA} (L_{DMA} represents PCL/PCL-*b*-PDMA layer) via sequential seeded growth. (AFM images scale is 1 µm; TEM images scale is 2 µm.) TEM images were stained with 1 wt. % uranyl acetate in water.

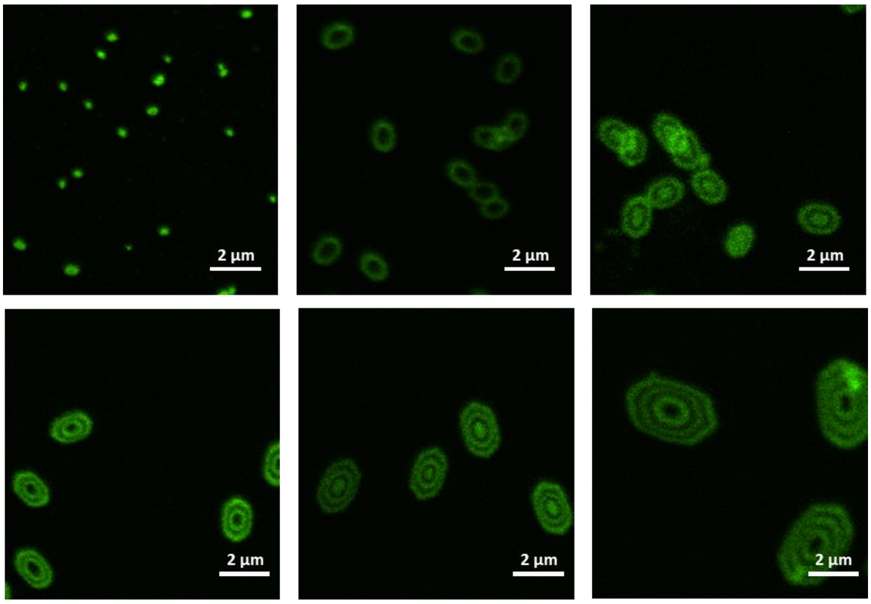

Figure S27. CLSM images of fluorescent modified multi-layered platelets by sequential epitaxial growth. (scale bar = 2 µm)

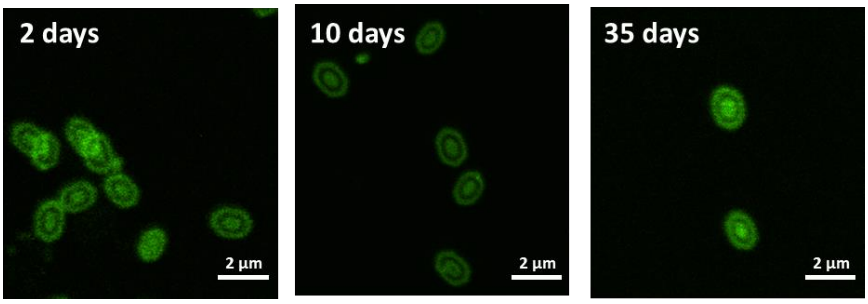

Figure S28. Stability of platelets. CLSM images of fluorescent platelets after different period of time (2, 10 and 35 days). (scale bar = 2 µm)

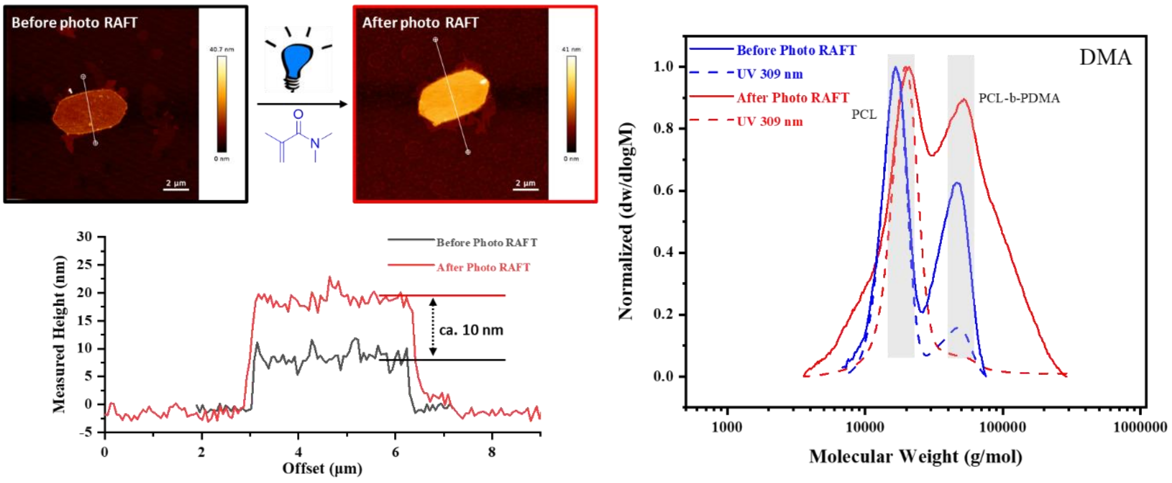

Figure S29. (Left) AFM images and height profiles of platelet before and after photo-iniferter polymerization (height measured at selected region); (Right) SEC chromatogram of PCL/PCL-*b*-PDMA platelets before and after photo-iniferter polymerization using $CHCl_3$ with 0.5% TEA as an eluent with PMMA standards. (Blue line present the molecular weight distribution of original platelets; Red line presents the molecular weight distribution of platelets after photo-iniferter polymerization.)

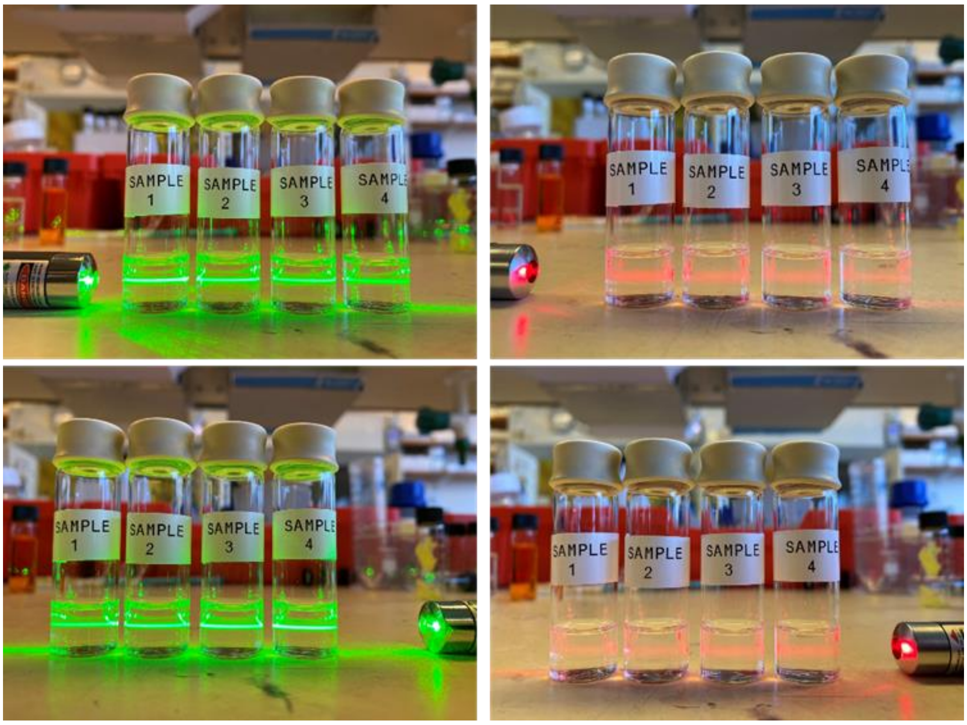

Figure S30. Images of Tyndall effect for platelet solution. (Sample 1) pure platelet solution; (Sample 2) platelet solution with added monomer; (Sample 3) platelet solution after 30 minutes of ice cooling; and (Sample 4) platelet solution with added monomer after 30 minutes of ice cooling.

Through observation of the Tyndall effect, no changes or the formation of large particles (precipitation) after cooling can be detected under green and red laser radiation from both side direction. Consequently, there is no precipitation effect when cooling the platelet + monomer solution under ice-cold conditions before light-induced polymerization. Recently, Tong group also demonstrated the living CDSA process of PCL/PCL-b-PDMA and PHL/PHL-b-PDMA system at low temperatures, which can form well defined 2D nanostructures without precipitation.[11]

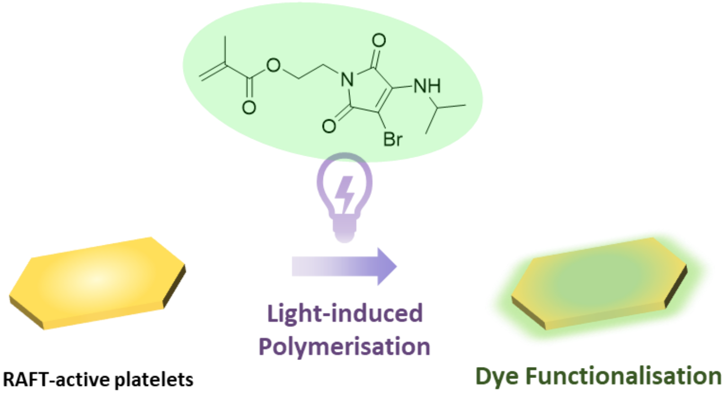

Scheme S6. Strategy of surface fluorescence modification of platelet via light induced polymerization (photo iniferter polymerization) with aminobromomaleimide methacrylate (ABMMA) based dye monomer.

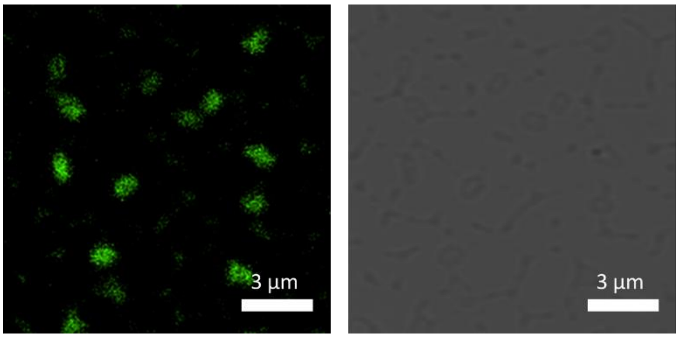

Figure S31. CLSM images of platelets after photo iniferter polymerization with fluorescent dye (methacrylate aminobromomaleimide) on 2D platelets assemblies. Left: confocal field; Right: bright field. (Scale bar = 3 µm)

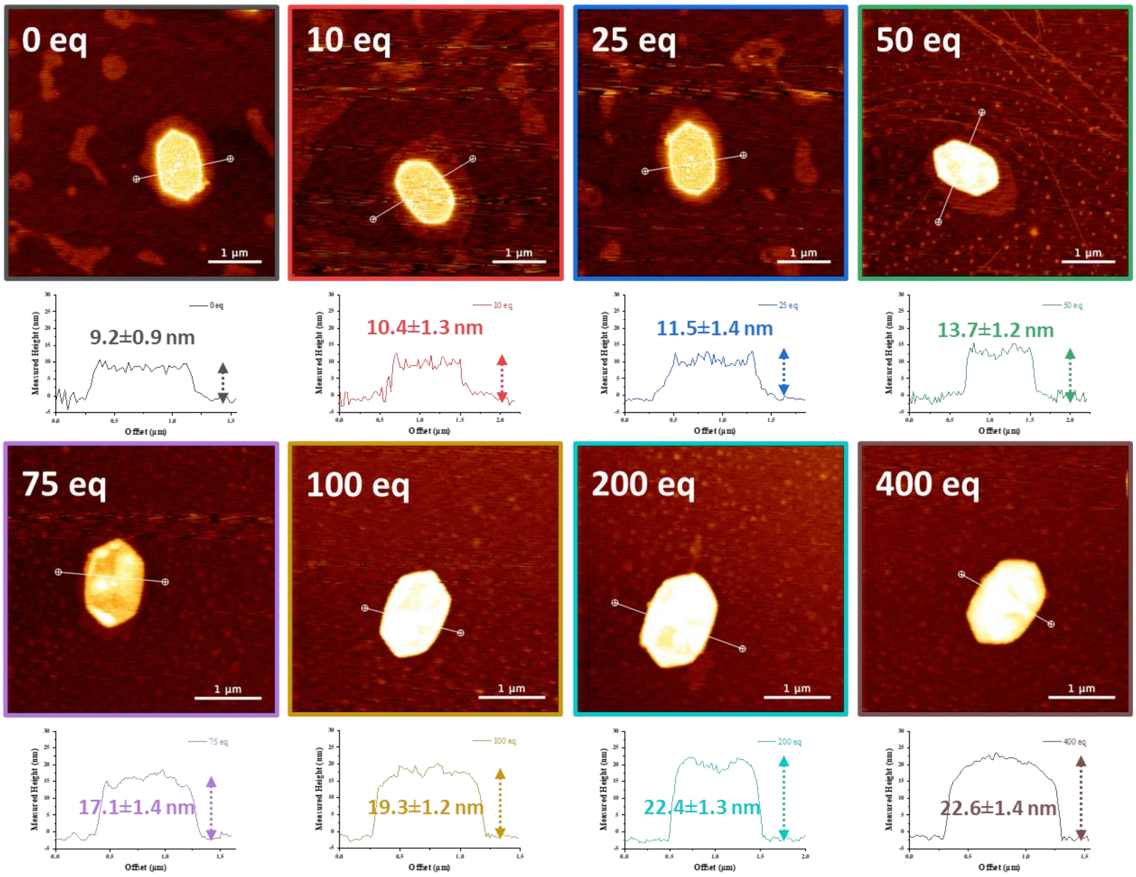

Figure S32. AFM images of platelets reacted with different concentration of DMA (0, 10, 25, 50, 75, 100, 200 and 400 mass equivalents to platelets mass) after photo-iniferter polymerzation.

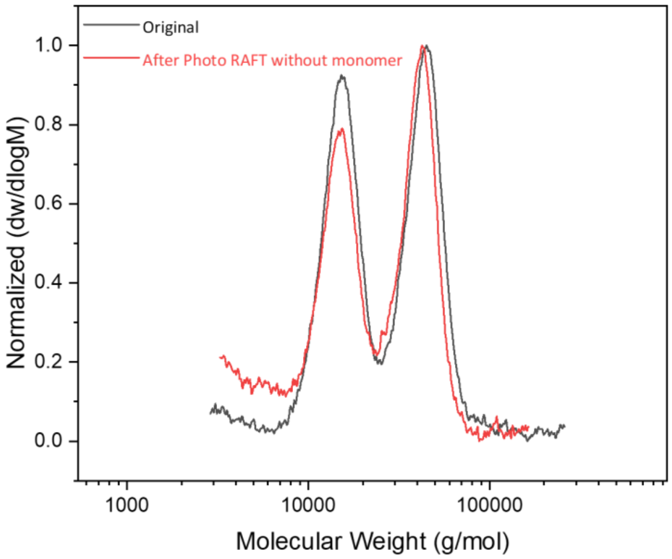

Figure S33. SEC chromatogram of platelets reacted without addition of DMA before and after photo-iniferter polymerzation.

To explore the possibility of interlinking between neighboring chains. A control experiment in which platelet solution was used without the additional DMA monomer. This scenario provides the greatest opportunity for interlinking between neighboring chains. If so, a new peak of higher molecular weight will occur. However, from SEC characterization, no right shift of molecular weight was observed and two RI trace almost overlapped. Thus, we think the amount of interlinking between the neighboring chains is very low.

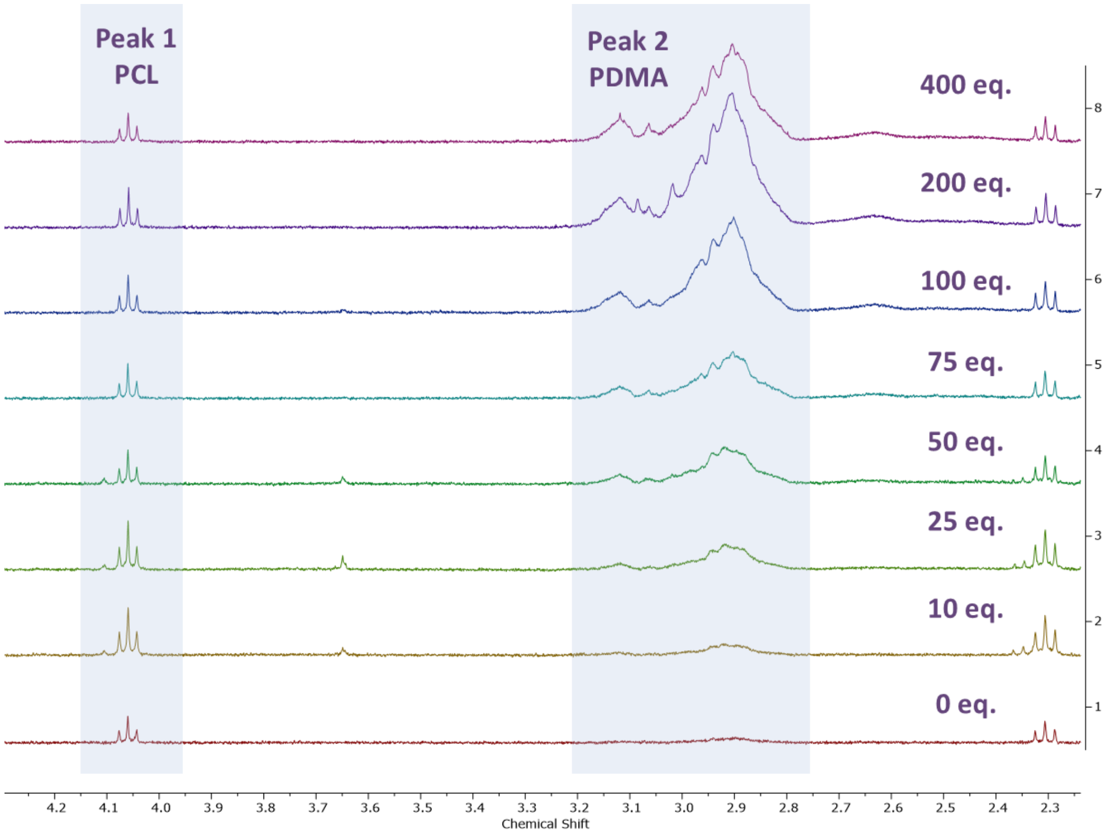

Figure S34. ^{1}H NMR analysis of platelets reacted with different concentration of DMA (0, 10, 25, 50, 75, 100, 200 and 400 mass equivalents to platelets mass) after photo-iniferter polymerization.

Table S3. The change of degree of polymerization (ΔDP), molecular weight (ΔM_w) and platelets height (Δh) after photo-iniferter polymerzation under different concentration of DMA (0, 10, 25, 50, 75, 100, 200 and 400 mass equivalents to platelets mass).

DMA concentration	0 equiv	10 equiv	25 equiv	50 equiv	75 equiv	100 equiv	200 equiv	400 equiv
ΔDP (average)	0	18	40	116	197	280	435	473
ΔM_w (kg/mol, average)	0	1.7	3.9	11.5	19.5	27.7	43.1	46.8
Δh (nm, average)	0	1.2	2.3	4.4	7.8	10.0	13.1	13.3

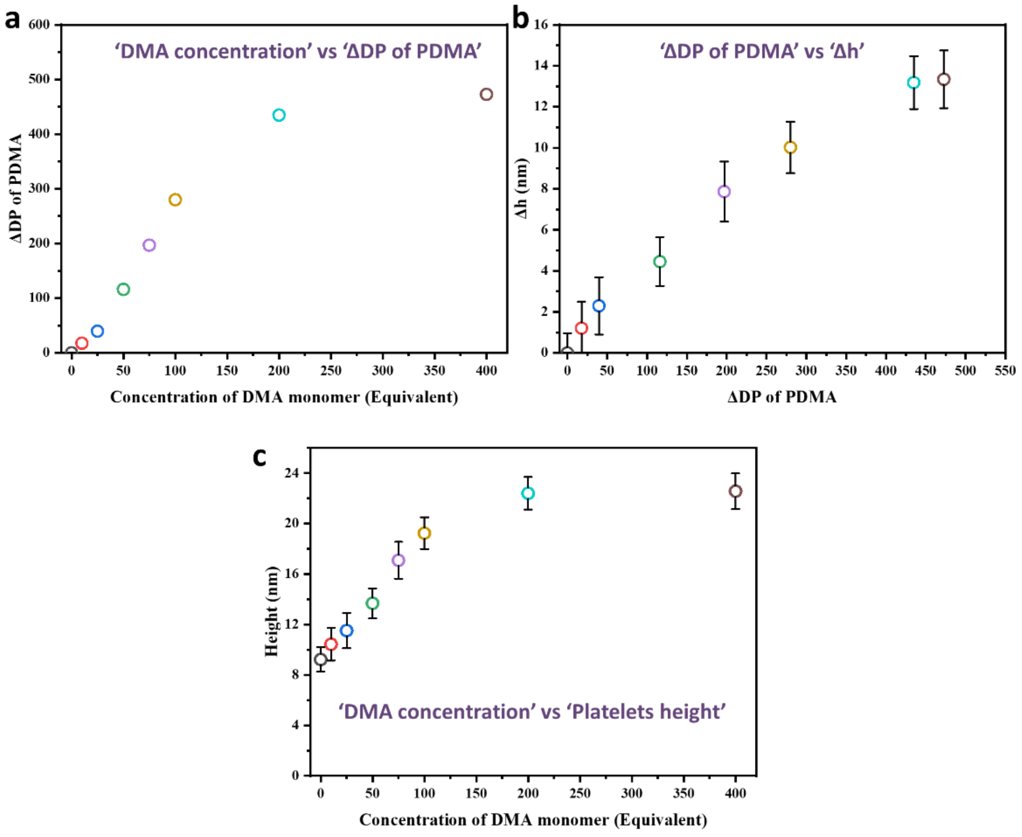

Figure S35. The relationships between three key factors: (a) DMA concentration and 'increasing DP of PDMA block; (b) increasing DP of PDMA block and increasing height; (c) DMA concentration and platelet height after photo-iniferter polymerzation under different concentration of DMA (0, 10, 25, 50, 75, 100, 200 and 400 mass equivalents to platelets mass).

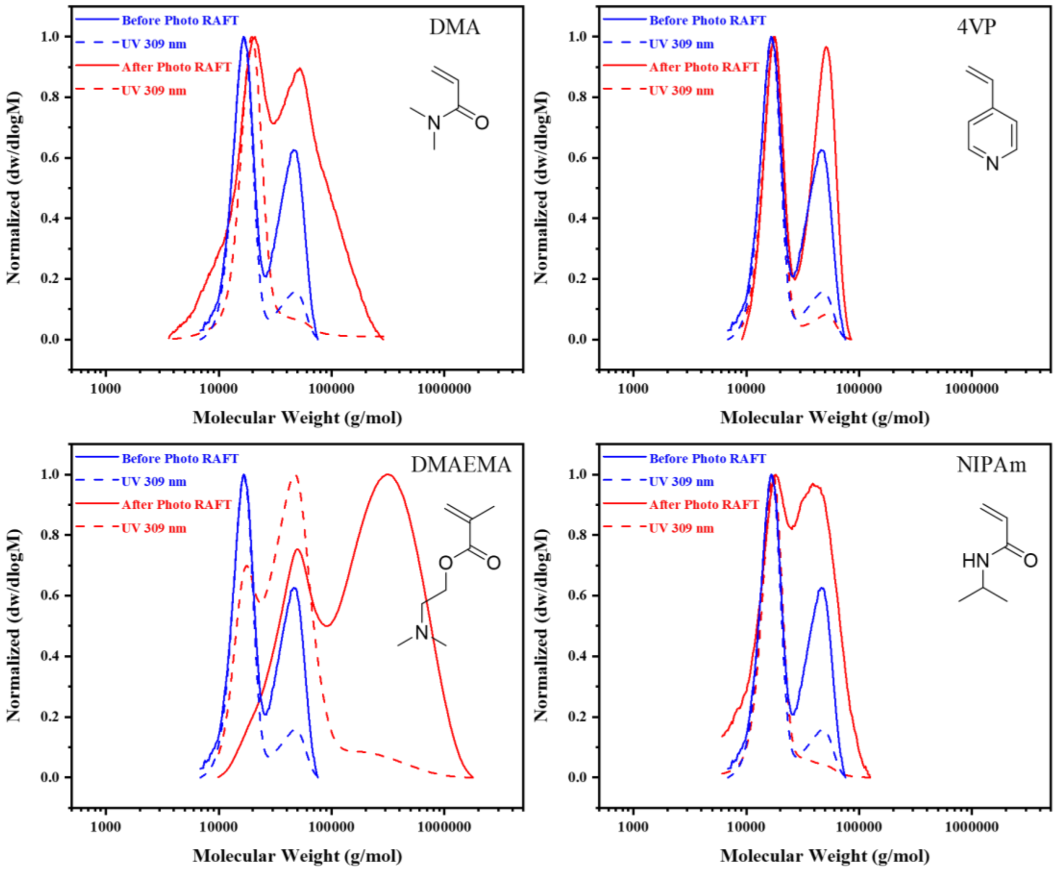

Figure S36. SEC chromatogram of PCL/PCL-*b*-PDMA platelets reacted with different monomer DMA, NIPAm, 4VP, and DMAEMA before and after photo-iniferter polymerization using $CHCl_3$ with 0.5% TEA as an eluent with PMMA standards.

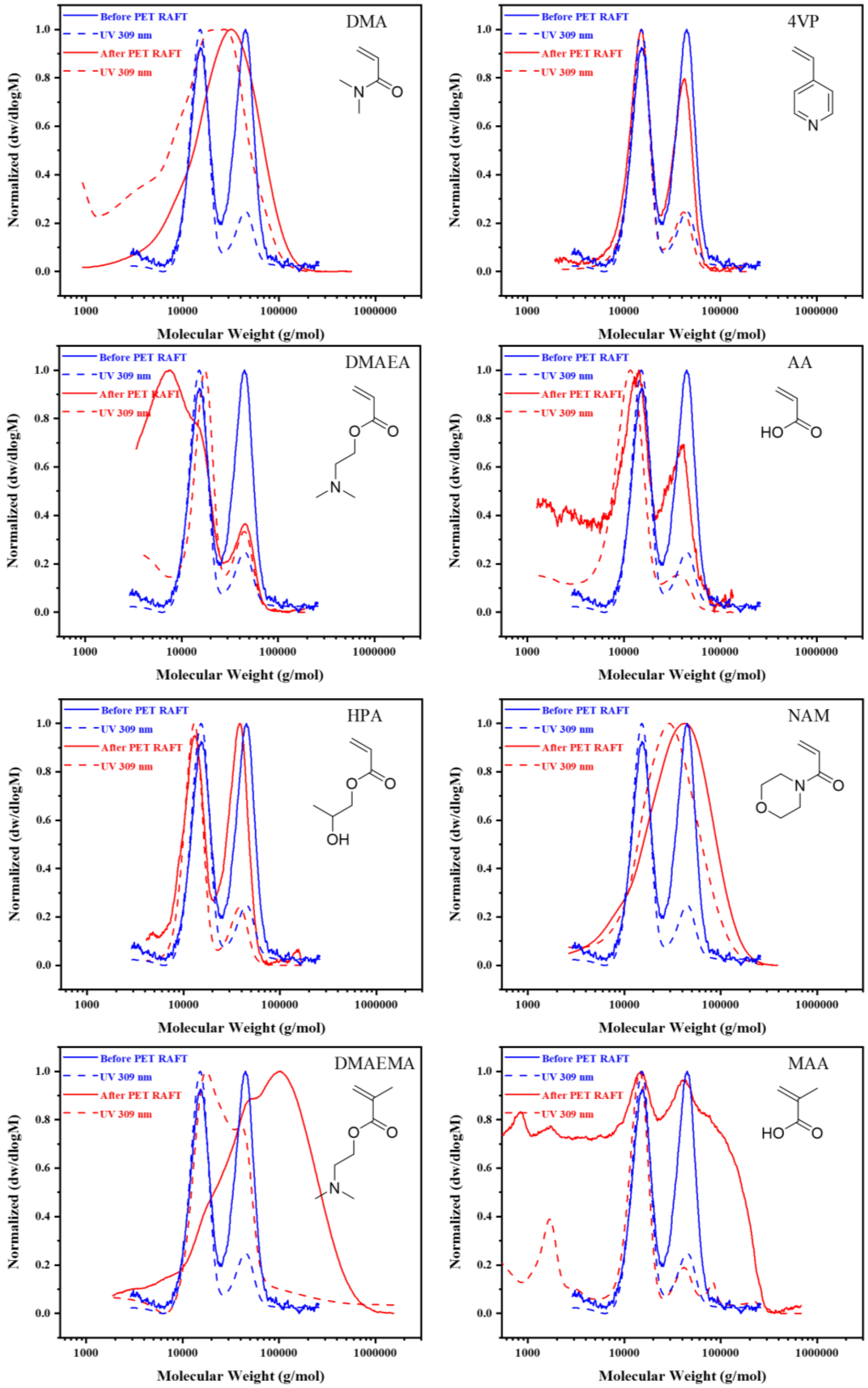

Figure S37. SEC chromatogram of PCL/PCL-*b*-PDMA platelets reacted with different monomer DMA, 4VP, DMAEA, AA, HPA, NAM, DMAEMA, and MAA before and after PET-RAFT polymerization using $CHCl_3$ with 0.5% TEA as an eluent with PMMA standards.

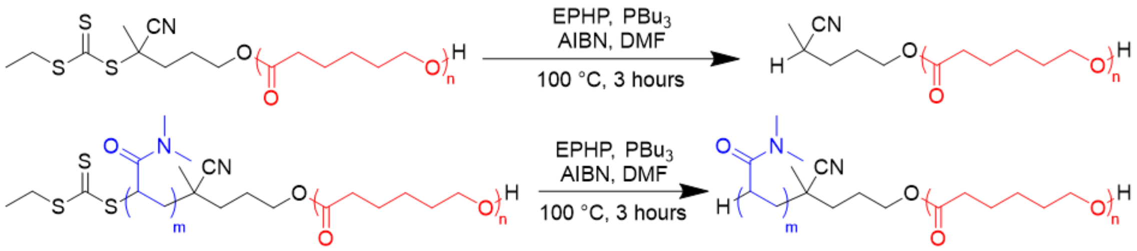

Scheme S7. Design of RAFT group removed homopolymer and block copolymer. The trithiocarbonate group removal was conducted by (N-ethylpiperidine hypophosphite) EPHP method, which loses chain extended ability when radical polymerization is initiated.[12]

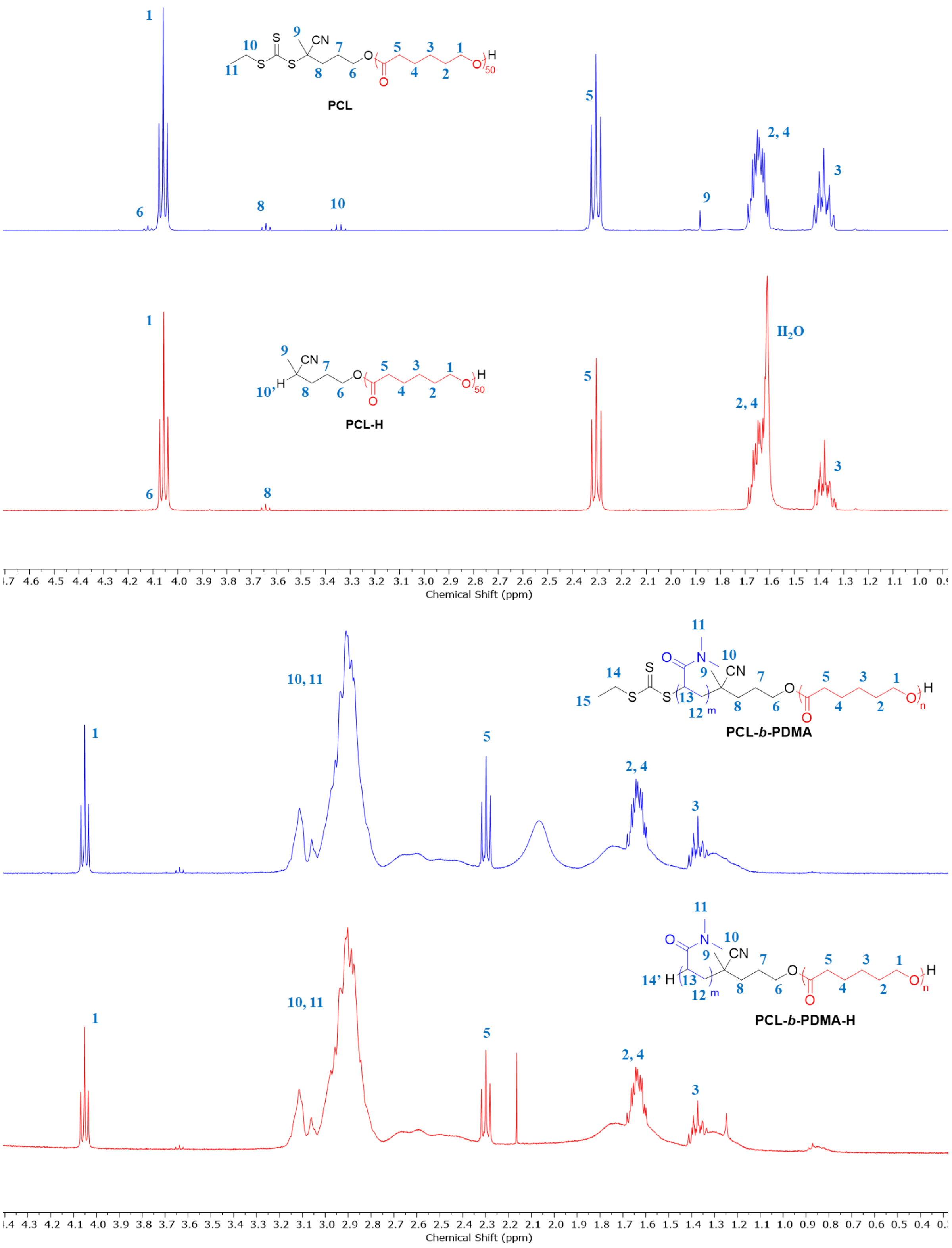

Figure S38. ^{1}H NMR spectrum of homopolymer PCL_{50} and block copolymer PCL_{50}-*b*-$PDMA_{198}$ before and after removing end group in $CDCl_3$ (400 MHz).

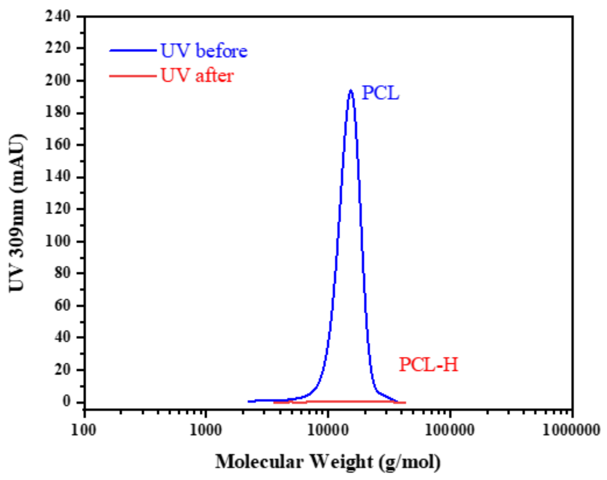

Figure S39. SEC chromatogram (UV trace, 309 nm) of PCL_{50} homopolymer before and after removing end group using $CHCl_3$ with 0.5% TEA as an eluent with PMMA standards.

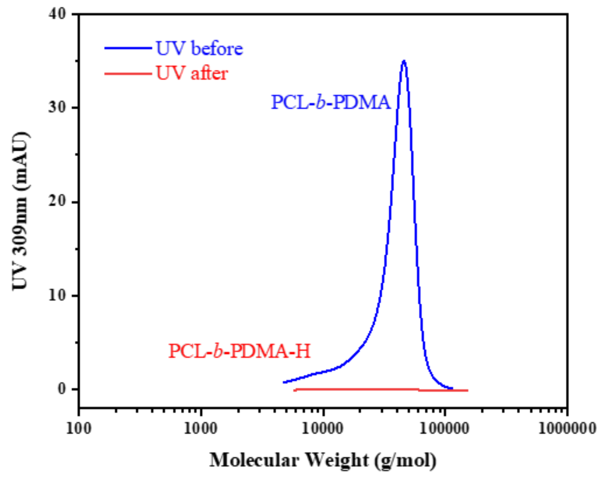

Figure S40. SEC chromatogram (UV trace, 309 nm) of block copolymer PCL_{50}-*b*-$PDMA_{198}$ before and after removing end group using $CHCl_3$ with 0.5% TEA as an eluent with PMMA standards.

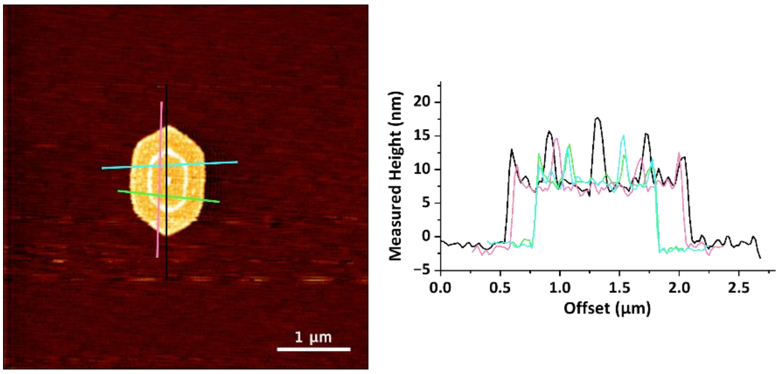

Figure S41. AFM images and height profiles of double-layered platelet (inner layer: RAFT active layer, outer layer: RAFT inactive layer) before photoiniferter polymerization with DMA monomer.

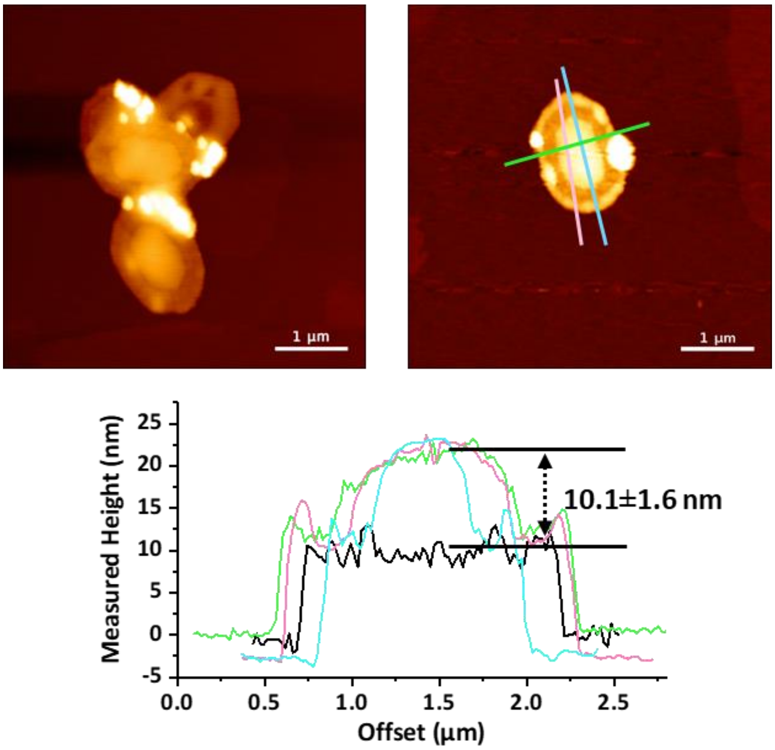

Figure S42. AFM images and height profiles of double-layered platelet (inner layer: RAFT active layer, outer layer: RAFT inactive layer) after photoiniferter polymerization with DMA monomer.

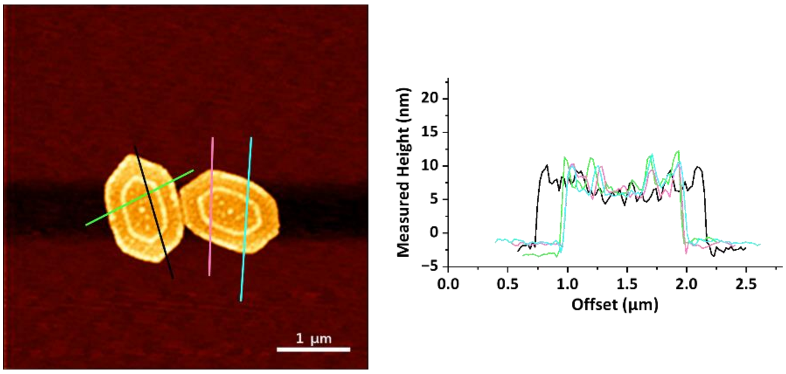

Figure S43. AFM images and height profiles of double-layered platelet (inner layer: RAFT inactive layer, outer layer: RAFT active layer) before photoiniferter polymerization with DMA.

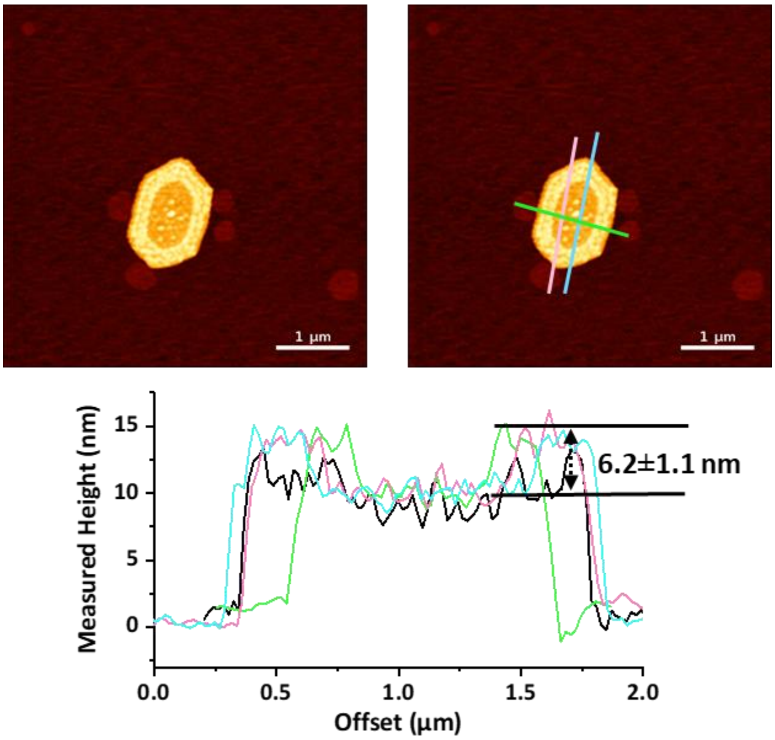

Figure S44. AFM images and height profiles of double-layered platelet (inner layer: RAFT inactive layer, outer layer: RAFT active layer) after photoiniferter polymerization with DMA.

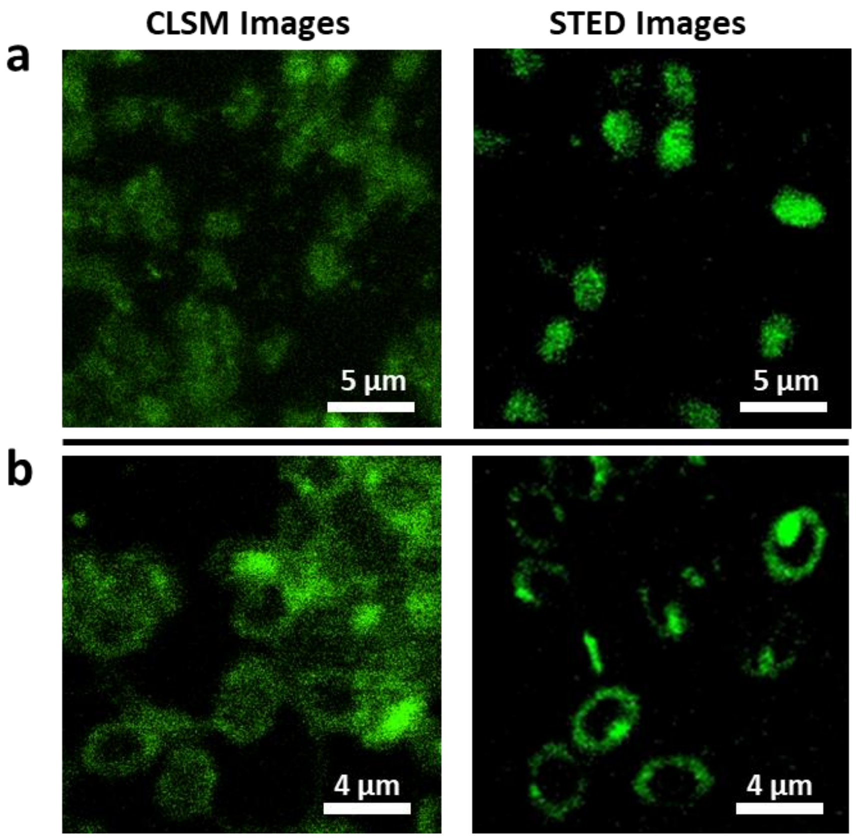

Figure S45. CLSM and STED images of double-layered platelet after photoiniferter polymerization with fluorescent dye. (a) inner layer: RAFT active layer, outer layer: RAFT inactive layer; (b) inner layer: RAFT inactive layer, outer layer: RAFT active layer.

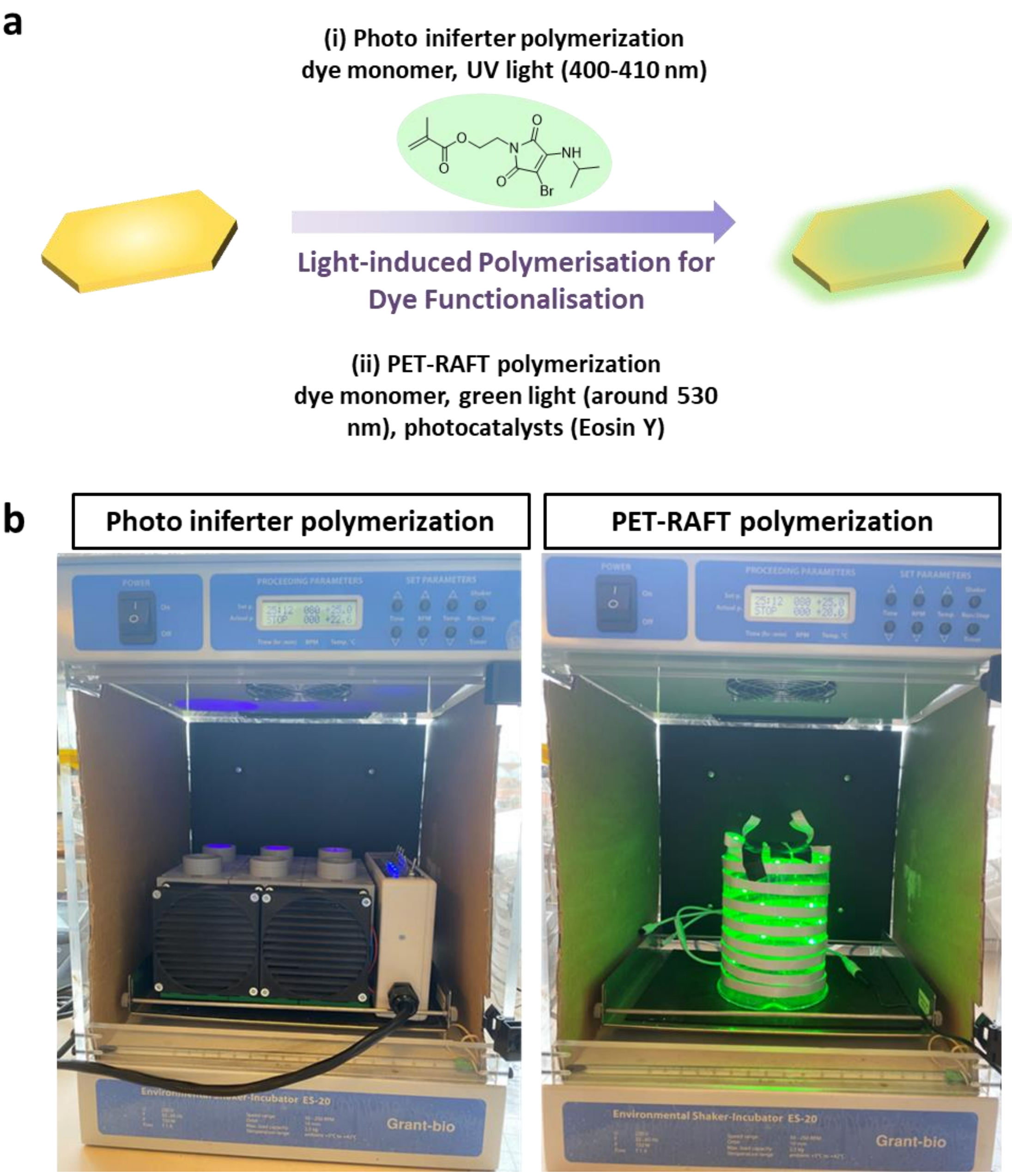

Figure S46. (a) Strategy of surface dye functionalization of platelet via light induced polymerization ((i) photoiniferter polymerization and (ii) PET-RAFT polymerization) with dye monomer. (b) Apparatus setup for light induced polymerization.

Reference:

1. Y. Xie, J. T. Husband, M. Torrent-Sucarrat, H. Yang, W. Liu and R. K. O'Reilly, *Chem. Commun.*, 2018, **54**, 3339-3342.
2. Y. Xie, M. C. Arno, J. T. Husband, M. Torrent-Sucarrat and R. K. O'Reilly, *Nat. Commun.*, 2020, **11**, 2460.
3. J. Schmelz, M. Karg, T. Hellweg and H. Schmalz, *ACS Nano*, 2011, **5**, 9523-9534.
4. H. U. Kang, Y. C. Yu, S. J. Shin and J. H. Youk, *J. Polym. Sci., Part A: Polym. Chem.*, 2012, **51**, 774-779.
5. S. Varlas, P. G. Georgiou, P. Bilalis, J. R. Jones, N. Hadjichristidis and R. K. O'Reilly, *Biomacromolecules*, 2018, **19**, 4453-4462.
6. W. Yu, M. Inam, J. R. Jones, A. P. Dove and R. K. O'Reilly, *Polym. Chem.*, 2017, **8**, 5504-5512.
7. Z. Tong, Y. Li, H. Xu, H. Chen, W. Yu, W. Zhuo, R. Zhang and G. Jiang, *ACS Macro Lett.*, 2016, **5**, 867-872.
8. H. Qiu, Y. Gao, C. E. Boott, O. E. Gould, R. L. Harniman, M. J. Miles, S. E. Webb, M. A. Winnik and I. Manners, *Science*, 2016, **352**, 697-701.
9. J. Wang, W. Zhu, B. Peng and Y. M. Chen, *Polymer*, 2013, **54**, 6760-6767.
10. M. Inam, G. Cambridge, A. Pitto-Barry, Z. P. L. Laker, N. R. Wilson, R. T. Mathers, A. P. Dove and R. K. O'Reilly, *Chem. Sci.*, 2017, **8**, 4223-4230.
11. L. Liu, L. Zhu, Z. Chu and Z. Tong, *Macromolecules*, 2023, **56**, 5984-5992.
12. H. Willcock and R. K. O'Reilly, *Polym. Chem.*, 2010, **1**, 149-157.
